# Correction: Phytochemical profiling of *Vitex negundo* seeds via UHPLC-QTOF-MS/MS analyses with antimicrobial evaluation and in silico targeting of DNA Gyrase B and Secreted Aspartic Proteinase 2 (SAP2)

**DOI:** 10.1371/journal.pone.0349766

**Published:** 2026-05-22

**Authors:** Javed Mustafa, Tuba Ashraf, Basharat Ali, Shazia Kousar, Adeem Mahmood, Saif Ullah, Muhammad Imran, Bakhat Ali, Usman Rahim, Muhammad Yunis

The seventh author, Muhammad Imran, should not be noted as the corresponding author.
