# Supplementary material for: Phytochemical profiling of Vitex negundo seeds via UHPLC-QTOF-MS/MS analyses with antimicrobial evaluation and in silico targeting of DNA Gyrase B and Secreted Aspartic Proteinase 2 (SAP2)
Source: PLoS One. 2026 Mar 13;21(3):e0343965. doi: 10.1371/journal.pone.0343965 (PMC12987476; doi:10.1371/journal.pone.0343965)
Supplement: S1 File — S1. Antibacterial assay; S2. Antifungal assay. S3. UHPLC-QTOF-MS/MS-based metabolic profiling of Vitex negundo seeds in positive ionization mode procedure; S4 – S8. Description of Compound 11 – Compound 15; S9. Fig 5 and Fig 6. Docking simulations of major bioactive constituents. (DOCX) [file pone.0343965.s001.docx]

**Phytochemical Profiling of *Vitex negundo* Seeds via UHPLC-QTOF-MS/MS Analyses with Antimicrobial Evaluation and In Silico Targeting of DNA Gyrase B and Secreted Aspartic Proteinase 2** (**SAP2)**

**Javed Mustafa^1^, Tuba Ashraf^1^, Basharat Ali^1^, Shazia Kousar^1^, Adeem Mahmood^2^, Muhammad Imran^3^*, Saifullah^4^,** **Usman Rahim^1^, Muhammad Younis^1^, Bakhat Ali^1,5^***

^1^Institute of Chemistry, Khwaja Fareed University of Engineering & Information Technology, Rahim Yar Khan, 64200, Pakistan

^2^John Innes Centre, Norwich Research Park, NR4 7UH, United Kingdom

^3^Department of Chemistry, College of Science, King Khalid University, 61413, Abha, Saudi Arabia

^4^Institute of Chemistry, Govt Sadiq Abbas Graduate College, Dera Nawab Sahib, District

Bahawalpur, 62020, Pakistan

^5^International Center for Chemical and Biological Sciences, H.E.J. Research Institute of

Chemistry, University of Karachi, Karachi-75270, Pakistan

*Corresponding Authors: bakhat.ali@kfueit.edu.pk, imranchemist@gmail.com

**Supporting information**

**S1** **(Antibacterial assay)**

Colonies of *Staphylococcus aureus* were initially cultured in a broth medium comprising 10^6^ cells/ml, in a hemocytometer (China-Quijing). Later, to promote colony development, 100µl of the bacterial suspension was incubated at 37°C for 24 hours. A small portion of bacterial growth was blended and extracted with high-nutrient broth for 30 minutes, after which sterile cotton swabs were used to transfer bacterial colonies onto agar plates [1, 2]. Four wells were created in each agar plate using sterile cork borers, and 1 µL of molten agar medium was added to each well. Molten agar nutrients (1µl) have been added into each well during filling. The agar nutrients were allowed to solidify in each well. After this, different methanolic seed extract fractions of *V. negundo* were introduced into the wells for antimicrobial evaluation [3].

**S2** **(Antifungal assay)**

The methanolic seed extract of *Vitex negundo* was evaluated against the *Candida albicans* strain. The broth nutrients of 13 g/L combined with cleansed water have been employed to revitalize fungal strains. The broth was thoroughly autoclave-sterilized for this purpose before being transferred to a 250 ml flask. The flask was subsequently inoculated with a fresh fungal strain. The flask was incubated for three days in the incubator at 30°C to allow fungal growth. The methanolic seed extract was prepared at concentrations of 0.15 mg/mL (low dilution, TL) and 0.30 mg/mL (high dilution, TH). For this activity, amphotericin (reference) was used with various fractions, comparable to plant extracts, at concentrations of 0.30 mg/ml and 0.15 mg/ml. A sterile cotton swab was used to transfer the fungal strain onto an Agar plate of Petri dishes, and wells were loaded with different seed extract concentrations. The Agar plates of a fungal strain were incubated at 30°C for three days. Each sample dosage suppresses the fungal strain, and its zone of inhibition was measured in millimeters to evaluate antifungal efficacy [4].

**Fig (S1, S2). Antibacterial and antifungal activity of MeOH seed extract of *V. negundo*.**

**S3** **(UHPLC-QTOF-MS/MS-based metabolic profiling of *Vitex negundo* seeds in positive ionization mode)**

The Sciex X500 QTOF mass spectrometer with high-resolution analysis was used with a precision error of less than 4 ppm. The mass spectrometer was coupled with electrospray ionization (ESI) and operated in positive ionization mode, having a collision energy of 35 ± 15 eV for mass fragmentation. The mass-to-charge ratio (m/z) was set between 40 to 2000 to allow the comprehensive mass fragmentation analysis. The instrument acquired five spectra per second with the help of MS/MS fragmentation mode, systematized to acquire two precursor values within one cycle. In this study, Sciex Peak View version 2.10 and MS/MS fragmented software with the support from ACD Labs were used for data analysis. The exhaustive analysis of a total of 17 bioactive compounds was identified and characterized by an ion chromatogram as outlined in Fig 1 and Table 1. The study presents comprehensive confirmations of identified metabolites, consisting of their peak heights, molecular formulas, Rt (retention time), covered peak areas, precursor m/z, and authenticated from previous literature and internet databases. The identified compounds were classified into different biological classes, like lignins, flavonoids, phenolic glycosides, phenolic acids, amino acids, fatty acids, and triterpenes. Some peaks of bioactive compounds in the ion chromatogram were still assigned as unknown in the *Vitex negundo* seed extract.

**S4**

**Compound (11)** was identified with a retention time (Rt) of 2.11 min, eluted under positive mode of ionization, exhibiting a precursor ion at m/z 440 [M+H]^+^, corresponding to the molecular formula C_30_H_62_. Two prominent fragment ions with high intensities were observed at m/z 85 [M+H-355]^+^, resulting from cleavage between two carbon atoms of the bioactive compound, and representing a radical with a molecular formula C_24_H_49_**^˙^**. An additional, less intense fragment appeared at m/z 127 [M+H-313]^+^ corresponding to formula C_21_H_43_**^˙^**. Additional fragments appeared in the MS/MS spectrum but with less intense values, for example, at m/z 145 [M+H-295]^+^ and 163 [M+H-277]^+^. The compound was identified as triacontane, shown in Fig 2 and Table 1 [5].

**S5**

**Compound (12)** was detected with retention time (Rt) of 1.51 min in positive ionization mode, showing a precursor ion at m/z 408 [M+H]^+^, corresponding to the molecular formula C_29_H_60_. The MS/MS spectrum revealed several fragment ions, with the most intense peak observed at m/z 85 [M+H-323]^+^, resulting from cleavage between two carbon atoms of the long-chain hydrocarbon. Additional fragments were detected at m/z 145 [M+H-263]^+^, 163 [M+H-245]^+^, and 229 [M+H-179]^+^ respectively. Comparison with fragmentation pattern and online literature confirmed the compound as nonacosane (Fig 2 and Table 1) [6].

**S6**

**Compound (13)** was detected with a retention time (Rt) of 3.53 min in positive ionization mode [M+H]^+^, showing a precursor ion at m/z 464 [M+H]^+^ corresponding to the molecular formula C_33_H_68_. The dominant peak was observed at m/z 127 [M+H-337]^+^, resulting from cleavage between two carbon atoms and the formation of a radical ion C_24_H_49_˙. The additional fragments were also detected at m/z 85 [M+H-379]^+^, 207 [M+H-257]^+^, and 271 [M+H-193]^+^ corresponding to cleavages at various positions along the hydrocarbon chain. Thus, comparison of the fragmentation pattern with online literature, the molecule was identified as tritriacontane, shown in Fig 2 and Table 1 [7].

**S7**

**Compound (14)** was identified with a retention time (Rt) of 2.33 min, showing a precursor ion at m/z 278 [M+H]^+^ in positive ionization mode, corresponding to the molecular formula as C_18_H_30_O_2_. A characteristic fragment was identified at m/z 133 [M+H-145]^+^, resulting from cleavage at two sites: one by removing a proton [H^+^] and elimination of the radical C_8_H_15_O_2_**^˙^**. Additional fragments were also detected at m/z 157 [M+H-121]^+^ and 60 [M+H-218]^+^. Thus, the compound was identified by comparison with online literature with a fragmentation pattern as linolenic acid (Fig 2 and Table 1) [8].

**S8**

**Compound (15)** was characterized using the LC-MS/MS technique, with an expected molecular formula of C_19_H_30_O_4_ and eluted at a retention time (Rt) of 5.27 min. The MS/MS spectrum displayed a precursor ion at m/z 322 [M+H]^+^ in a positive ionization mode (Fig 2 and Table 1). The most intense fragment appeared at m/z 262 [M+H-60]^+^, resulting from multiple cleavages involving the loss of a proton (H^+^), an oxygen atom, and a C_2_H_3_O˙ radical. Fragment ion was detected at m/z 84 [M+H-238]^+^ attributed to the elimination of the compound C_15_H_26_O_2_**^˙^** moiety through dual carbon–carbon bond cleavage. Furthermore, additional fragments were detected at m/z 217 [M+H-105]^+^, 140 [M+H-182]^+^, and 58 [M+H-264]^+^. Thus, based on comparison with literature data, the compound was identified as vitedoin B [9].

**S9** **(Docking simulations of major bioactive constituents as antibacterial and antifungal agents)**

**Fig 5. (a) The 3D-crystal structure of protein 4URO; b 3D interaction of isoorientin docking with 4URO; c 2D interaction of isoorientin docking with 4URO; d hydrophobic interaction of isoorientin with 4URO**.

**Fig 5. (b) The 3D-crystal structure of protein 4URO; b 3D interaction of quercetin docking with 4URO; c 2D interaction of quercetin docking with 4URO; d hydrophobic interaction of quercetin with 4URO.**

**Fig 5. (c) The 3D-crystal structure of protein 4URO; b 3D interaction of orientin docking with 4URO; c 2D interaction of orientin docking with 4URO; d hydrophobic interaction of orientin with 4URO.**

**Fig 6. (a) The 3D-crystal structure of protein 1EAG; b 3D interaction of isoorientin docking with 1EAG; c 2D interaction of isoorientin docking with 1EAG; d hydrophobic interaction of isoorientin with 1EAG.**

**Fig 6. (b) The 3D-crystal structure of protein 1EAG; b 3D interaction of quercetin docking with 1EAG; c 2D interaction of quercetin docking with 1EAG; d hydrophobic interaction of quercetin with 1EAG.**

**Fig 6. (c) The 3D-crystal structure of protein 1EAG; b 3D interaction of orientin docking with 1EAG; c 2D interaction of orientin docking with 1EAG; d hydrophobic interaction of orientin with 1EAG**.

**References**

1. Zargar M, Hamid AA, Bakar FA, Shamsudin MN, Shameli K, Jahanshiri F, Farahani F. Green Synthesis and Antibacterial Effect of Silver Nanoparticles Using Vitex Negundo L. Molecules 2011; 16 (8): 6667–6676. <https://doi.org/10.3390/molecules16086667>

2. Yeo YL, Chia YY, Lee CH, Sow HS, Yap WS. Effectiveness of Maceration Periods with Different Extraction Solvents on In-Vitro Antimicrobial Activity from Fruit of *Momordica Charantia* L. J. Appl. Pharm. Sci. 2014; 4 (10); 016–023. <https://doi.org/10.7324/JAPS.2014.401004>

3. Sen A, Batra A. Evaluation of Antimicrobial Activity of Different Solvent Extracts of Medicinal Plant: Melia Azedarach L. Int J Curr Pharm Res 2012; 4 (2): 67–73.

4. Ullah N, Rehman A, Ahmad S, Samad N, Andaleeb H, Ahmad W, Ahmad K, Haroon M, Maqbool S, Altaf A. Antimicrobial Assay and Minimum Inhibitory Concentration Values of *Cistanche tubulosa*. Int. J. Curr. Microbiol. Appl. Sci. 2016; 5 (2): 380–388. <http://dx.doi.org/>10.205 46/ijcmas.2016.502.043

5. Kumar M, Prakash S, Radha, Kumari N, Pundir A, Punia S, Saurabh V, Choudhary P, Changan S, Dhumal S. Beneficial Role of Antioxidant Secondary Metabolites from Medicinal Plants in Maintaining Oral Health. Antioxidants 2021; 10 (7): 1061. https://doi.org/10.3390/antiox10071061

6. Meena AK, Perumal A, Kumar N, Singh R, Ilavarasan R, Srikanth N, Dhiman KS. Studies on Physicochemical, Phytochemicals, Chromatographic Profiling & Estimation and in-Silico Study of Negundoside in Roots & Small Branches of Vitex Negundo Plant. Phytomedicine Plus 2022; 2 (1): 100205. https://doi.org/10.1016/j.phyplu.2021.100205

7. Perveen S, Khan MA, Parveen R, Insaf A, Parveen B, Ahmad S, Husain SA. An Updated Review on Traditional and Modern Aspects of Vitex Negundo. Curr. Tradit. Med. 2023; 9 (2): 114–127. https://doi.org/10.2174/2215083808666220827115915

8. Kannathasan K, Senthilkumar A, Venkatesalu V, Chandrasekaran M. Larvicidal Activity of Fatty Acid Methyl Esters of Vitex Species against Culex Quinquefasciatus. Parasitol. Res. 2008; 103 (4): 999–1001. https://doi.org/10.1007/s00436-008-1078-1

9. Zheng CJ, Li HQ, Ren SC, Xu CL, Rahman K, Qin LP, Sun YH. Phytochemical and Pharmacological Profile of *Vitex Negundo*. Phytotherapy Research 2015; 29 (5): 633–647. <https://doi.org/10.1002/ptr.5303>
