# Supplementary figures and images for: Phytochemical profiling of Vitex negundo seeds via UHPLC-QTOF-MS/MS analyses with antimicrobial evaluation and in silico targeting of DNA Gyrase B and Secreted Aspartic Proteinase 2 (SAP2)
Source: PLoS One. 2026 Mar 13;21(3):e0343965. doi: 10.1371/journal.pone.0343965 (PMC12987476; doi:10.1371/journal.pone.0343965)

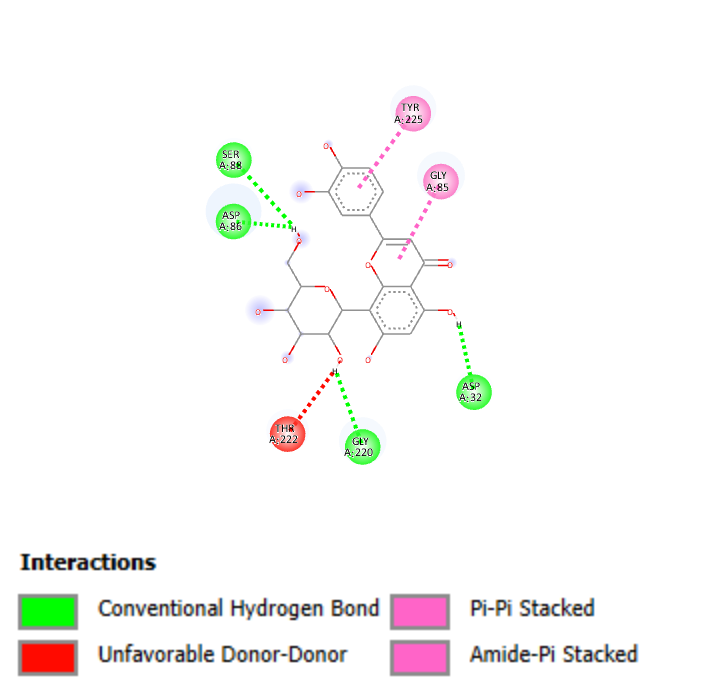

Supplement: S4 Data — (ZIP) [file pone.0343965.s005.zip › PONE-D-25-51583/Vitex Raw material/Docking 3, 1EAG/molecule4/1eag-f17.png]

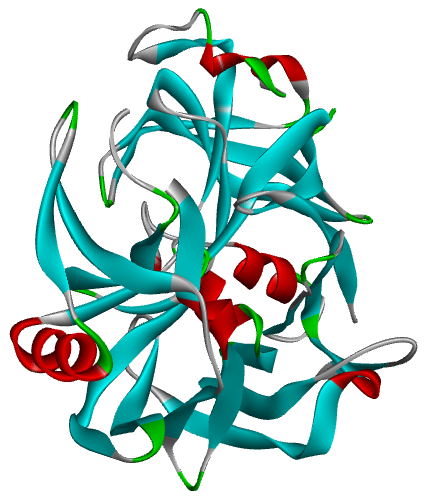

Supplement: S4 Data — (ZIP) [file pone.0343965.s005.zip › PONE-D-25-51583/Vitex Raw material/Docking 3, 1EAG/molecule4/1eag.png]

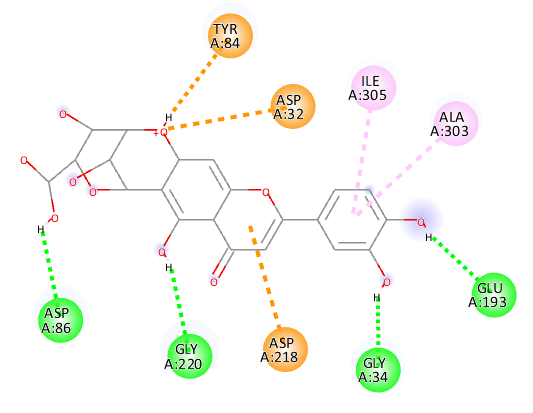

Supplement: S4 Data — (ZIP) [file pone.0343965.s005.zip › PONE-D-25-51583/Vitex Raw material/Docking 3, 1EAG/molecule4/f4-c4.png]

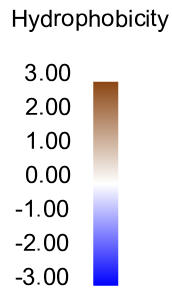

Supplement: S4 Data — (ZIP) [file pone.0343965.s005.zip › PONE-D-25-51583/Vitex Raw material/Docking 3, 1EAG/molecule4/ff4.png]

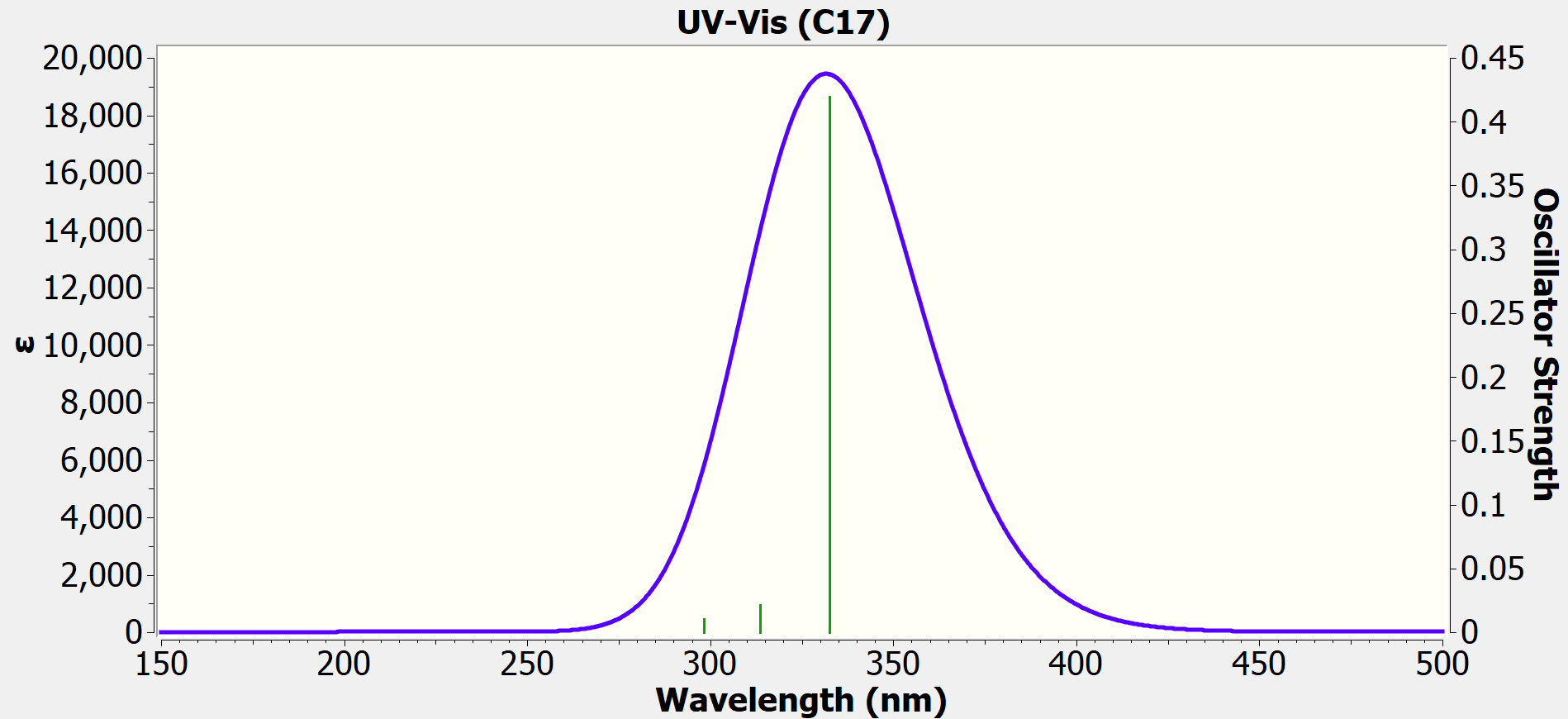

Supplement: S5 Data — (ZIP) [file pone.0343965.s006.zip › PONE-D-25-51583/Vitex Raw material/DFT Vitex all data/C17UV/33.png]

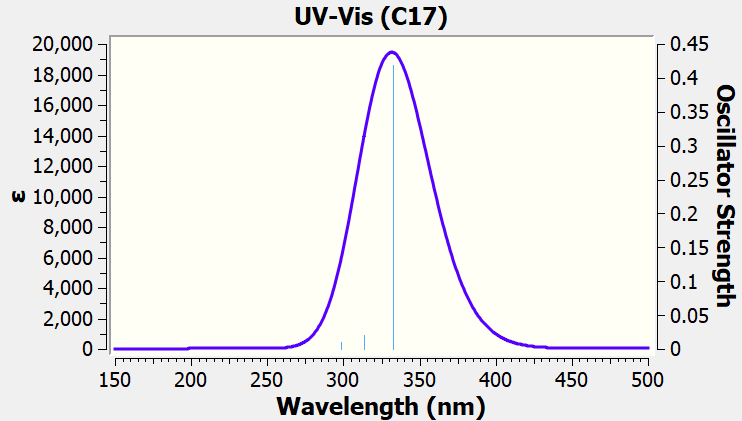

Supplement: S5 Data — (ZIP) [file pone.0343965.s006.zip › PONE-D-25-51583/Vitex Raw material/DFT Vitex all data/C17UV/ORIENTIN22_uvvis.png]

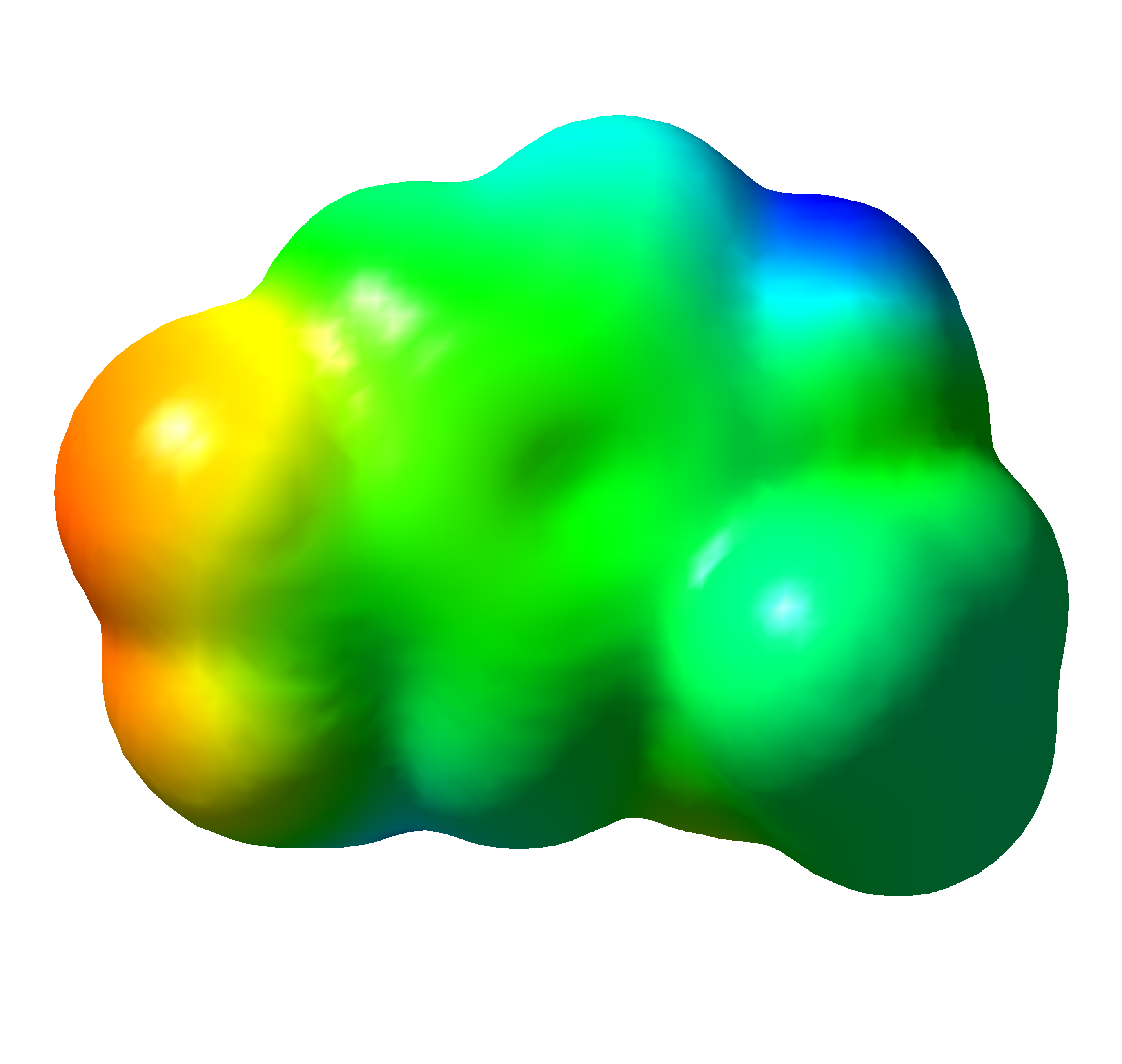

Supplement: S5 Data — (ZIP) [file pone.0343965.s006.zip › PONE-D-25-51583/Vitex Raw material/DFT Vitex all data/comp1/comp1.tif]

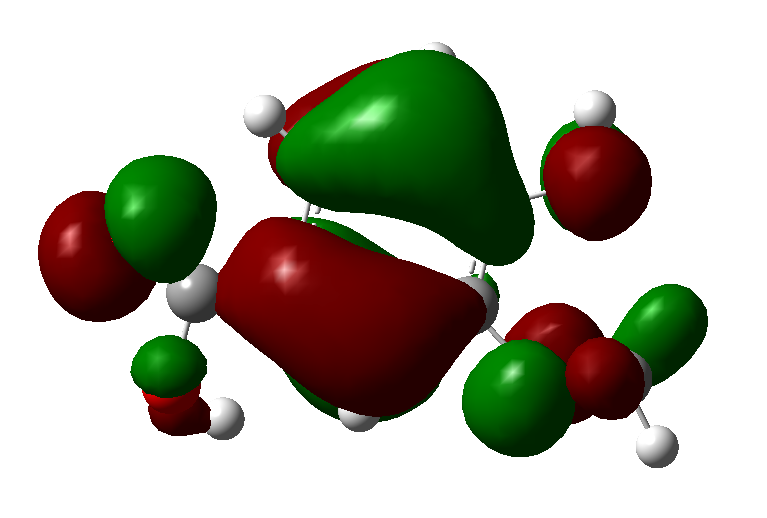

Supplement: S5 Data — (ZIP) [file pone.0343965.s006.zip › PONE-D-25-51583/Vitex Raw material/DFT Vitex all data/comp1/homo-1.tif]

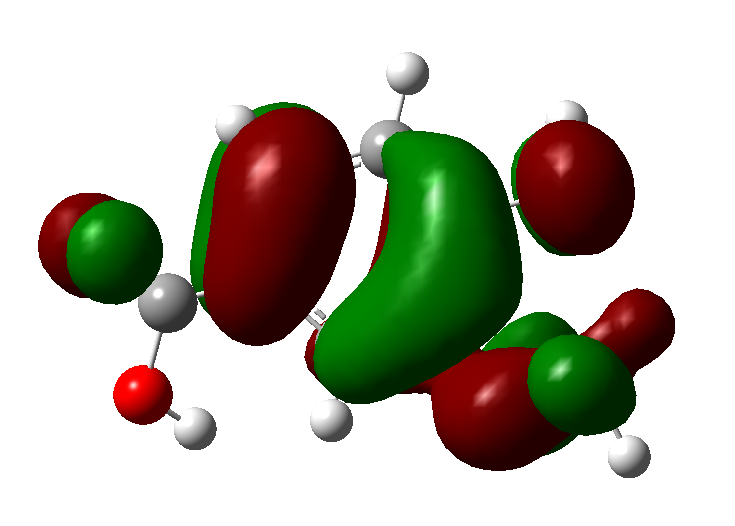

Supplement: S5 Data — (ZIP) [file pone.0343965.s006.zip › PONE-D-25-51583/Vitex Raw material/DFT Vitex all data/comp1/homo.tif]

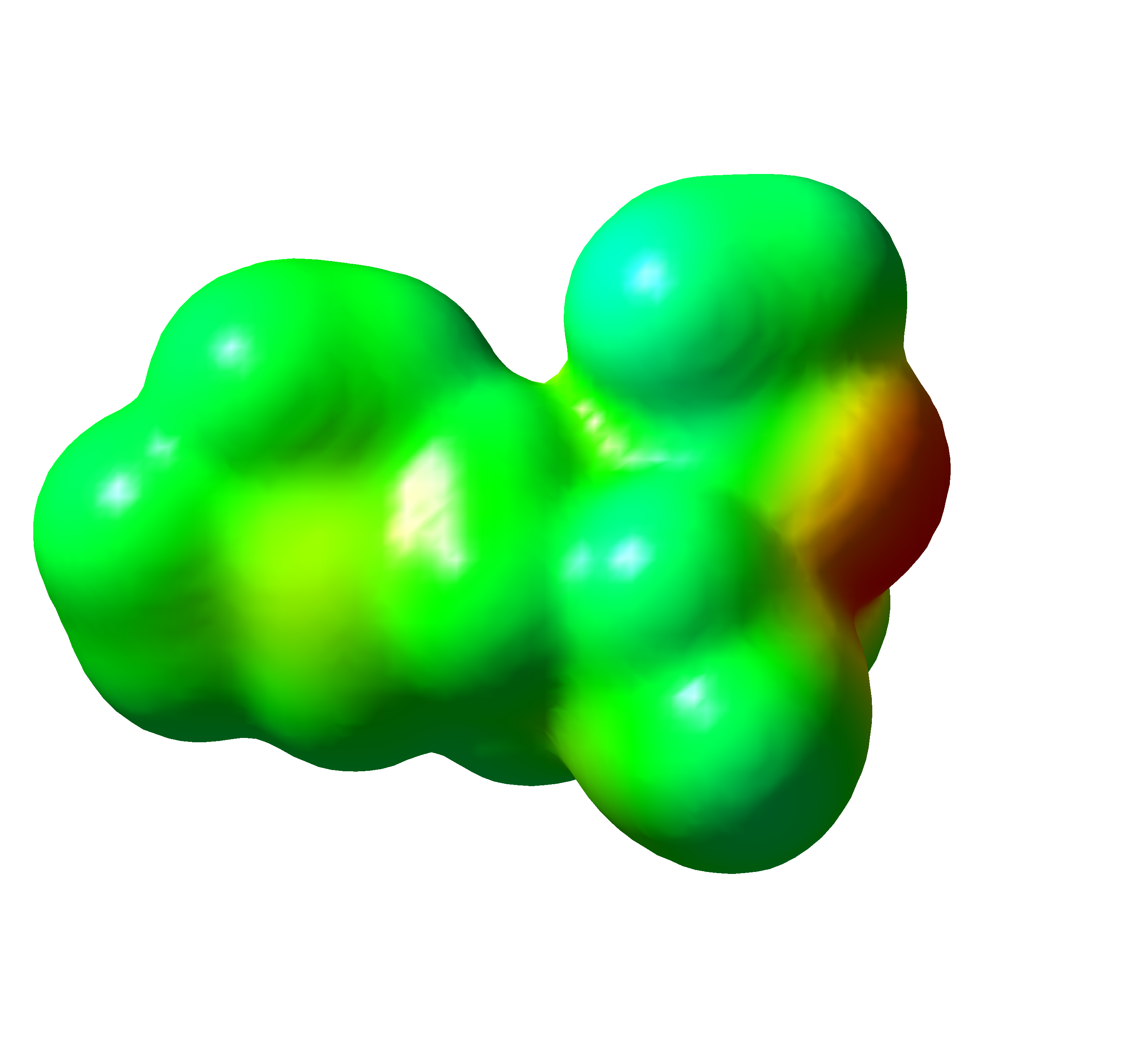

Supplement: S5 Data — (ZIP) [file pone.0343965.s006.zip › PONE-D-25-51583/Vitex Raw material/DFT Vitex all data/comp2/comp2.tif]

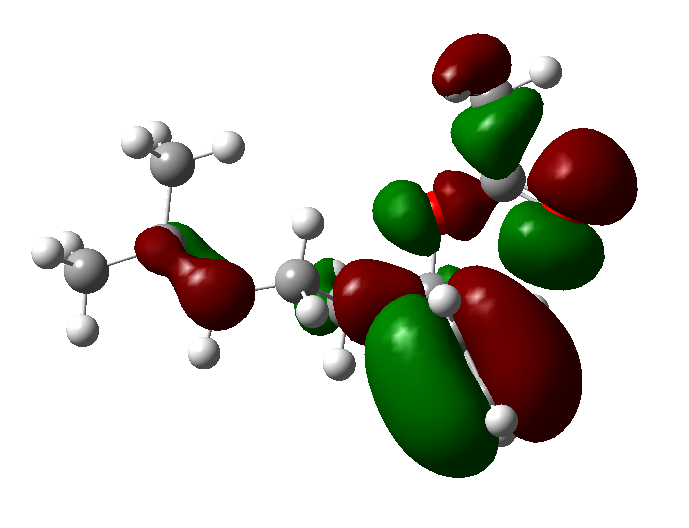

Supplement: S5 Data — (ZIP) [file pone.0343965.s006.zip › PONE-D-25-51583/Vitex Raw material/DFT Vitex all data/comp2/homo-1.tif]

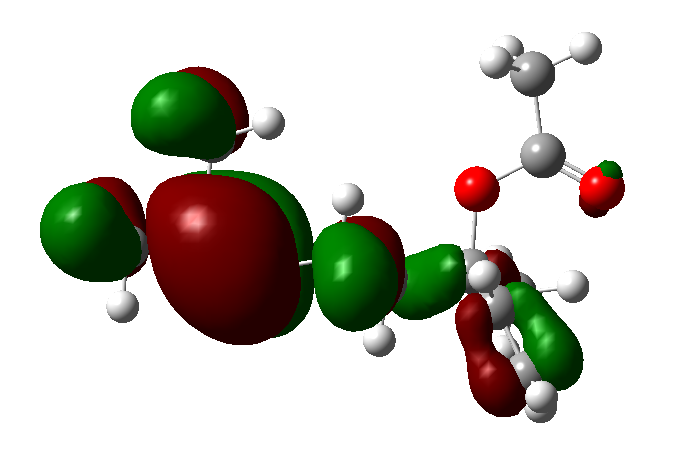

Supplement: S5 Data — (ZIP) [file pone.0343965.s006.zip › PONE-D-25-51583/Vitex Raw material/DFT Vitex all data/comp2/homo.tif]

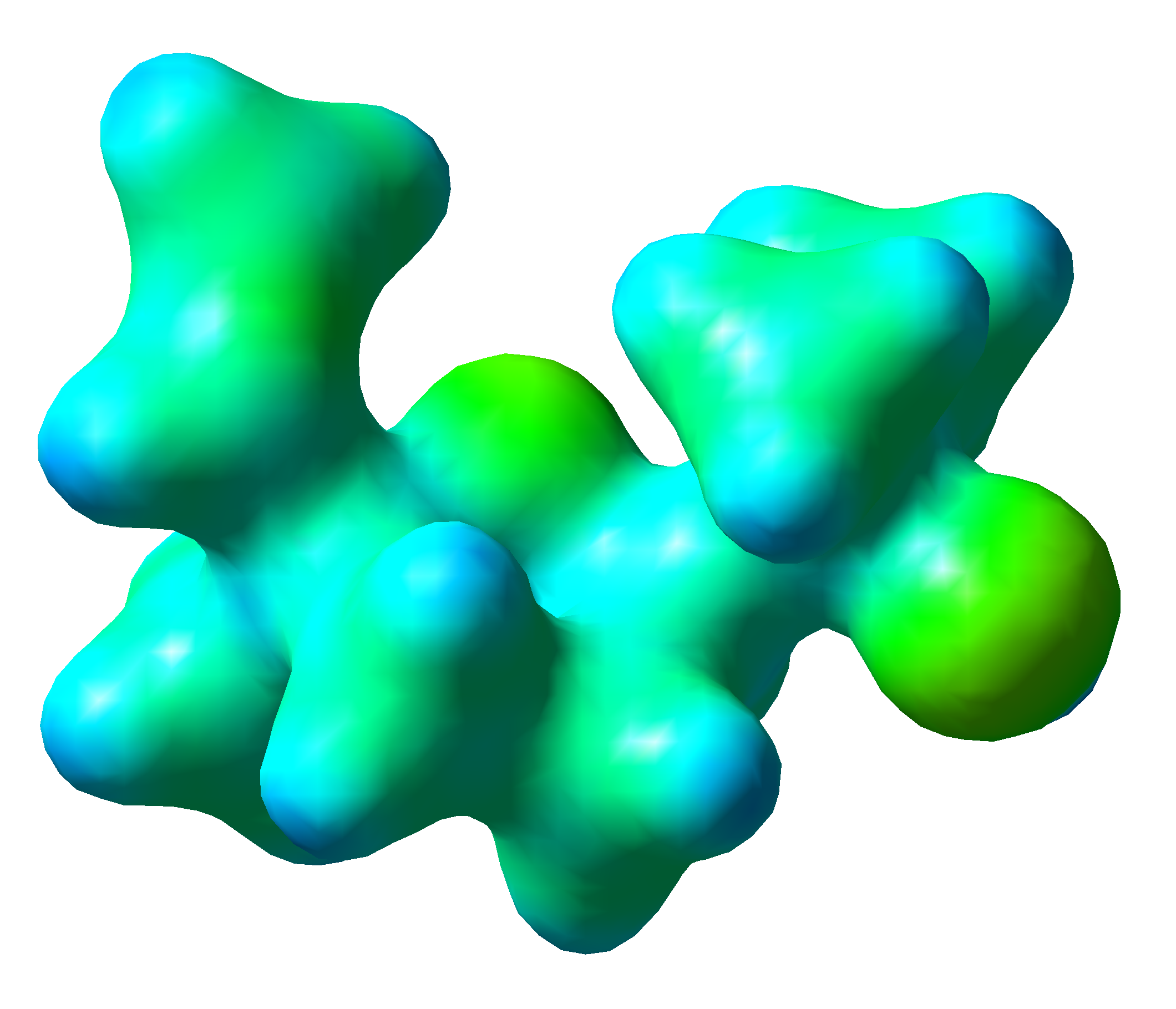

Supplement: S5 Data — (ZIP) [file pone.0343965.s006.zip › PONE-D-25-51583/Vitex Raw material/DFT Vitex all data/comp3/c3.tif]

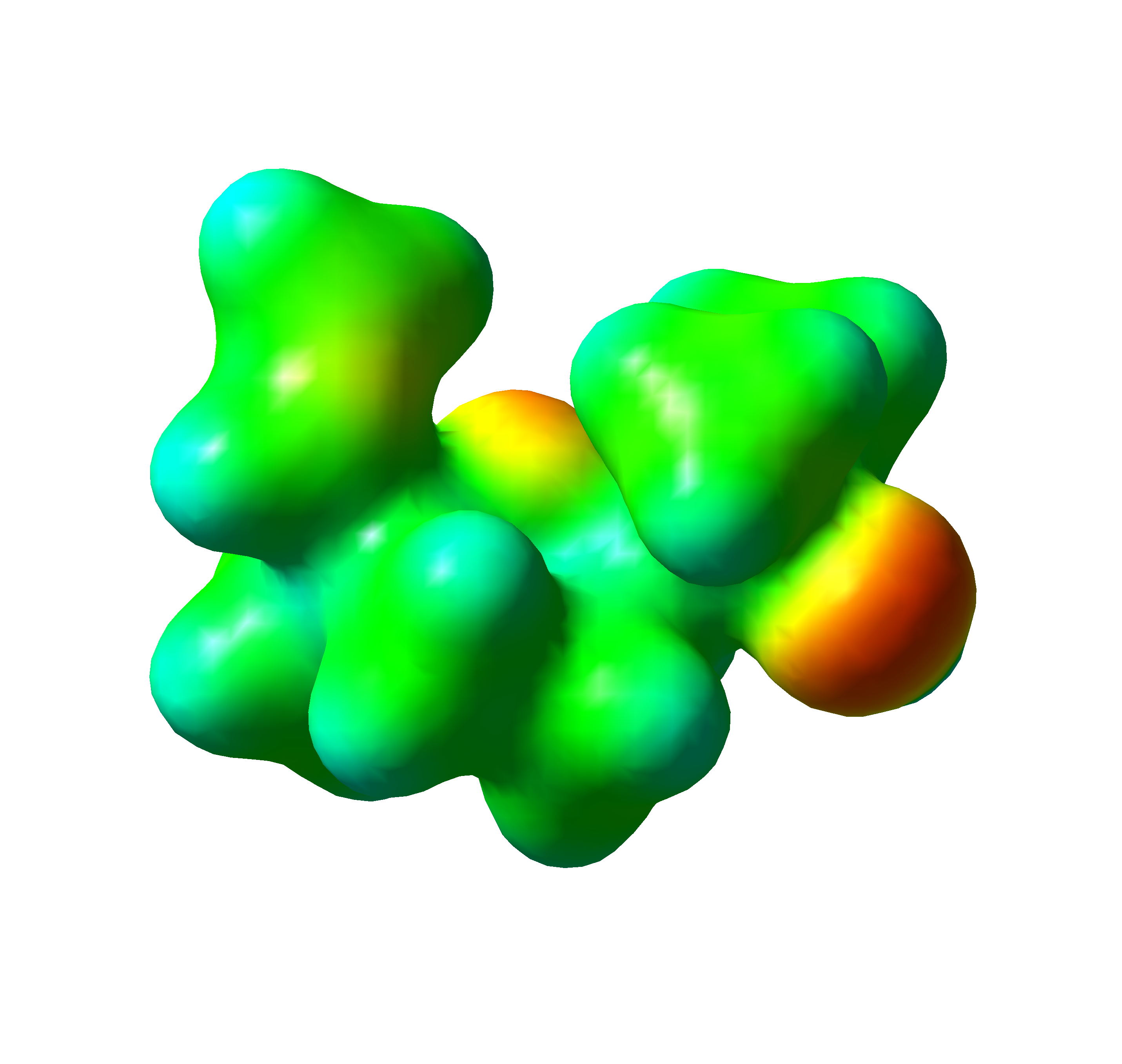

Supplement: S5 Data — (ZIP) [file pone.0343965.s006.zip › PONE-D-25-51583/Vitex Raw material/DFT Vitex all data/comp3/cc3.tif]

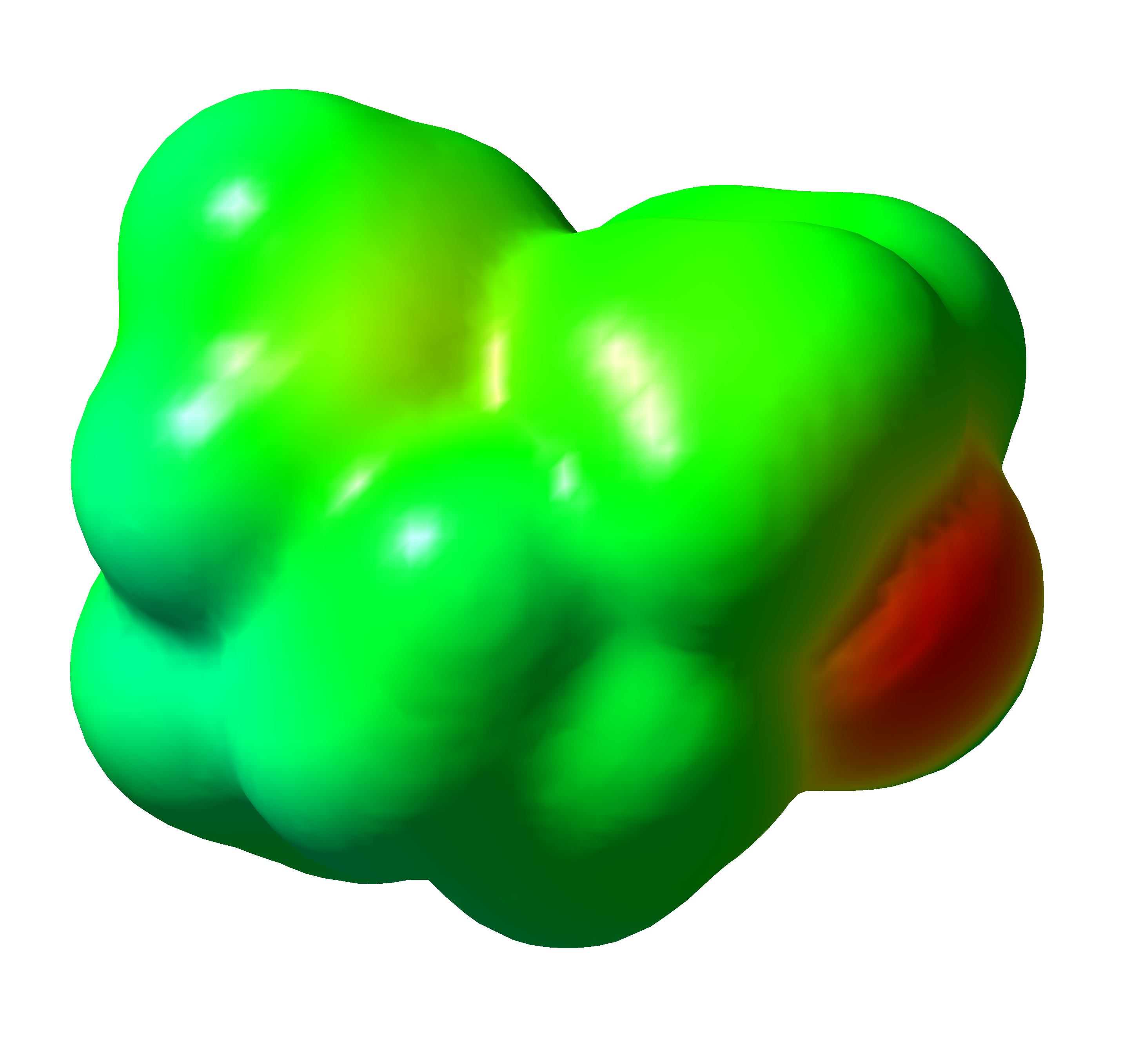

Supplement: S5 Data — (ZIP) [file pone.0343965.s006.zip › PONE-D-25-51583/Vitex Raw material/DFT Vitex all data/comp3/comp3.tif]

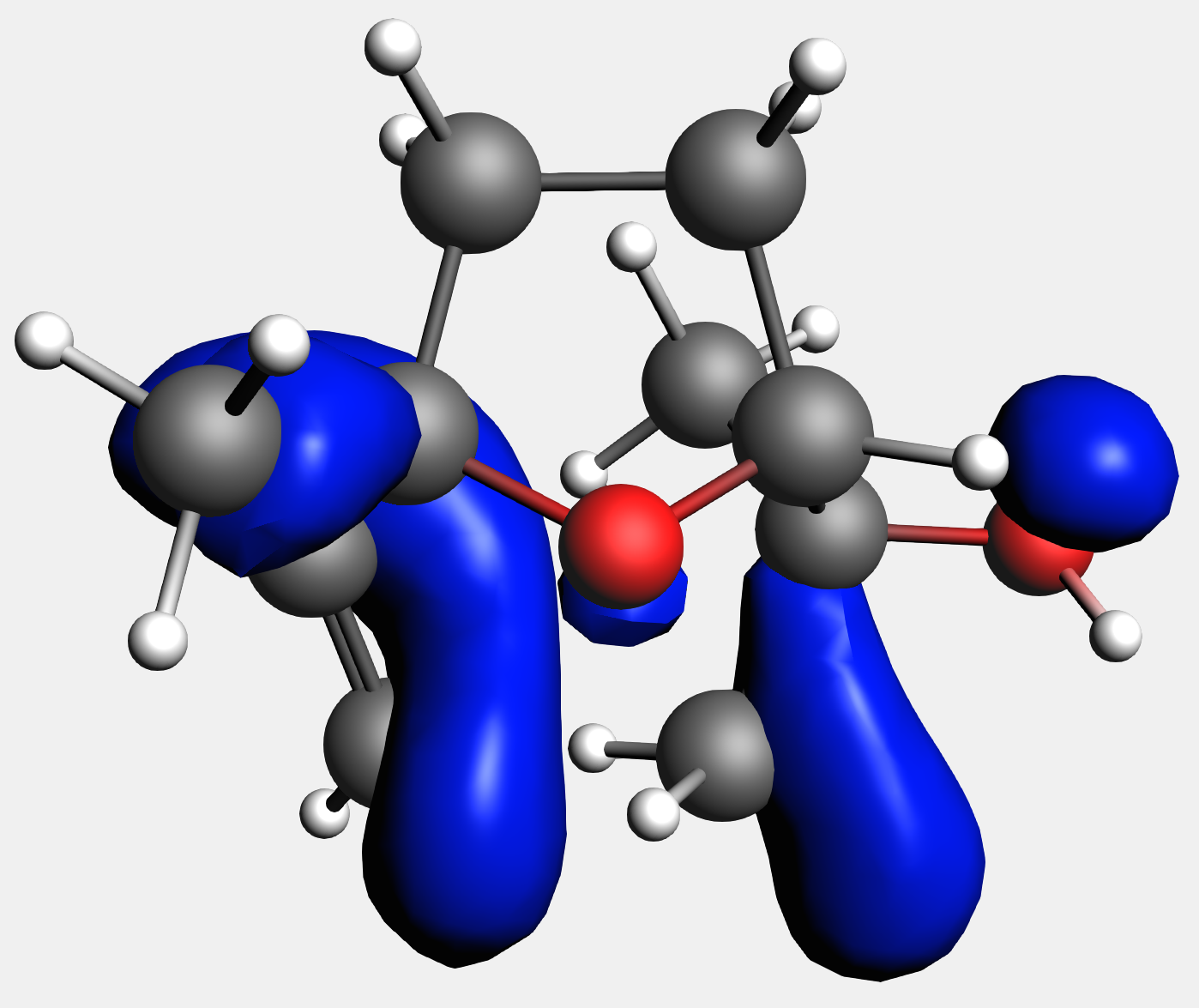

Supplement: S5 Data — (ZIP) [file pone.0343965.s006.zip › PONE-D-25-51583/Vitex Raw material/DFT Vitex all data/comp3/H.png]

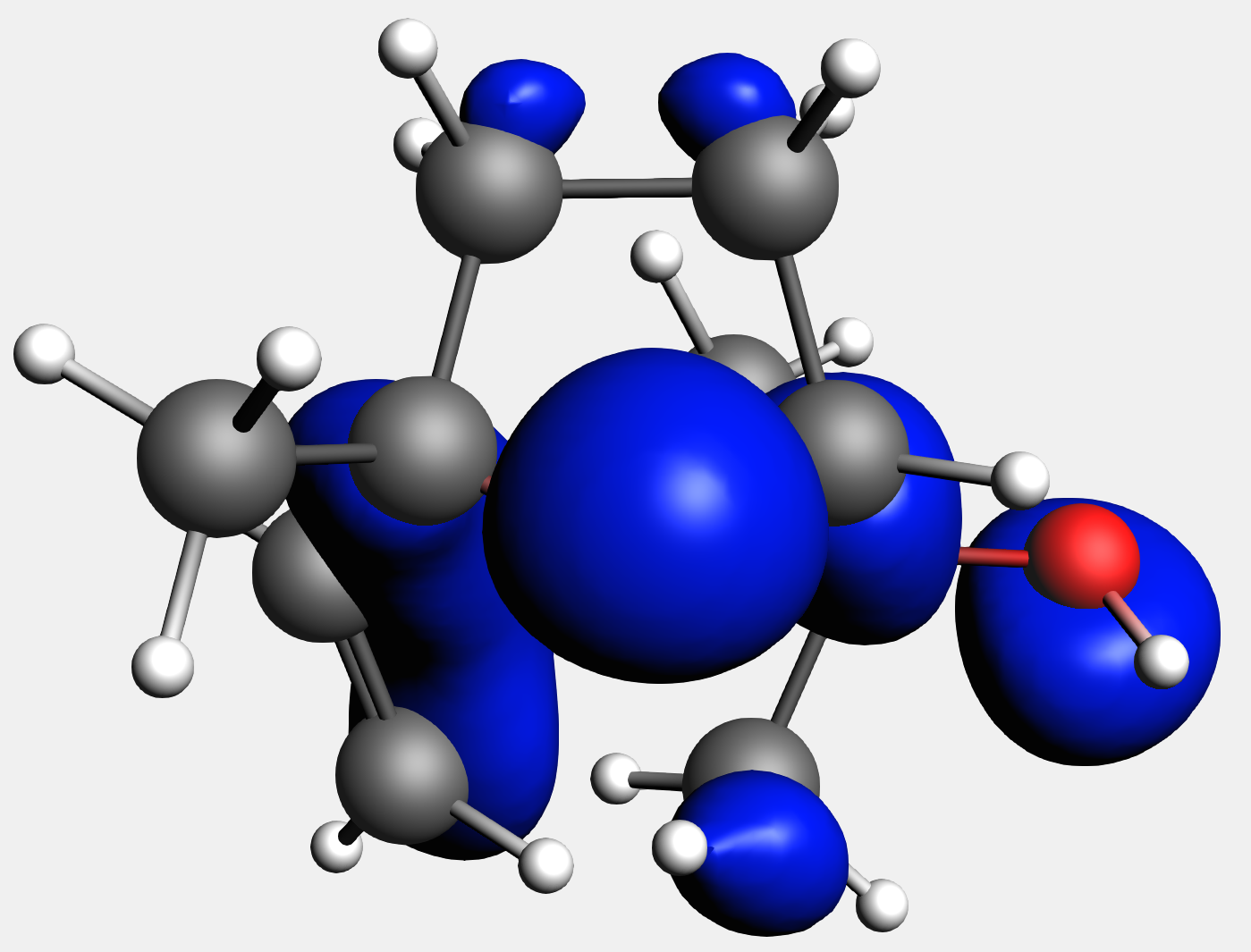

Supplement: S5 Data — (ZIP) [file pone.0343965.s006.zip › PONE-D-25-51583/Vitex Raw material/DFT Vitex all data/comp3/H1.png]

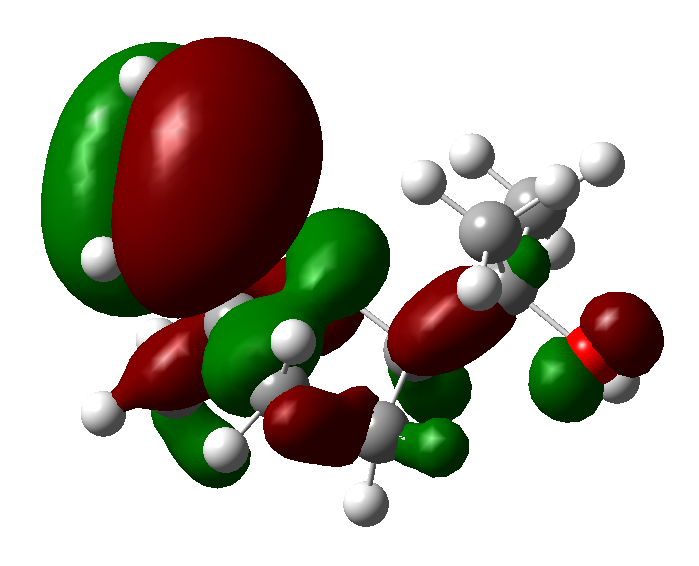

Supplement: S5 Data — (ZIP) [file pone.0343965.s006.zip › PONE-D-25-51583/Vitex Raw material/DFT Vitex all data/comp3/homo-1.tif]

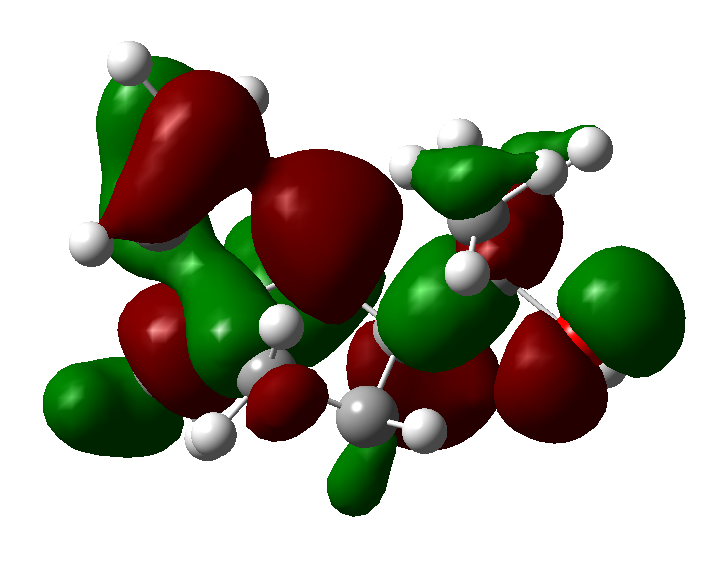

Supplement: S5 Data — (ZIP) [file pone.0343965.s006.zip › PONE-D-25-51583/Vitex Raw material/DFT Vitex all data/comp3/homo.tif]

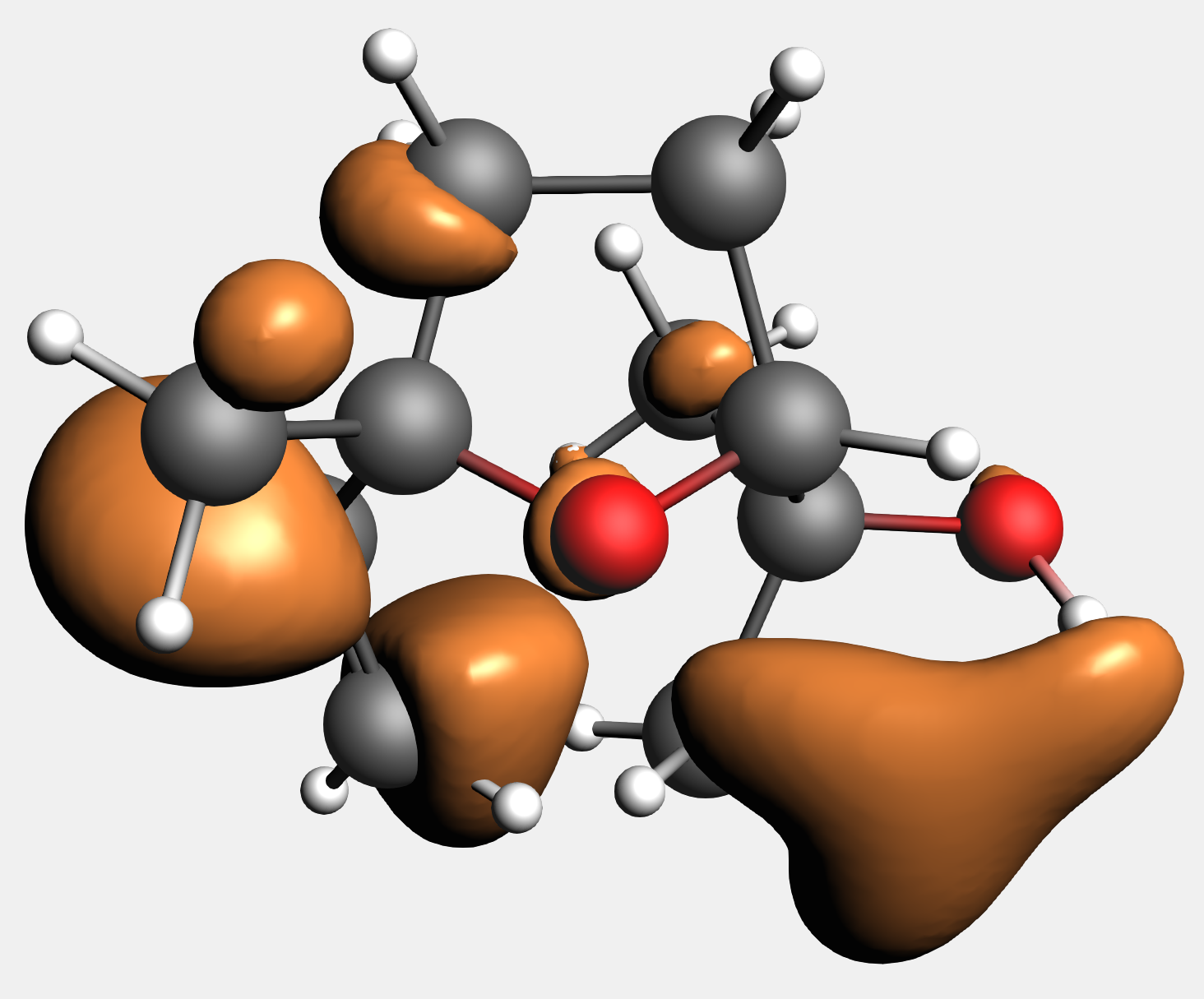

Supplement: S5 Data — (ZIP) [file pone.0343965.s006.zip › PONE-D-25-51583/Vitex Raw material/DFT Vitex all data/comp3/L.png]

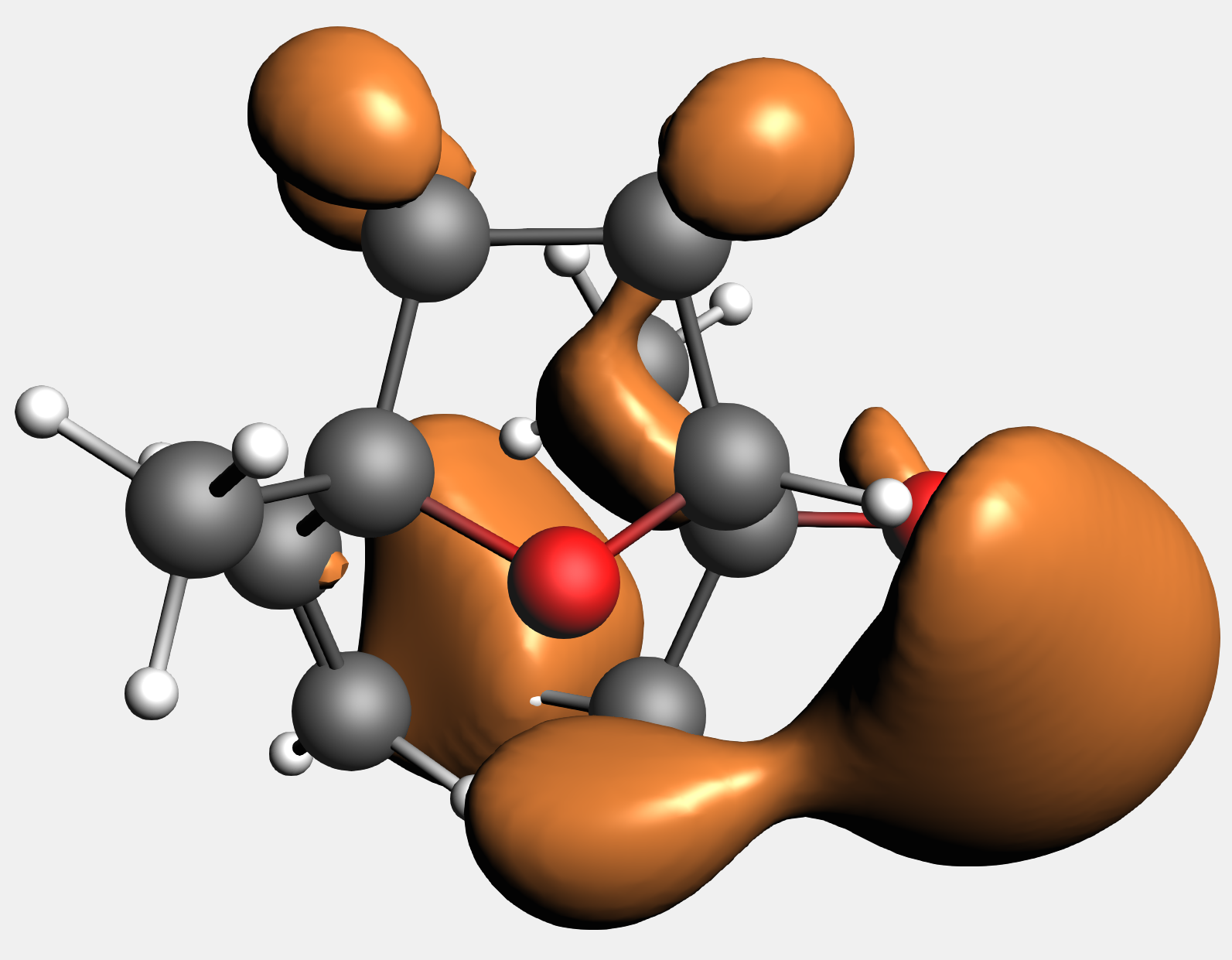

Supplement: S5 Data — (ZIP) [file pone.0343965.s006.zip › PONE-D-25-51583/Vitex Raw material/DFT Vitex all data/comp3/L1.png]

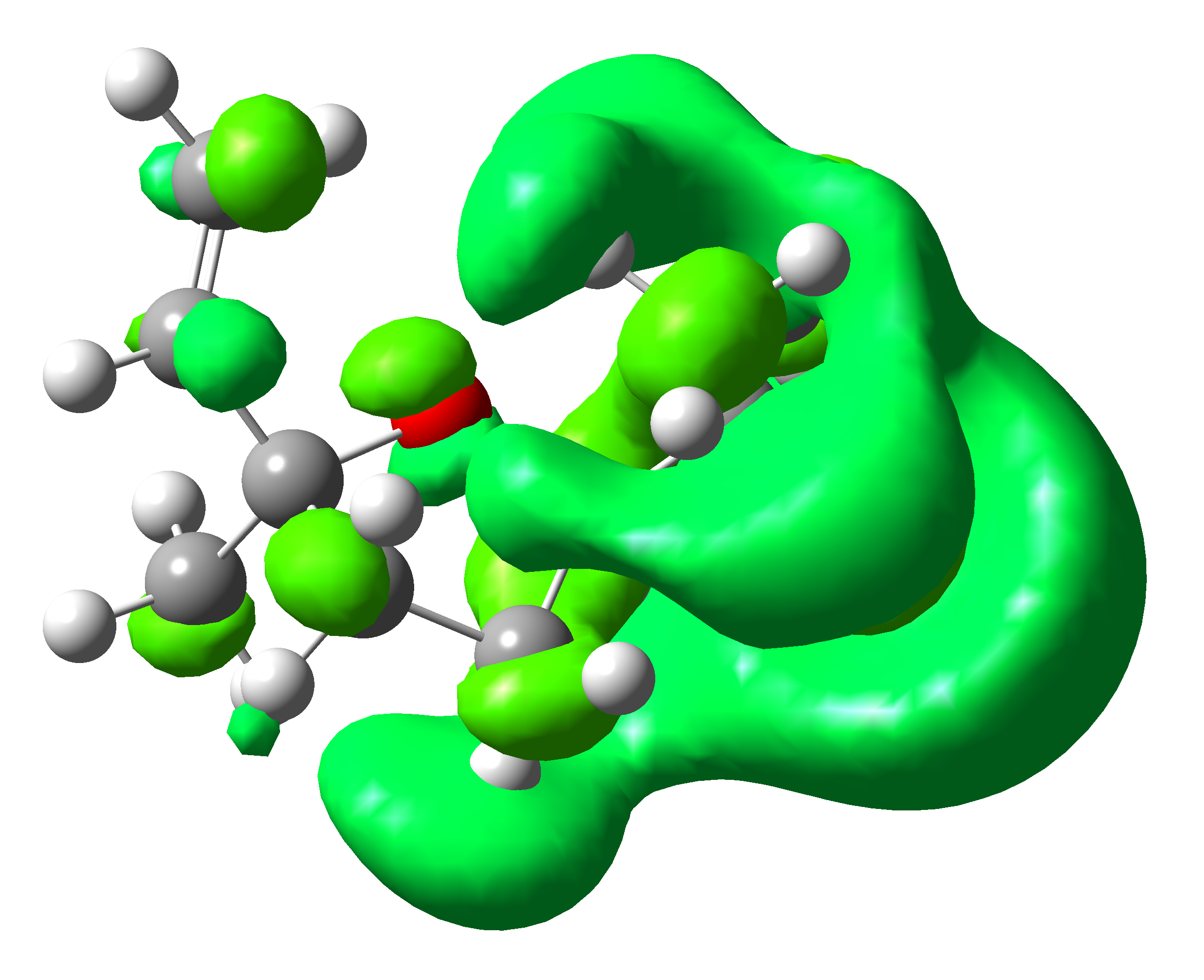

Supplement: S5 Data — (ZIP) [file pone.0343965.s006.zip › PONE-D-25-51583/Vitex Raw material/DFT Vitex all data/comp3/lomo+1.tif]

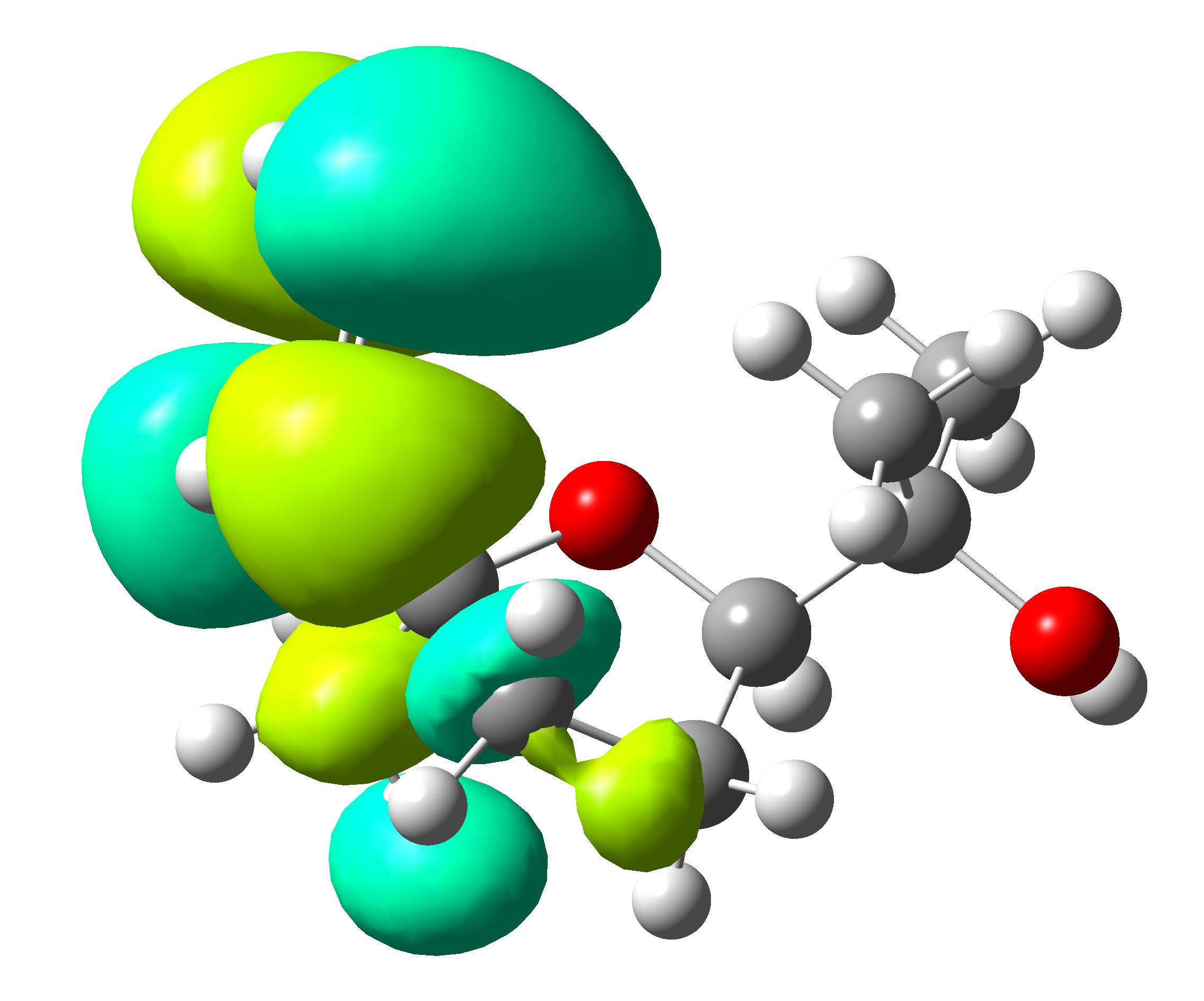

Supplement: S5 Data — (ZIP) [file pone.0343965.s006.zip › PONE-D-25-51583/Vitex Raw material/DFT Vitex all data/comp3/lomo.tif]

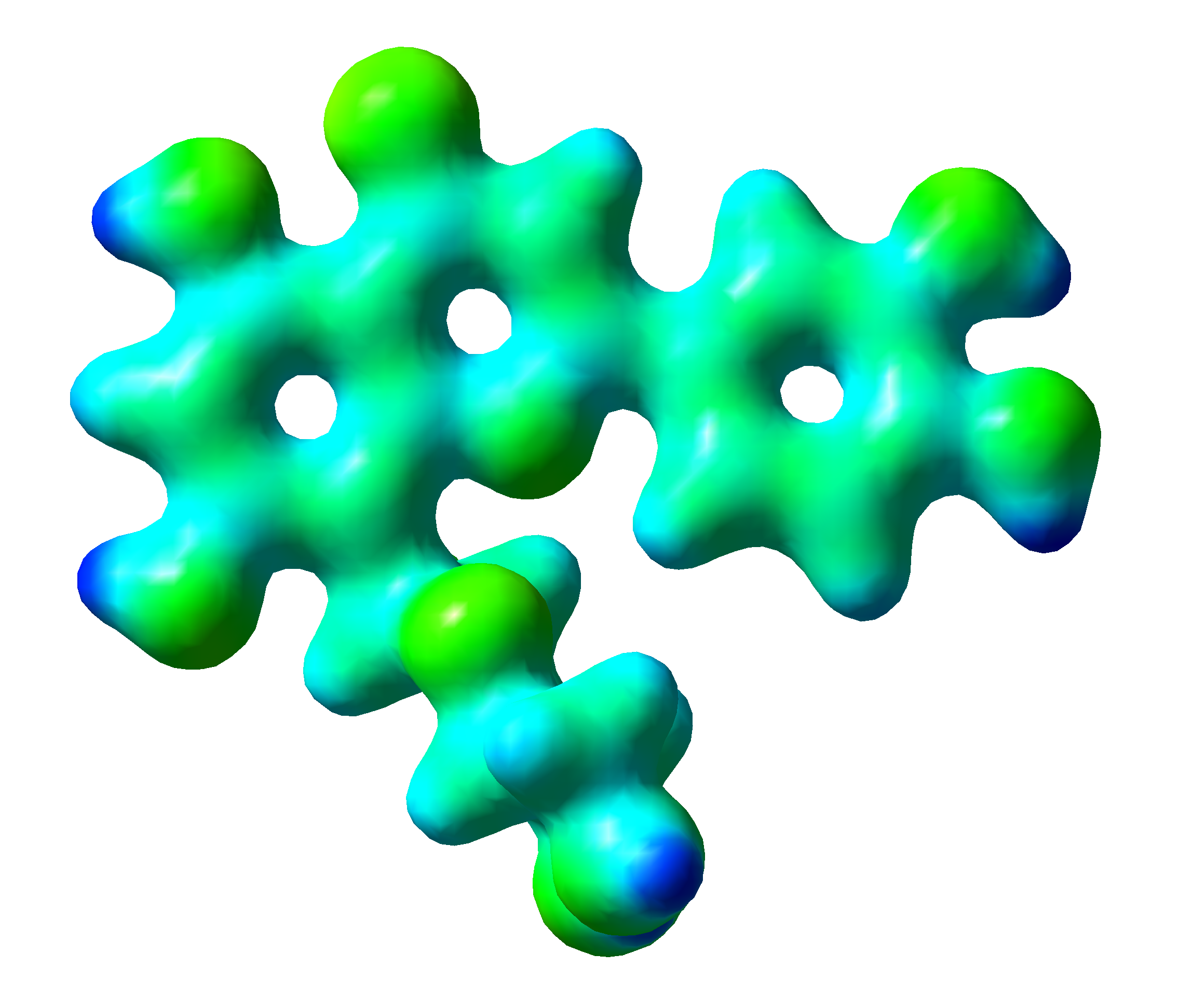

Supplement: S6 Data — (ZIP) [file pone.0343965.s007.zip › PONE-D-25-51583/Vitex Raw material/DFT Vitex all data/comp17/c17.tif]

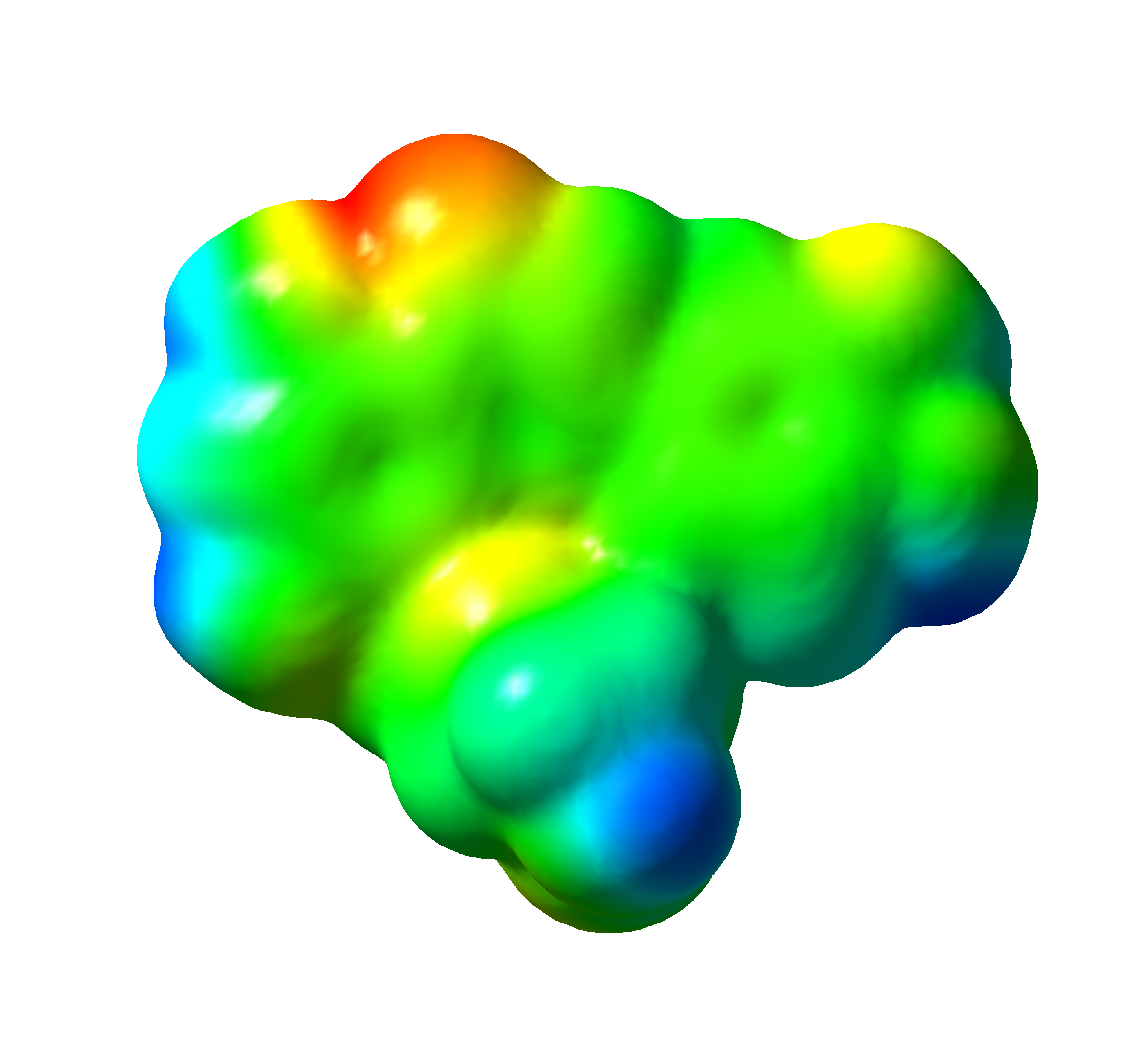

Supplement: S6 Data — (ZIP) [file pone.0343965.s007.zip › PONE-D-25-51583/Vitex Raw material/DFT Vitex all data/comp17/comp17.tif]

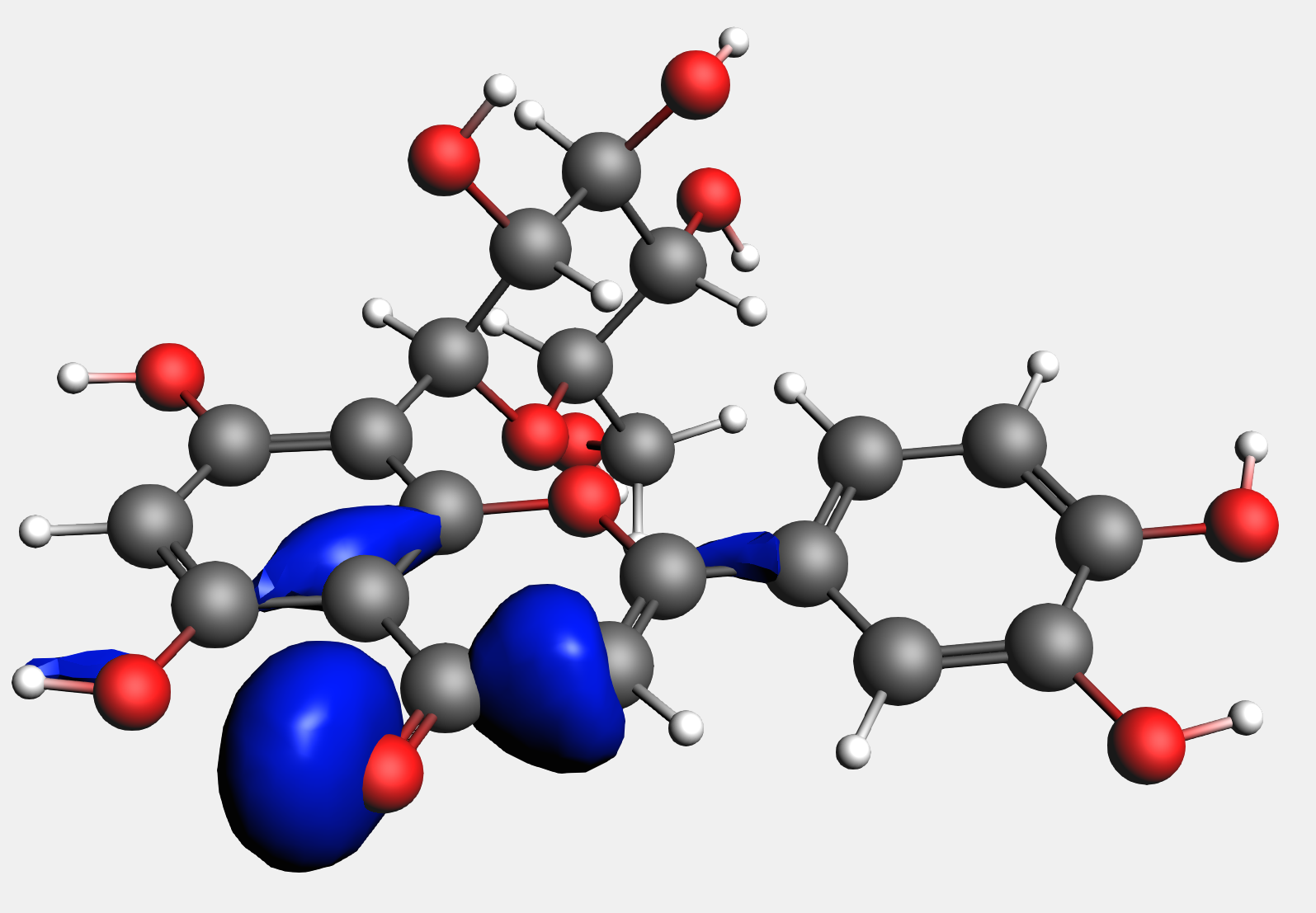

Supplement: S6 Data — (ZIP) [file pone.0343965.s007.zip › PONE-D-25-51583/Vitex Raw material/DFT Vitex all data/comp17/H.png]

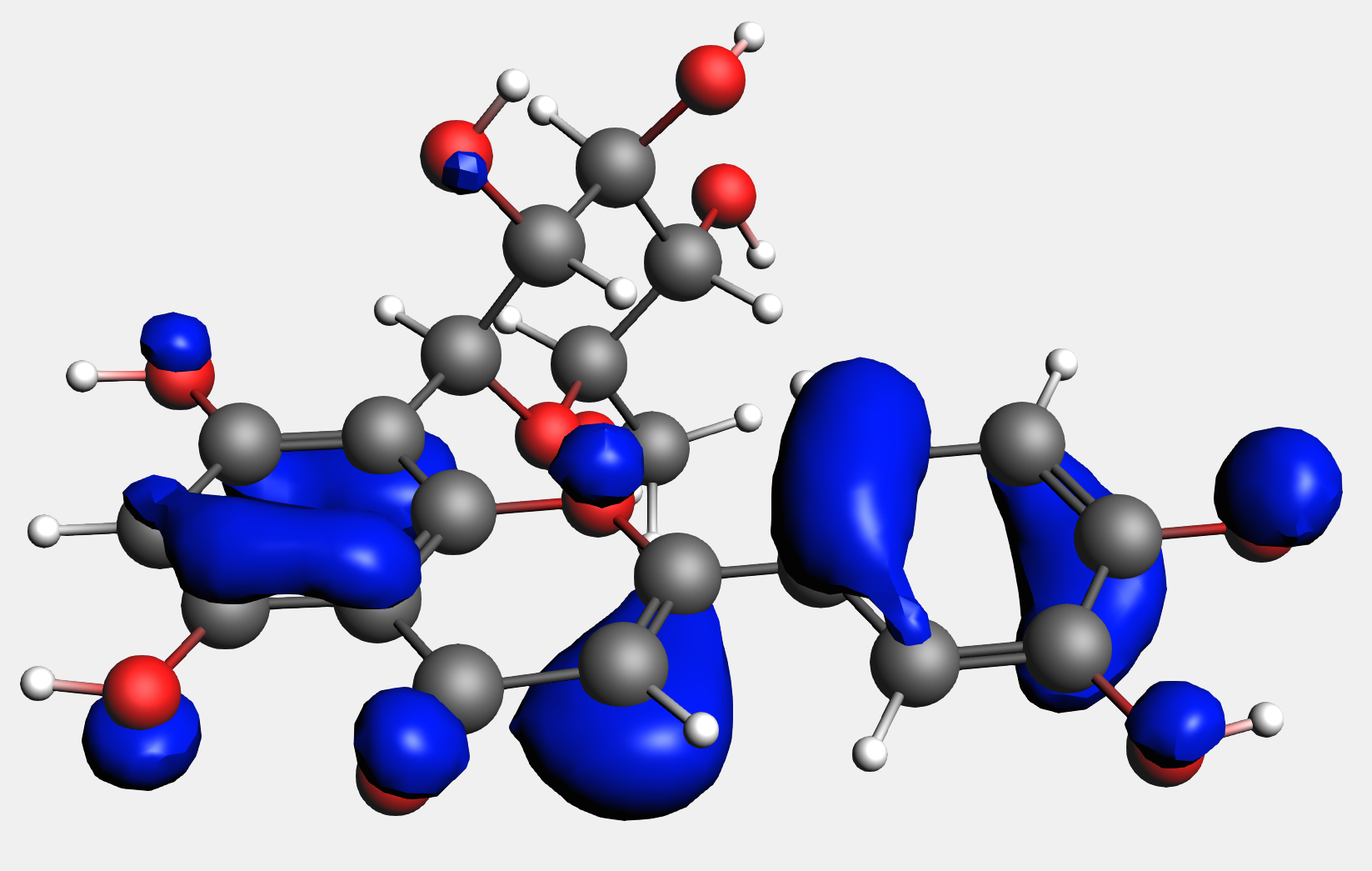

Supplement: S6 Data — (ZIP) [file pone.0343965.s007.zip › PONE-D-25-51583/Vitex Raw material/DFT Vitex all data/comp17/H1.png]

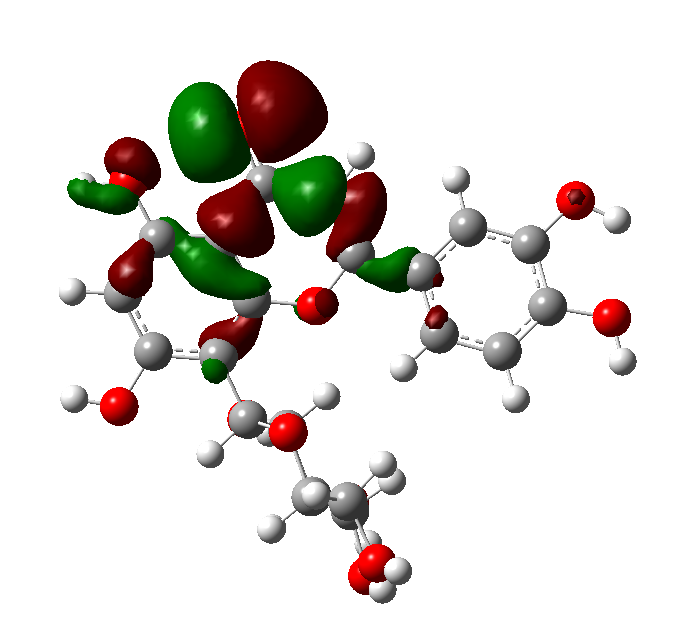

Supplement: S6 Data — (ZIP) [file pone.0343965.s007.zip › PONE-D-25-51583/Vitex Raw material/DFT Vitex all data/comp17/homo-1.tif]

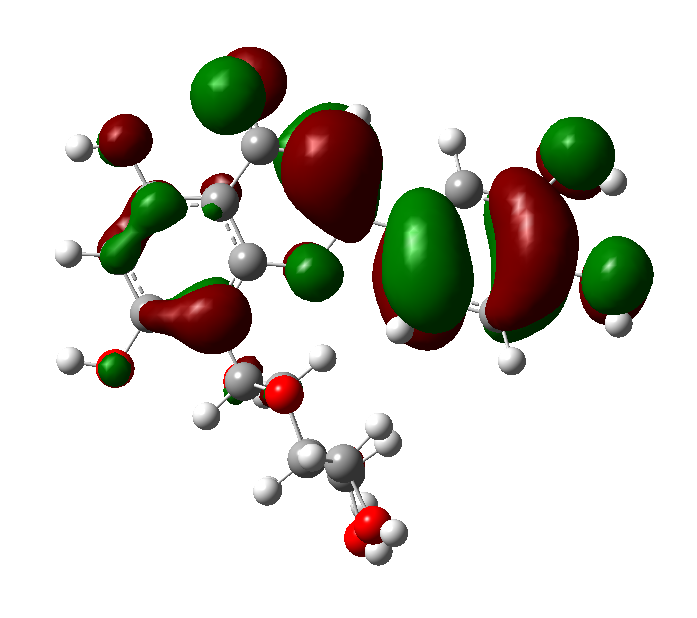

Supplement: S6 Data — (ZIP) [file pone.0343965.s007.zip › PONE-D-25-51583/Vitex Raw material/DFT Vitex all data/comp17/homo.tif]

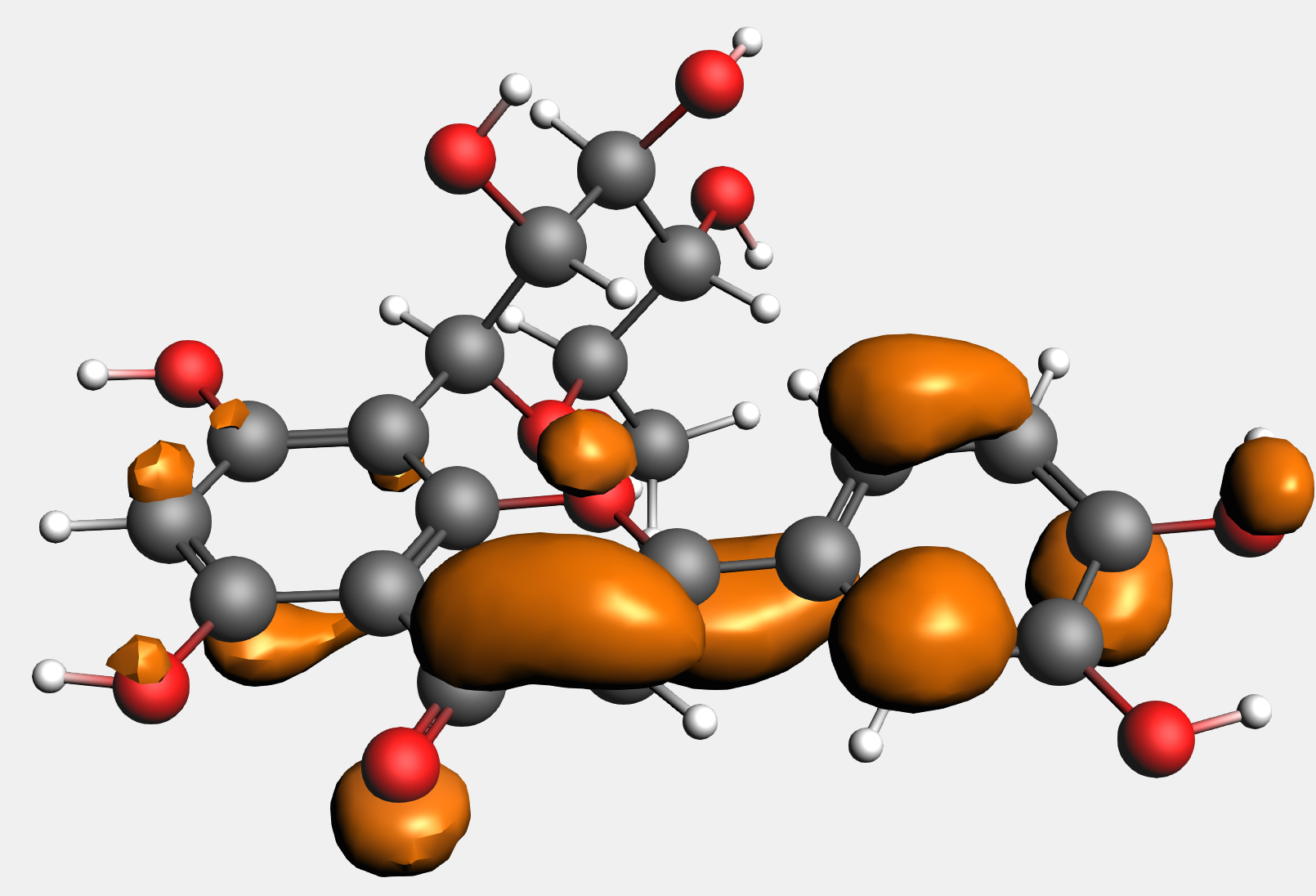

Supplement: S6 Data — (ZIP) [file pone.0343965.s007.zip › PONE-D-25-51583/Vitex Raw material/DFT Vitex all data/comp17/L.png]

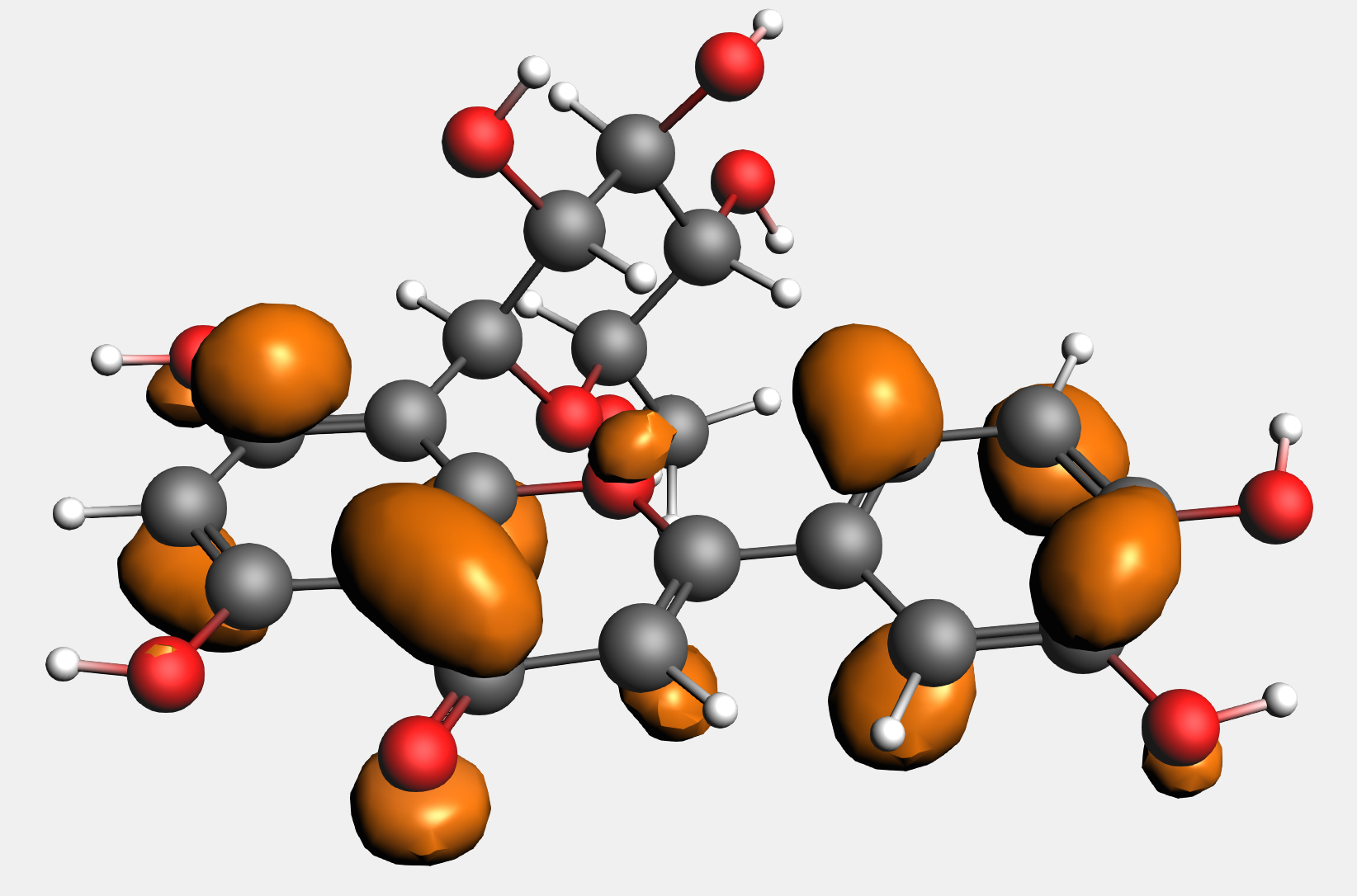

Supplement: S6 Data — (ZIP) [file pone.0343965.s007.zip › PONE-D-25-51583/Vitex Raw material/DFT Vitex all data/comp17/L1.png]

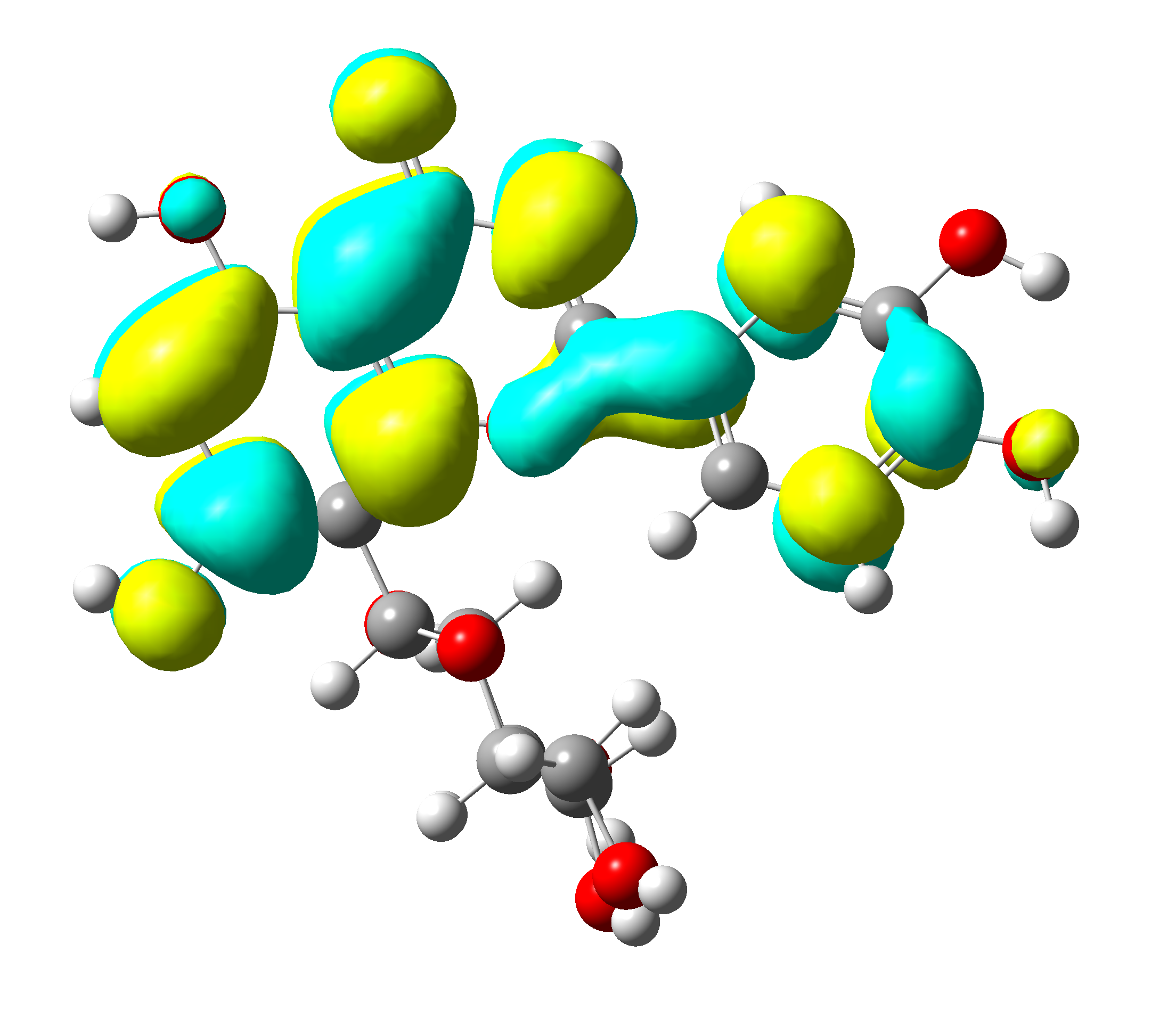

Supplement: S6 Data — (ZIP) [file pone.0343965.s007.zip › PONE-D-25-51583/Vitex Raw material/DFT Vitex all data/comp17/lomo+1.tif]

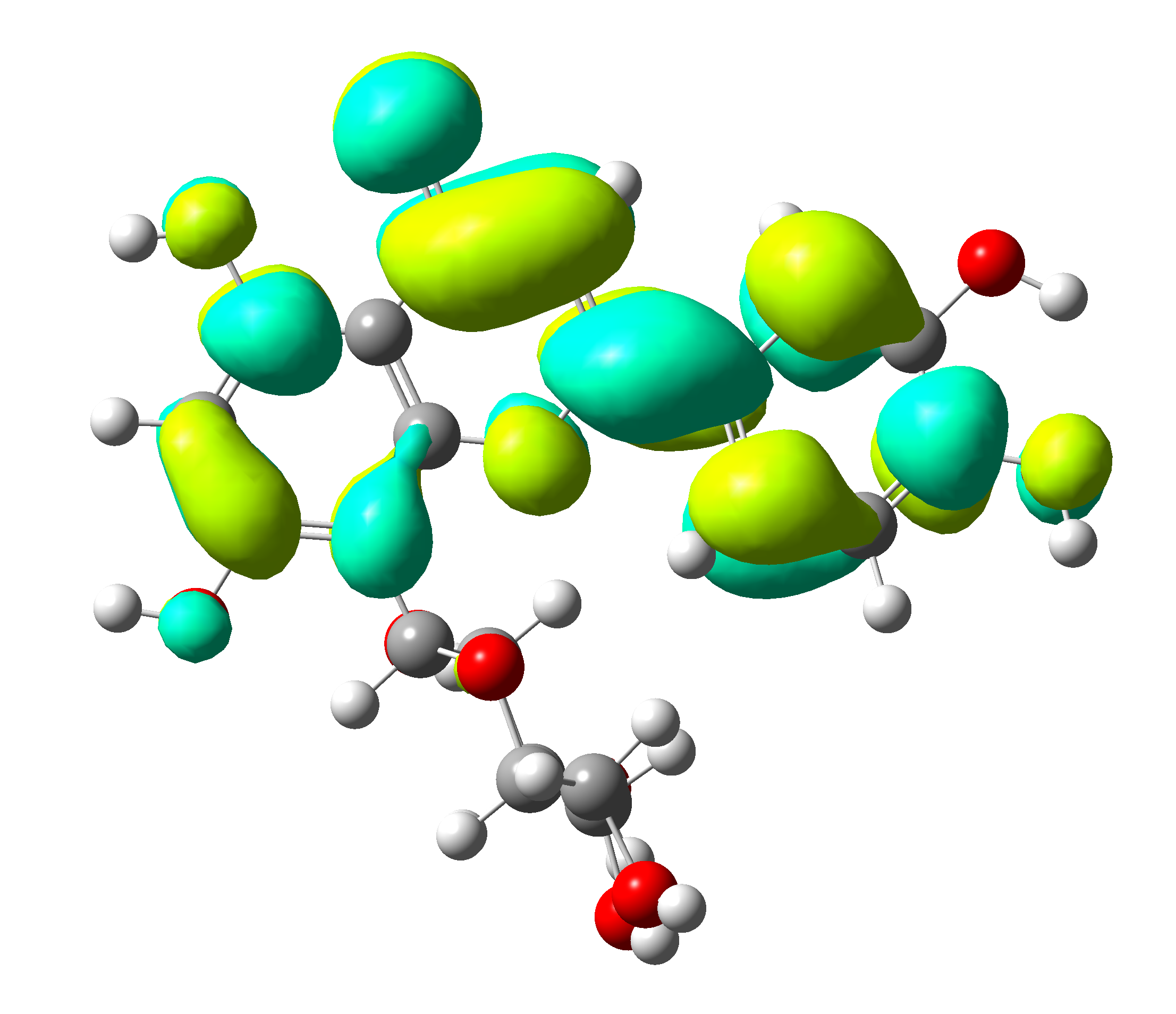

Supplement: S6 Data — (ZIP) [file pone.0343965.s007.zip › PONE-D-25-51583/Vitex Raw material/DFT Vitex all data/comp17/lomo.tif]

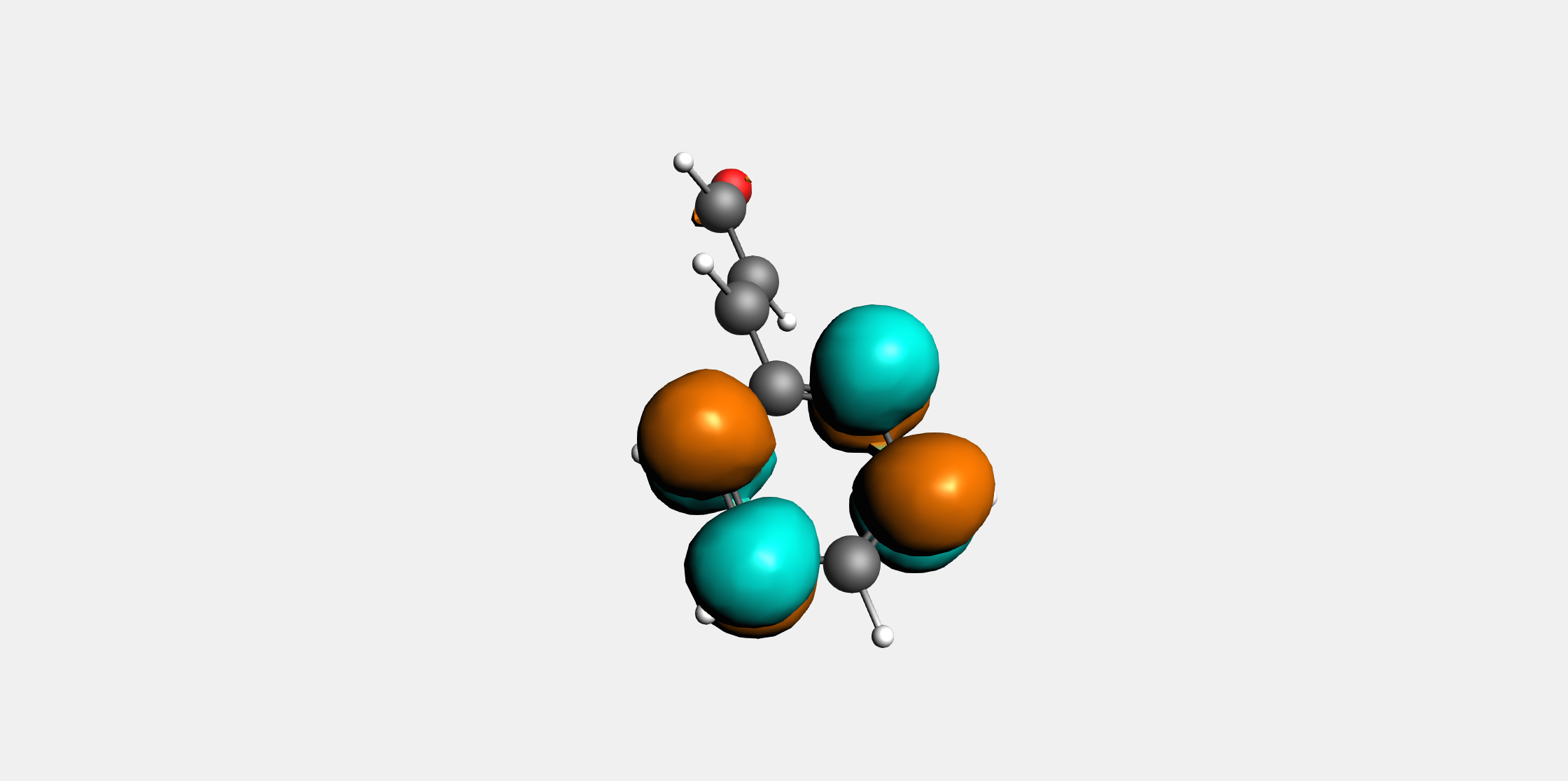

Supplement: S7 Data — (ZIP) [file pone.0343965.s008.zip › PONE-D-25-51583/Vitex Raw material/DFT Vitex all data/comp6/C6.results/L1.png]

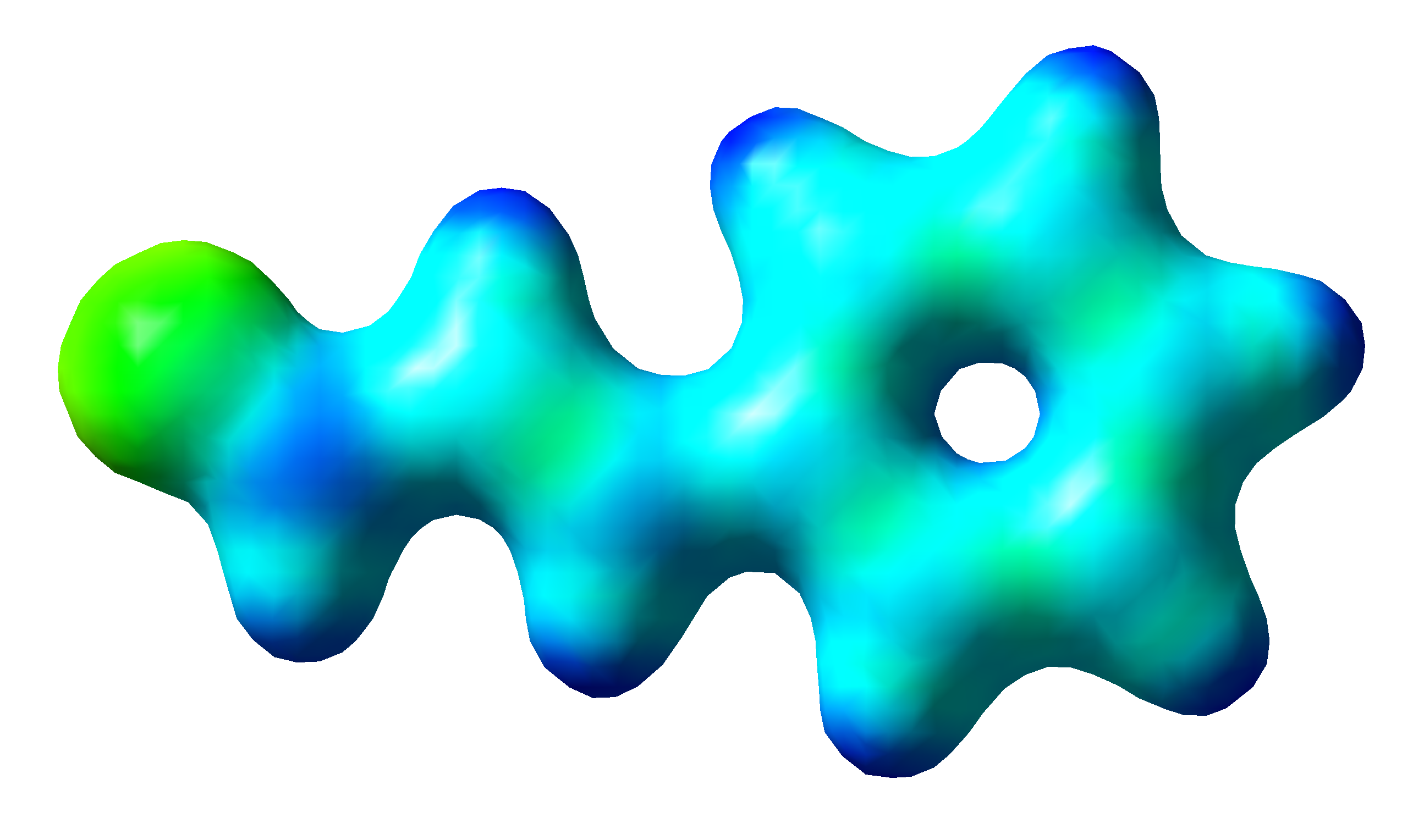

Supplement: S7 Data — (ZIP) [file pone.0343965.s008.zip › PONE-D-25-51583/Vitex Raw material/DFT Vitex all data/comp6/c6.tif]

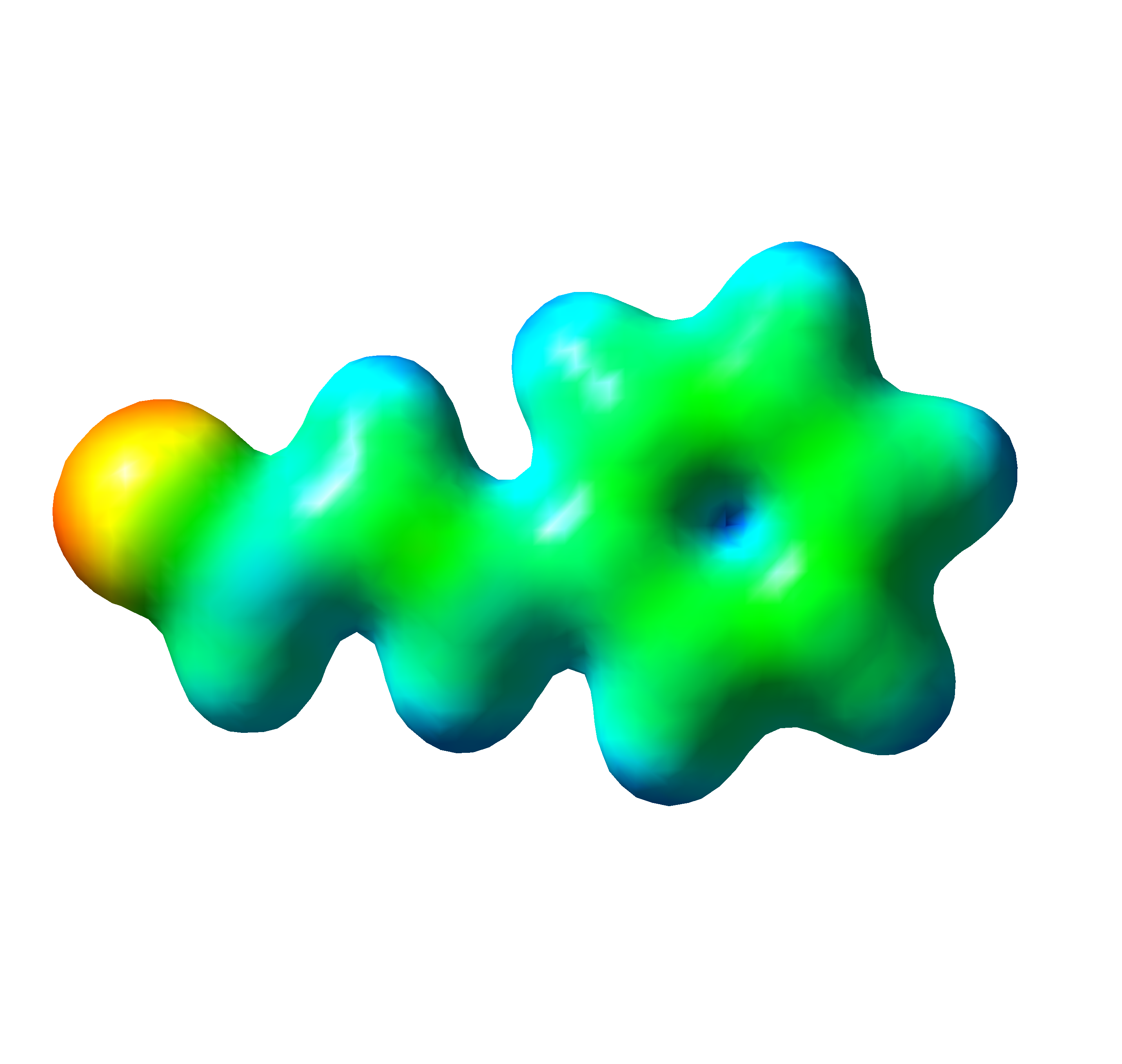

Supplement: S7 Data — (ZIP) [file pone.0343965.s008.zip › PONE-D-25-51583/Vitex Raw material/DFT Vitex all data/comp6/cc6.tif]

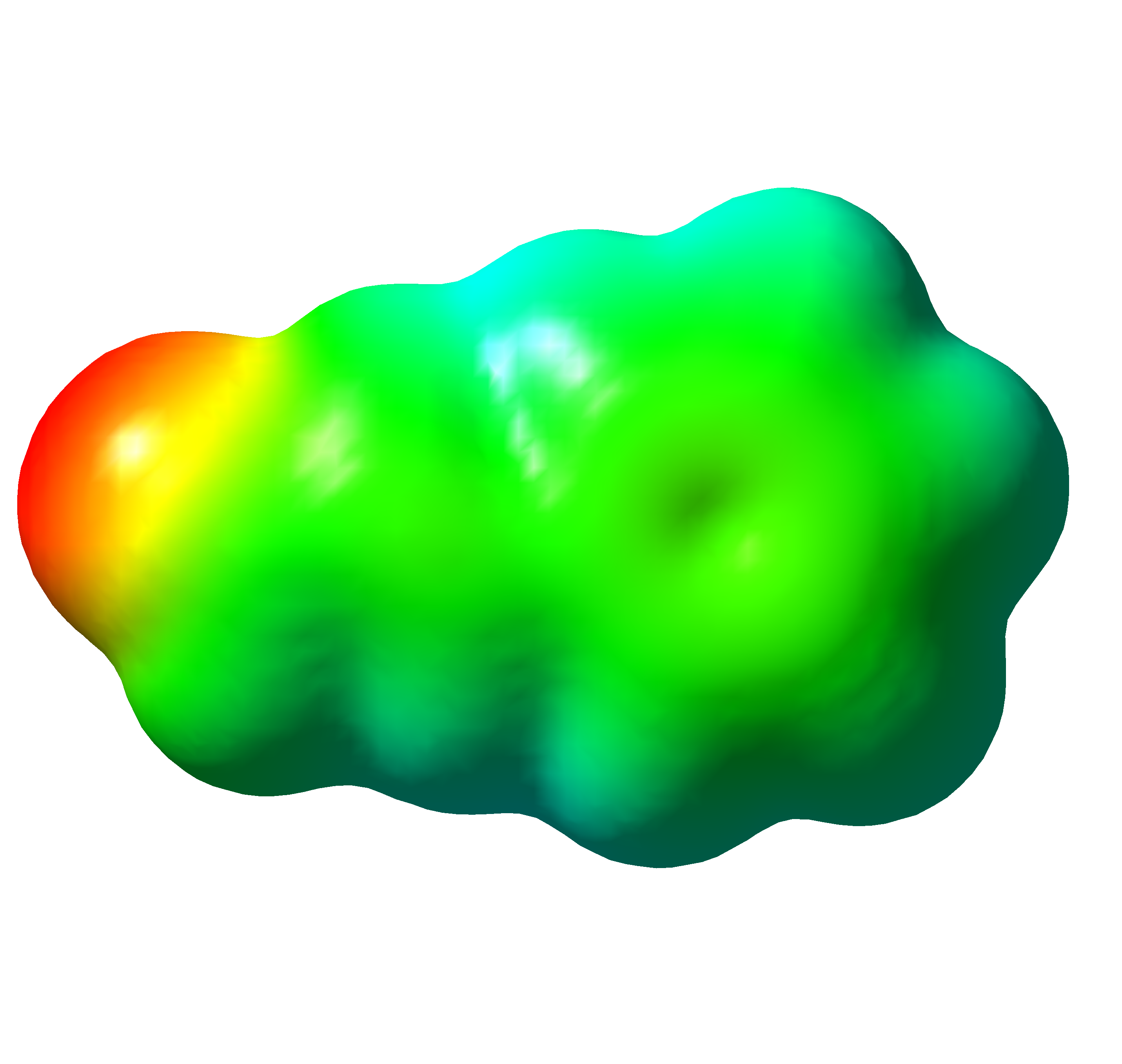

Supplement: S7 Data — (ZIP) [file pone.0343965.s008.zip › PONE-D-25-51583/Vitex Raw material/DFT Vitex all data/comp6/comp6.tif]

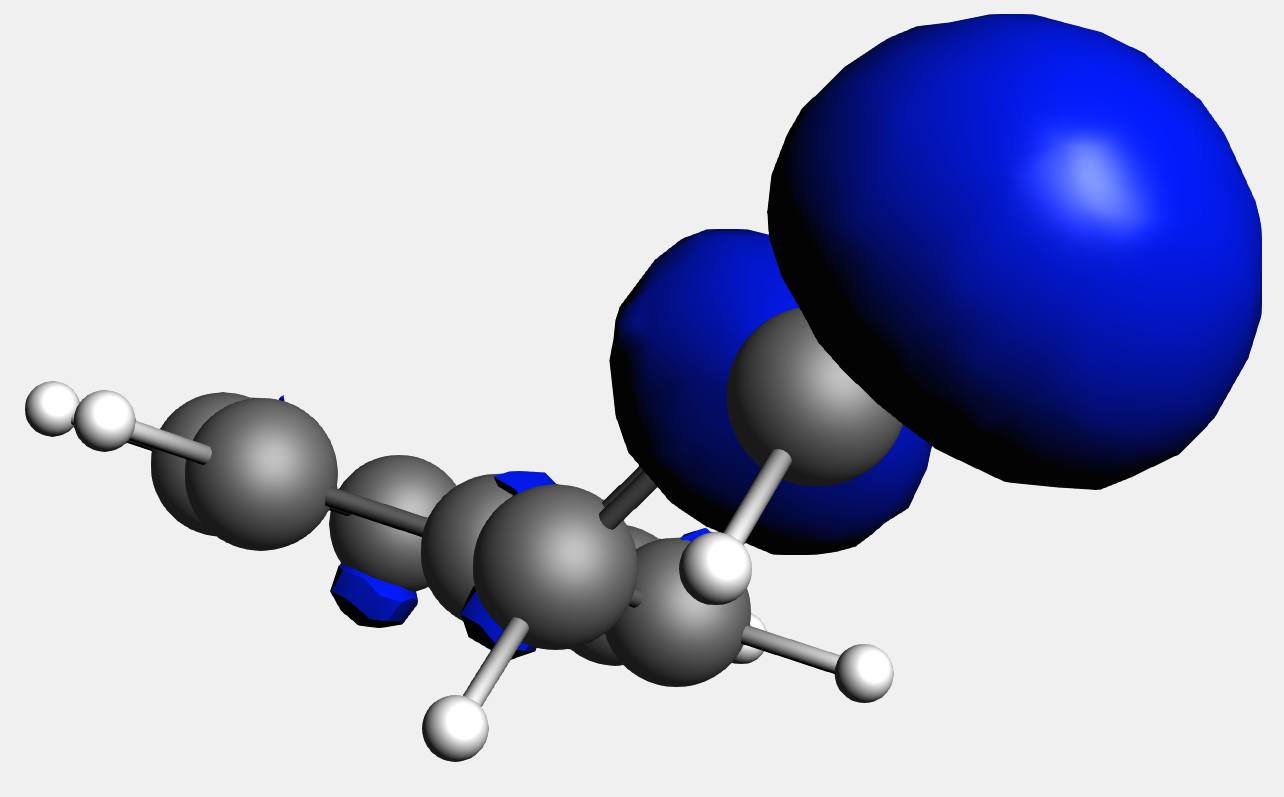

Supplement: S7 Data — (ZIP) [file pone.0343965.s008.zip › PONE-D-25-51583/Vitex Raw material/DFT Vitex all data/comp6/H.png]

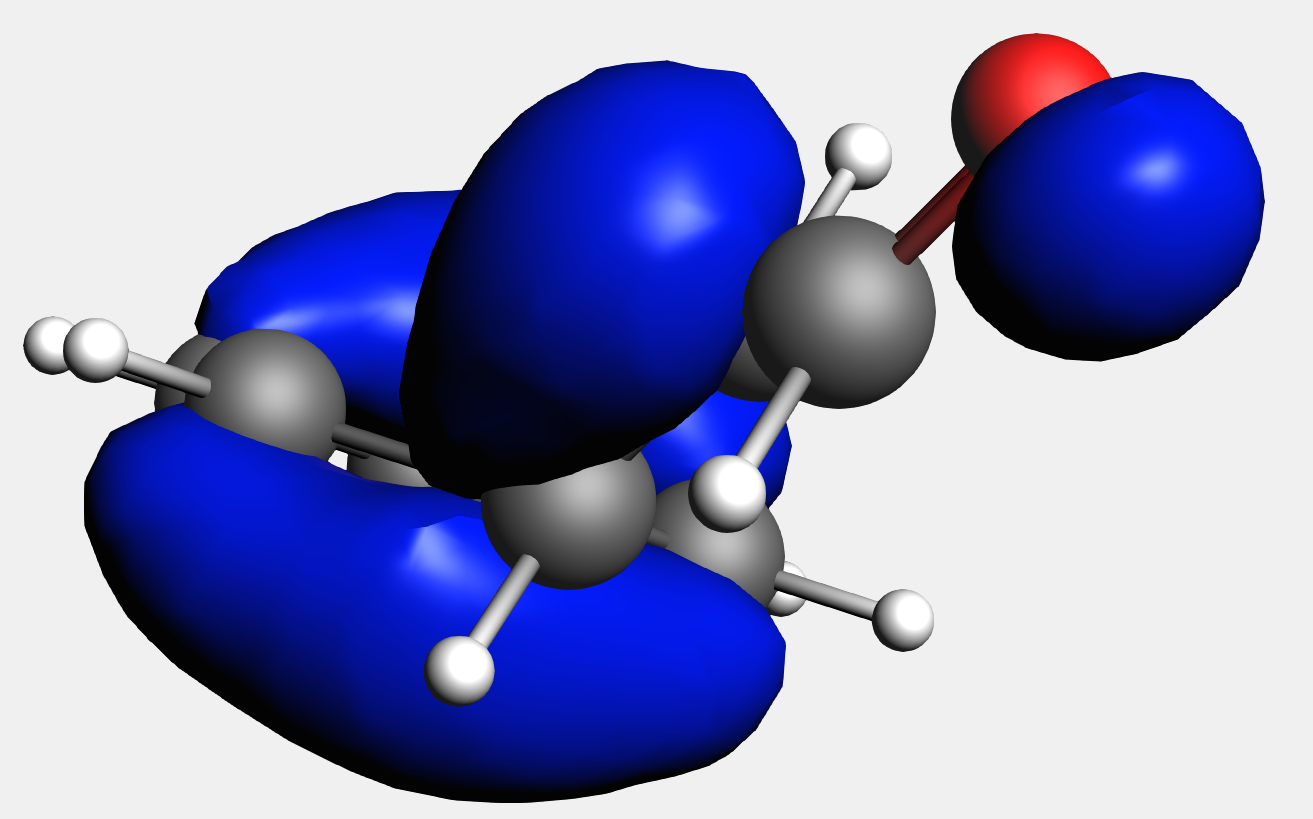

Supplement: S7 Data — (ZIP) [file pone.0343965.s008.zip › PONE-D-25-51583/Vitex Raw material/DFT Vitex all data/comp6/H1.png]

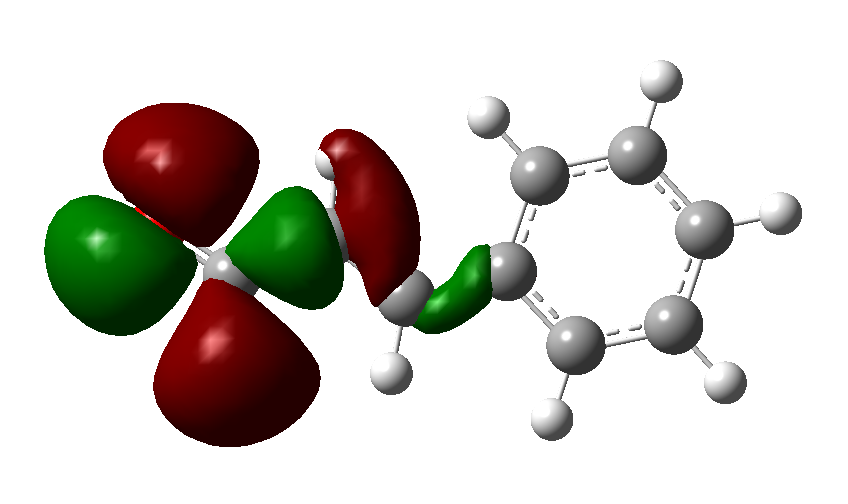

Supplement: S7 Data — (ZIP) [file pone.0343965.s008.zip › PONE-D-25-51583/Vitex Raw material/DFT Vitex all data/comp6/homo-1.tif]

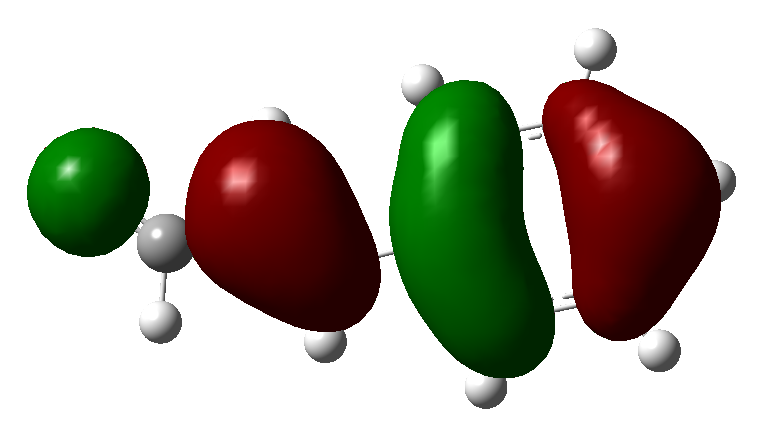

Supplement: S7 Data — (ZIP) [file pone.0343965.s008.zip › PONE-D-25-51583/Vitex Raw material/DFT Vitex all data/comp6/homo.tif]

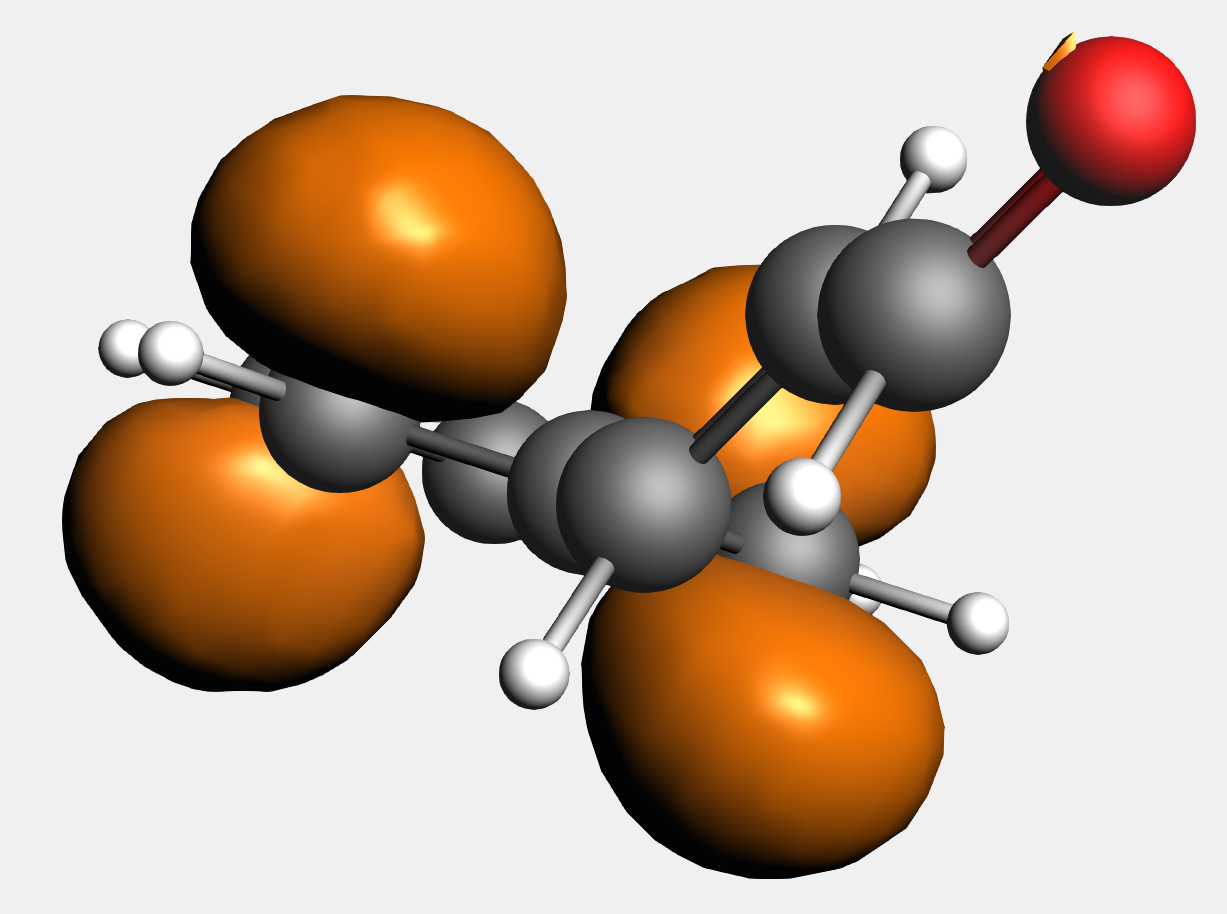

Supplement: S7 Data — (ZIP) [file pone.0343965.s008.zip › PONE-D-25-51583/Vitex Raw material/DFT Vitex all data/comp6/L11.png]

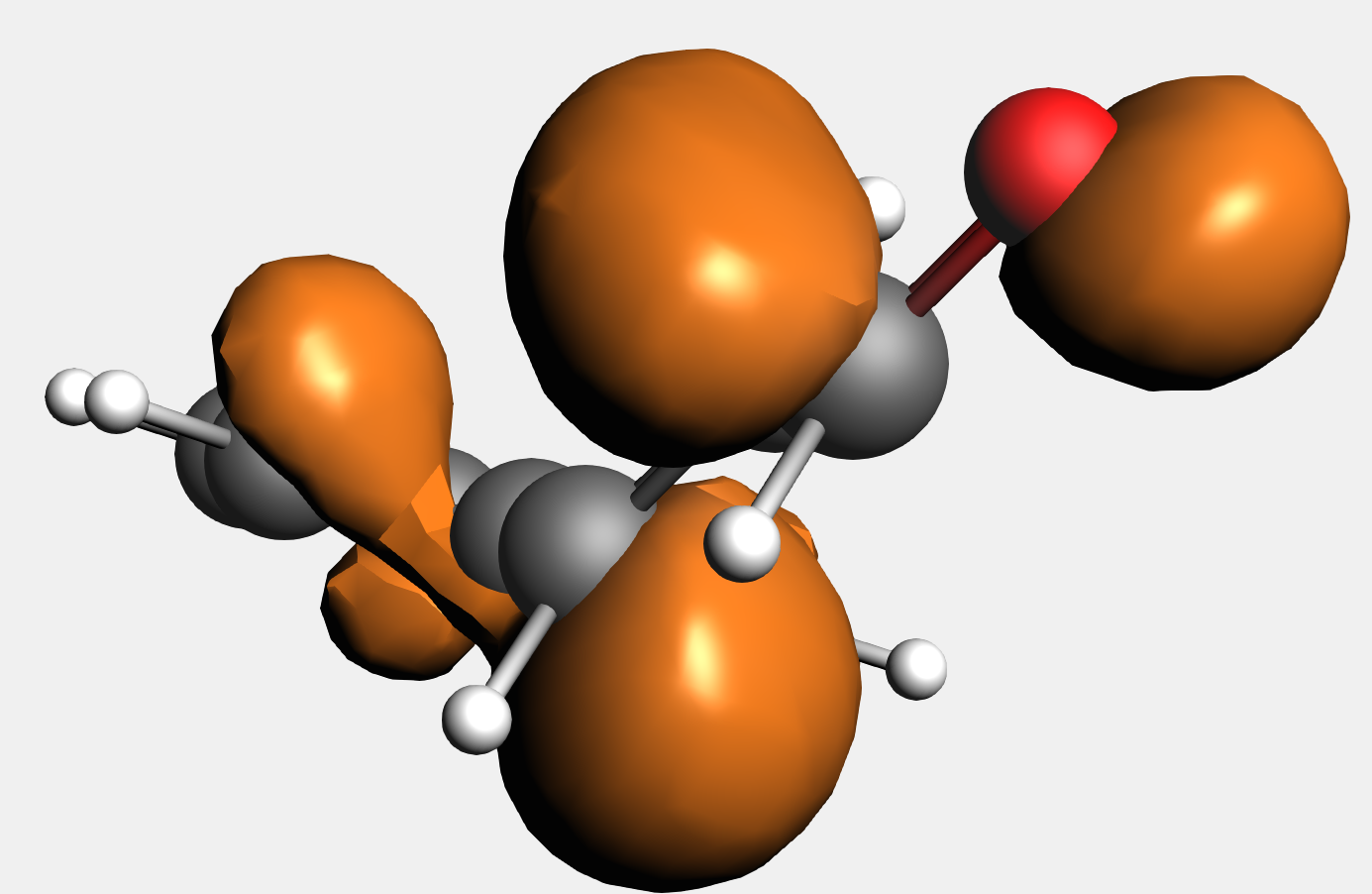

Supplement: S7 Data — (ZIP) [file pone.0343965.s008.zip › PONE-D-25-51583/Vitex Raw material/DFT Vitex all data/comp6/LL.png]

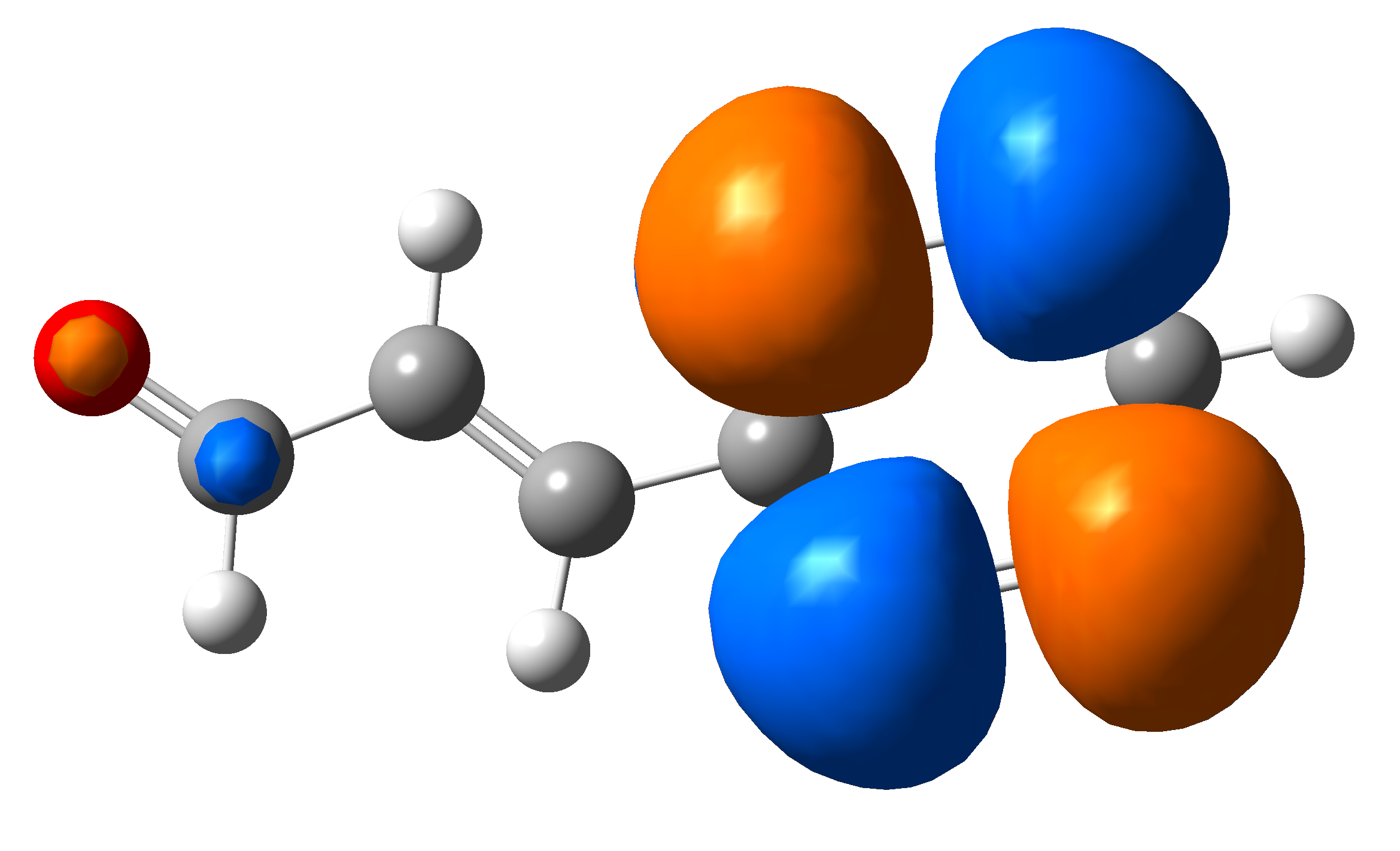

Supplement: S7 Data — (ZIP) [file pone.0343965.s008.zip › PONE-D-25-51583/Vitex Raw material/DFT Vitex all data/comp6/lomo+1.tif]

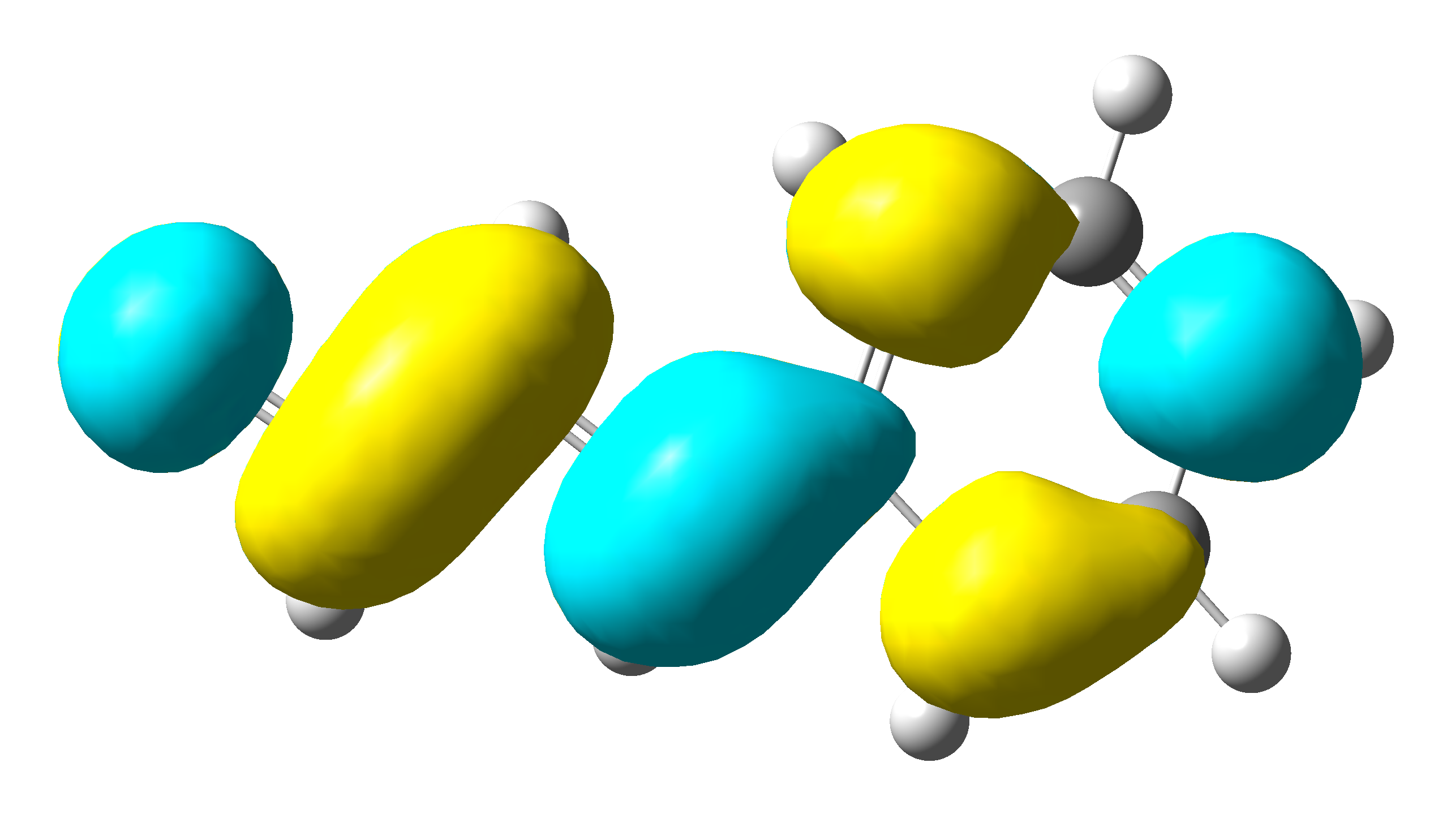

Supplement: S7 Data — (ZIP) [file pone.0343965.s008.zip › PONE-D-25-51583/Vitex Raw material/DFT Vitex all data/comp6/lomo.tif]

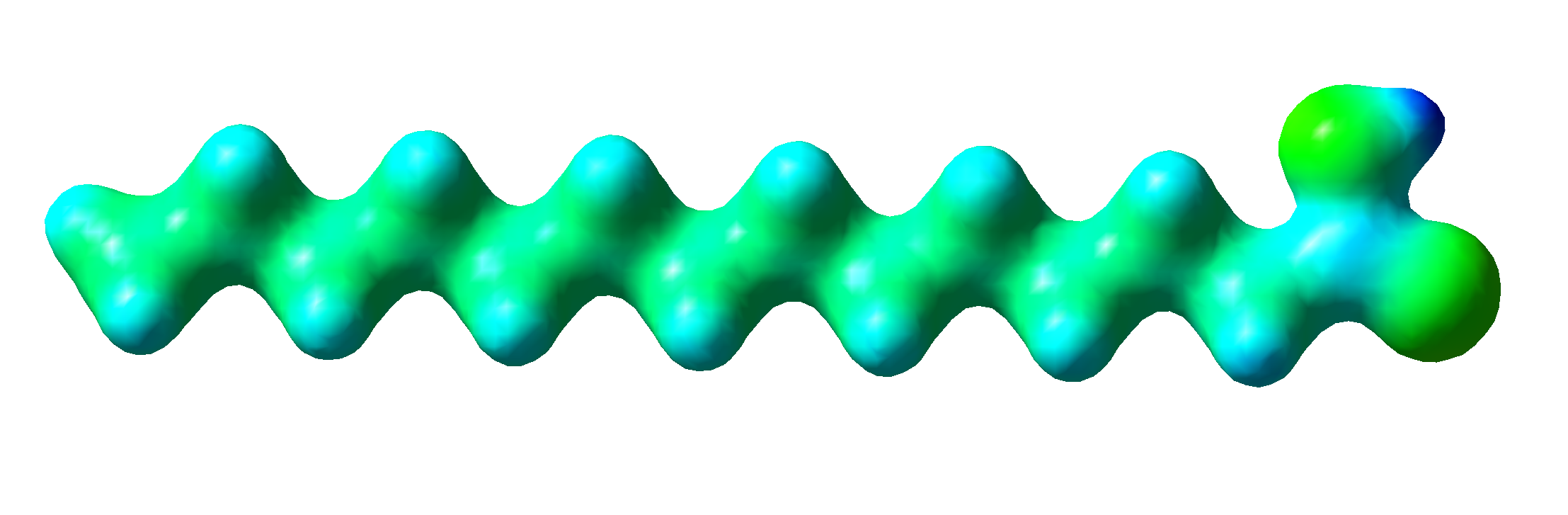

Supplement: S7 Data — (ZIP) [file pone.0343965.s008.zip › PONE-D-25-51583/Vitex Raw material/DFT Vitex all data/comp7/c7.tif]

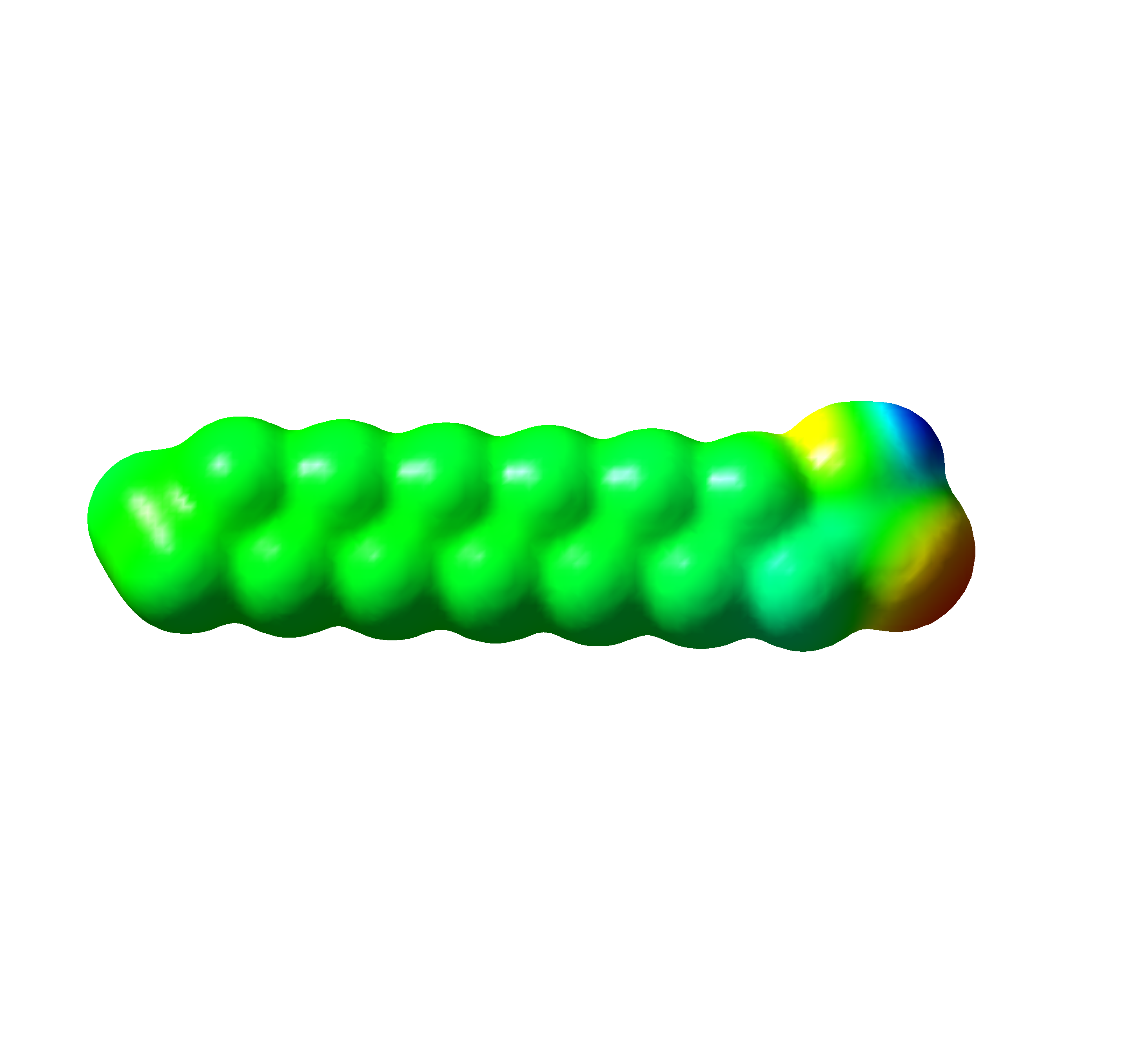

Supplement: S7 Data — (ZIP) [file pone.0343965.s008.zip › PONE-D-25-51583/Vitex Raw material/DFT Vitex all data/comp7/comp7.tif]

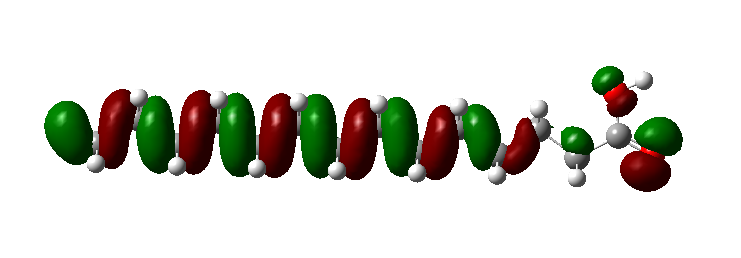

Supplement: S7 Data — (ZIP) [file pone.0343965.s008.zip › PONE-D-25-51583/Vitex Raw material/DFT Vitex all data/comp7/homo-1.tif]

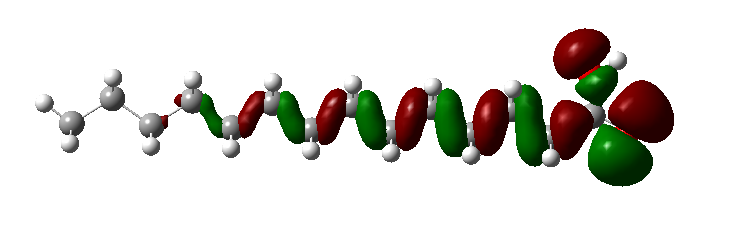

Supplement: S7 Data — (ZIP) [file pone.0343965.s008.zip › PONE-D-25-51583/Vitex Raw material/DFT Vitex all data/comp7/homo.tif]

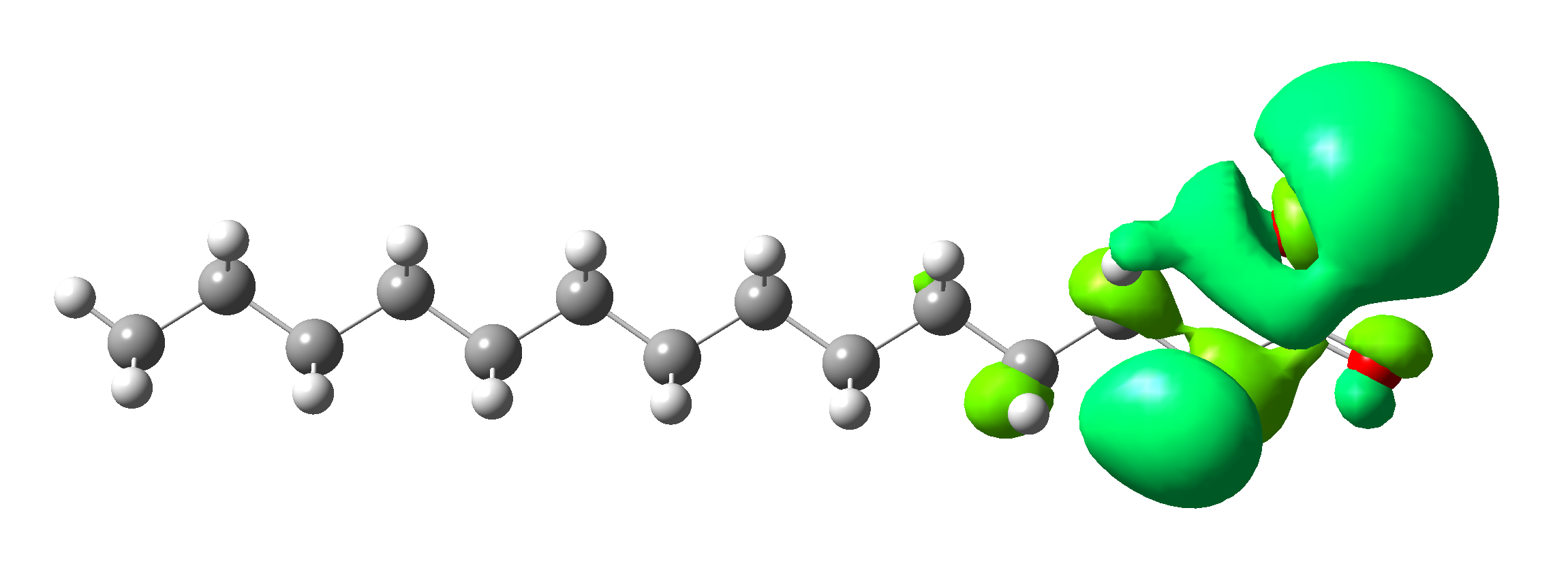

Supplement: S7 Data — (ZIP) [file pone.0343965.s008.zip › PONE-D-25-51583/Vitex Raw material/DFT Vitex all data/comp7/lomo+1.tif]

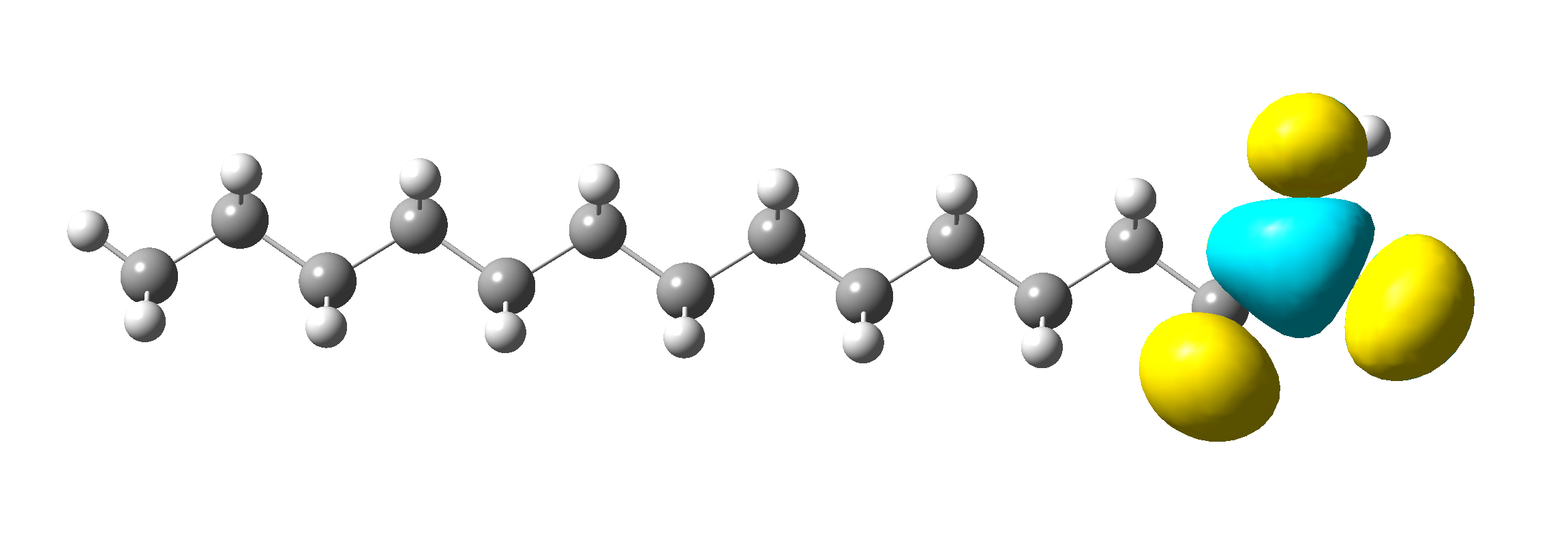

Supplement: S7 Data — (ZIP) [file pone.0343965.s008.zip › PONE-D-25-51583/Vitex Raw material/DFT Vitex all data/comp7/lomo.tif]

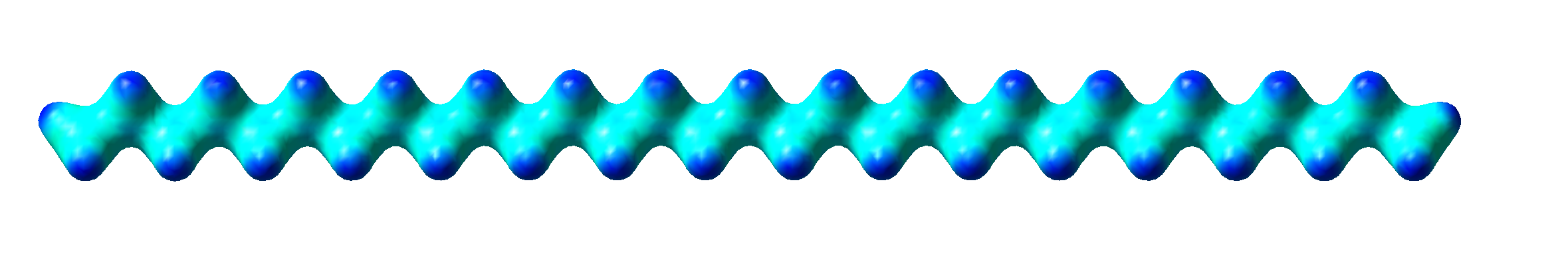

Supplement: S8 Data — (ZIP) [file pone.0343965.s009.zip › PONE-D-25-51583/Vitex Raw material/DFT Vitex all data/comp10/c10.tif]

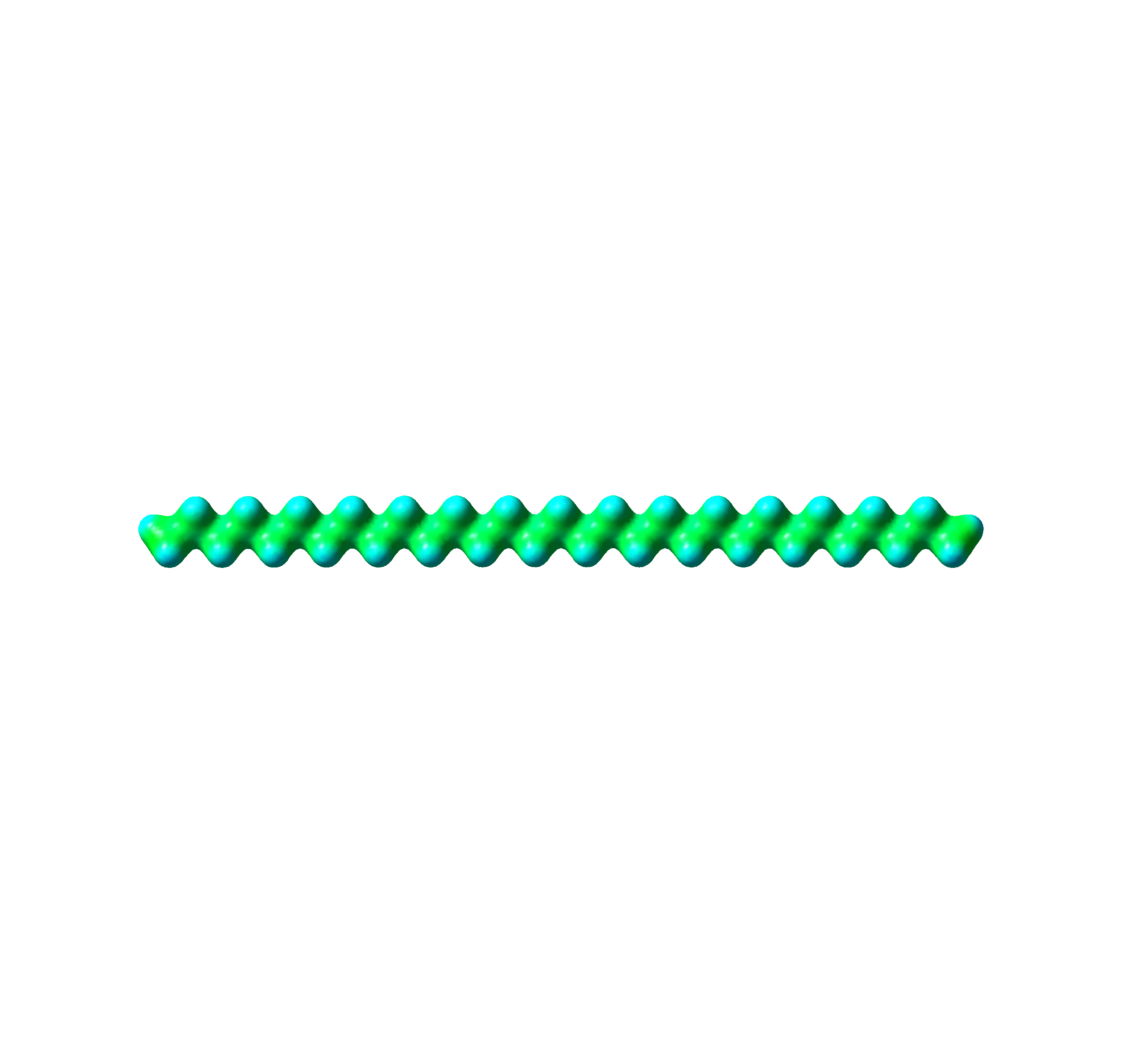

Supplement: S8 Data — (ZIP) [file pone.0343965.s009.zip › PONE-D-25-51583/Vitex Raw material/DFT Vitex all data/comp10/cc10.tif]

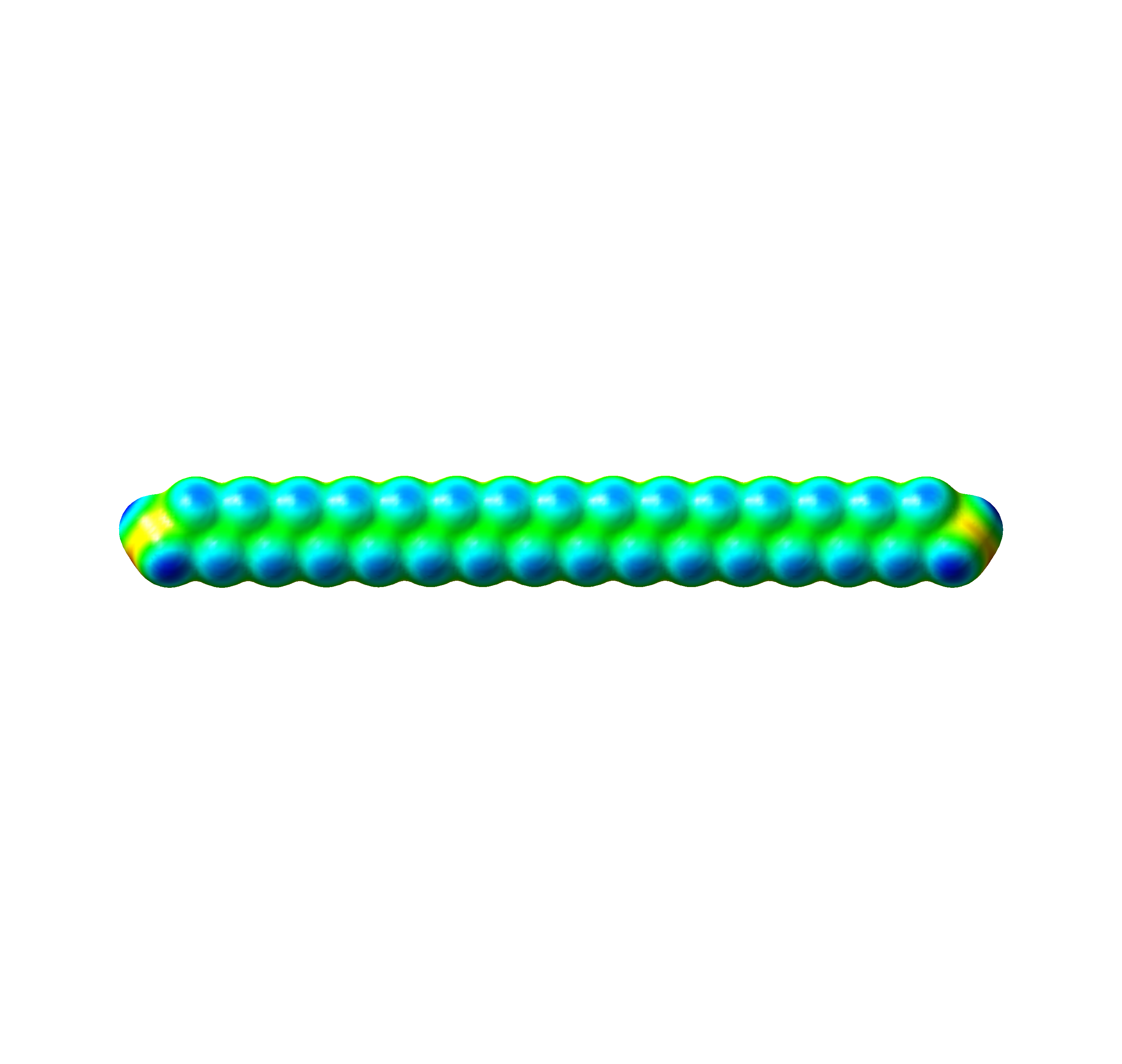

Supplement: S8 Data — (ZIP) [file pone.0343965.s009.zip › PONE-D-25-51583/Vitex Raw material/DFT Vitex all data/comp10/comp10.tif]

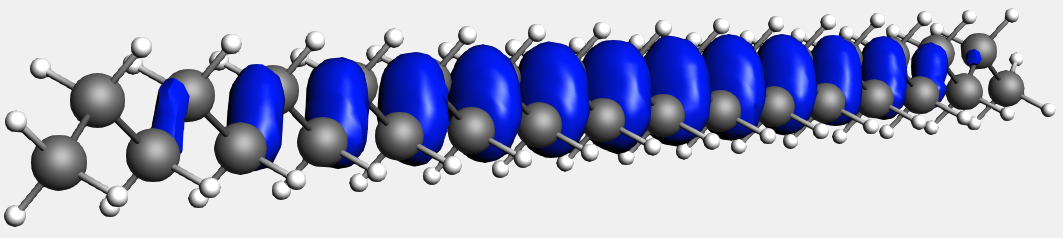

Supplement: S8 Data — (ZIP) [file pone.0343965.s009.zip › PONE-D-25-51583/Vitex Raw material/DFT Vitex all data/comp10/h.png]

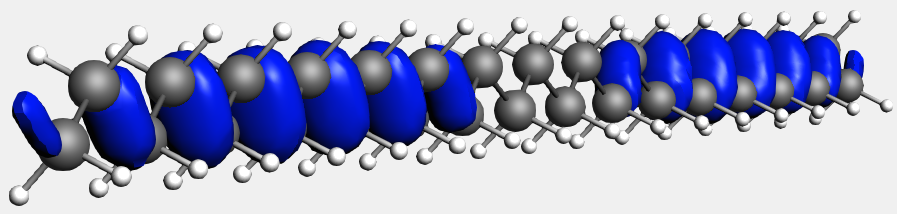

Supplement: S8 Data — (ZIP) [file pone.0343965.s009.zip › PONE-D-25-51583/Vitex Raw material/DFT Vitex all data/comp10/H1.png]

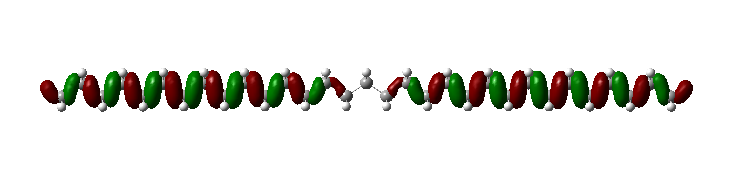

Supplement: S8 Data — (ZIP) [file pone.0343965.s009.zip › PONE-D-25-51583/Vitex Raw material/DFT Vitex all data/comp10/homo-1.tif]

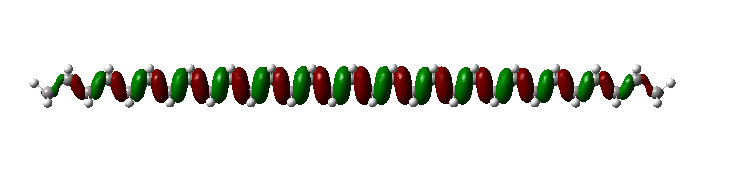

Supplement: S8 Data — (ZIP) [file pone.0343965.s009.zip › PONE-D-25-51583/Vitex Raw material/DFT Vitex all data/comp10/homo.tif]

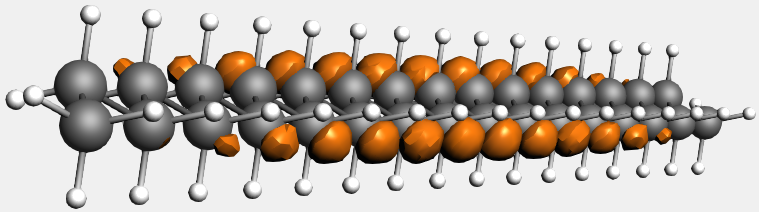

Supplement: S8 Data — (ZIP) [file pone.0343965.s009.zip › PONE-D-25-51583/Vitex Raw material/DFT Vitex all data/comp10/L.png]

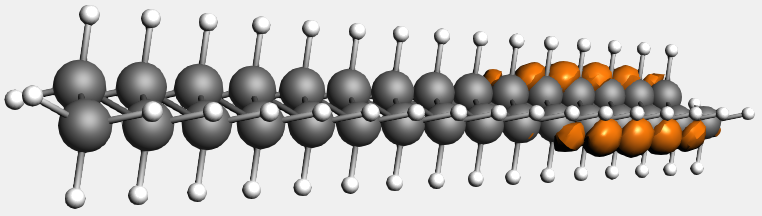

Supplement: S8 Data — (ZIP) [file pone.0343965.s009.zip › PONE-D-25-51583/Vitex Raw material/DFT Vitex all data/comp10/L1.png]

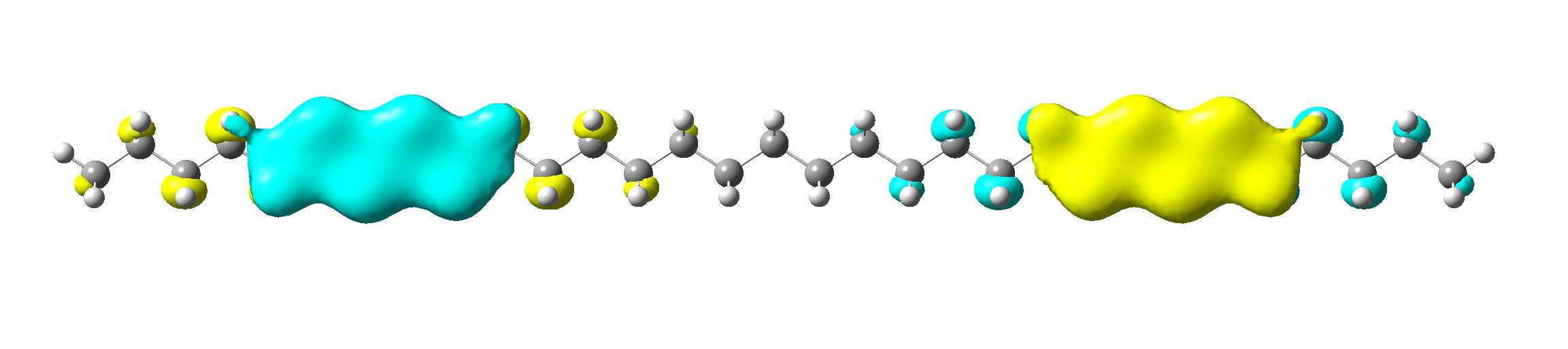

Supplement: S8 Data — (ZIP) [file pone.0343965.s009.zip › PONE-D-25-51583/Vitex Raw material/DFT Vitex all data/comp10/lomo+1.tif]

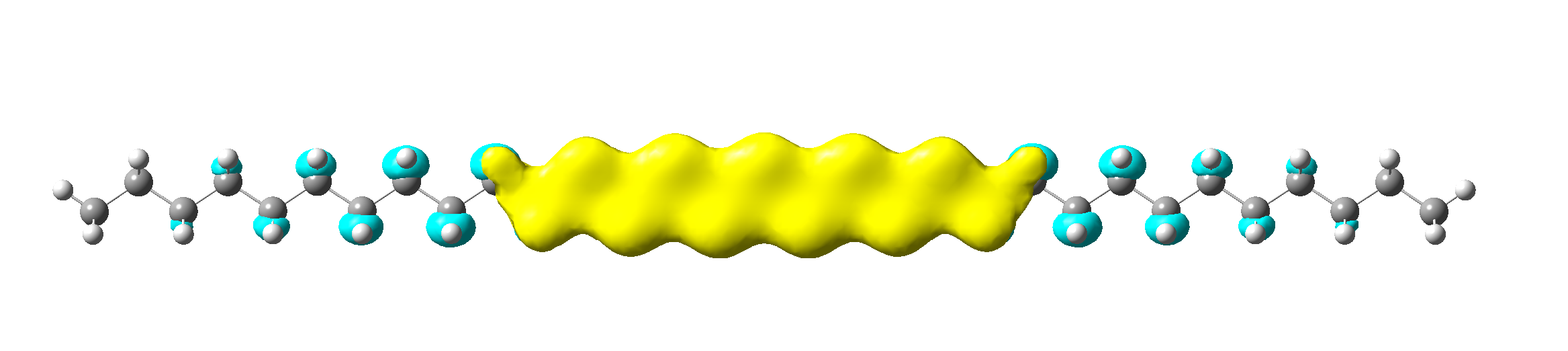

Supplement: S8 Data — (ZIP) [file pone.0343965.s009.zip › PONE-D-25-51583/Vitex Raw material/DFT Vitex all data/comp10/lomo.tif]

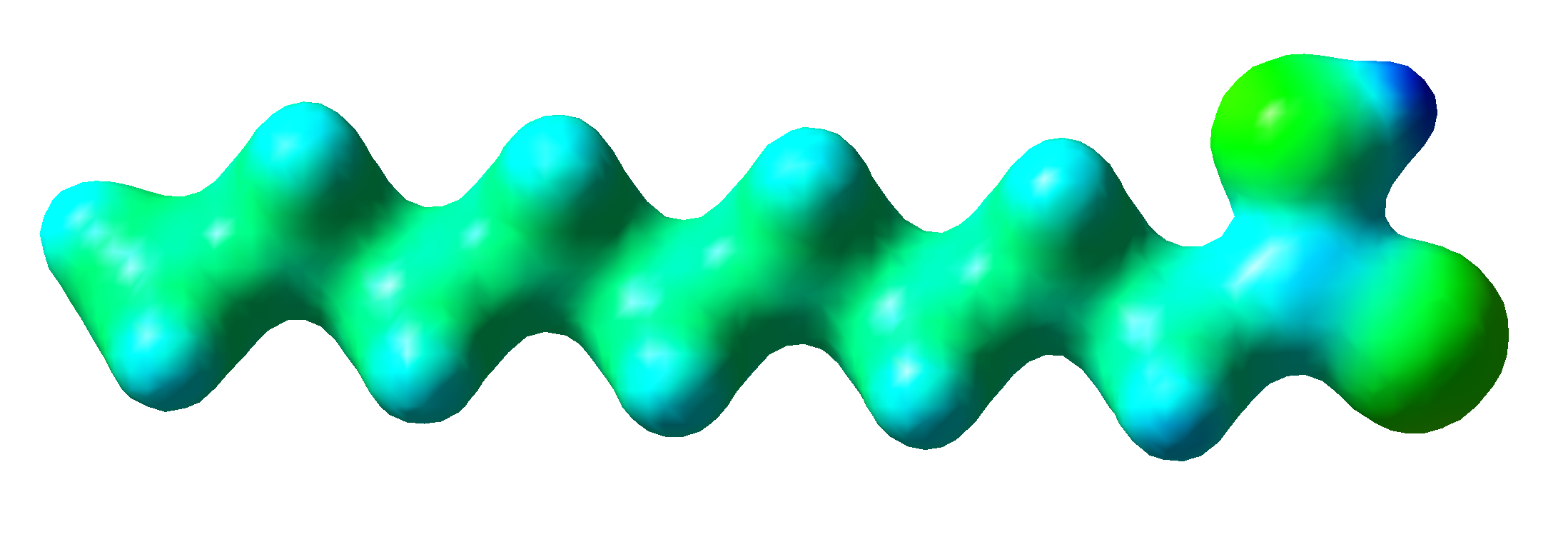

Supplement: S8 Data — (ZIP) [file pone.0343965.s009.zip › PONE-D-25-51583/Vitex Raw material/DFT Vitex all data/comp8/c8.tif]

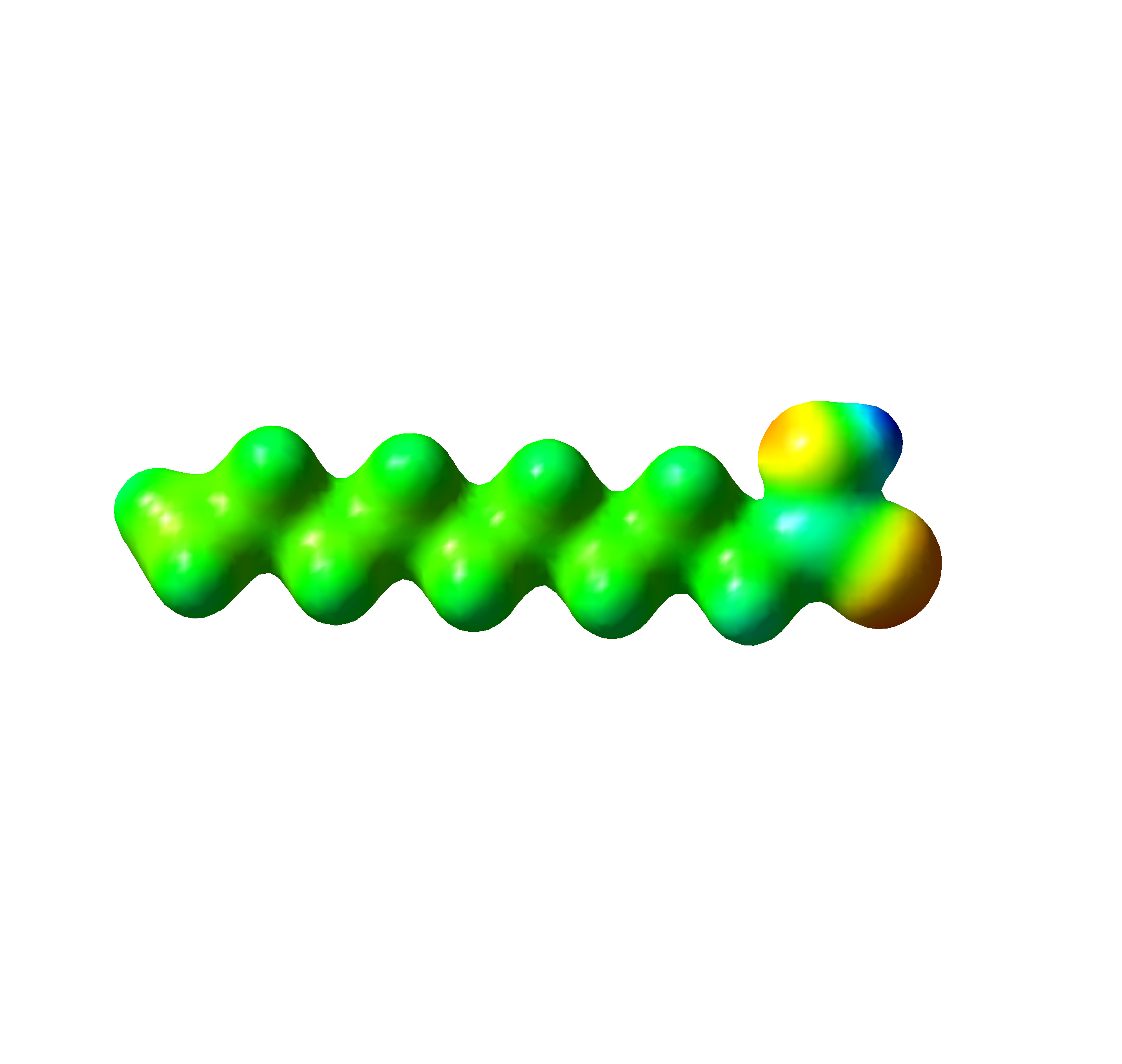

Supplement: S8 Data — (ZIP) [file pone.0343965.s009.zip › PONE-D-25-51583/Vitex Raw material/DFT Vitex all data/comp8/cc8.tif]

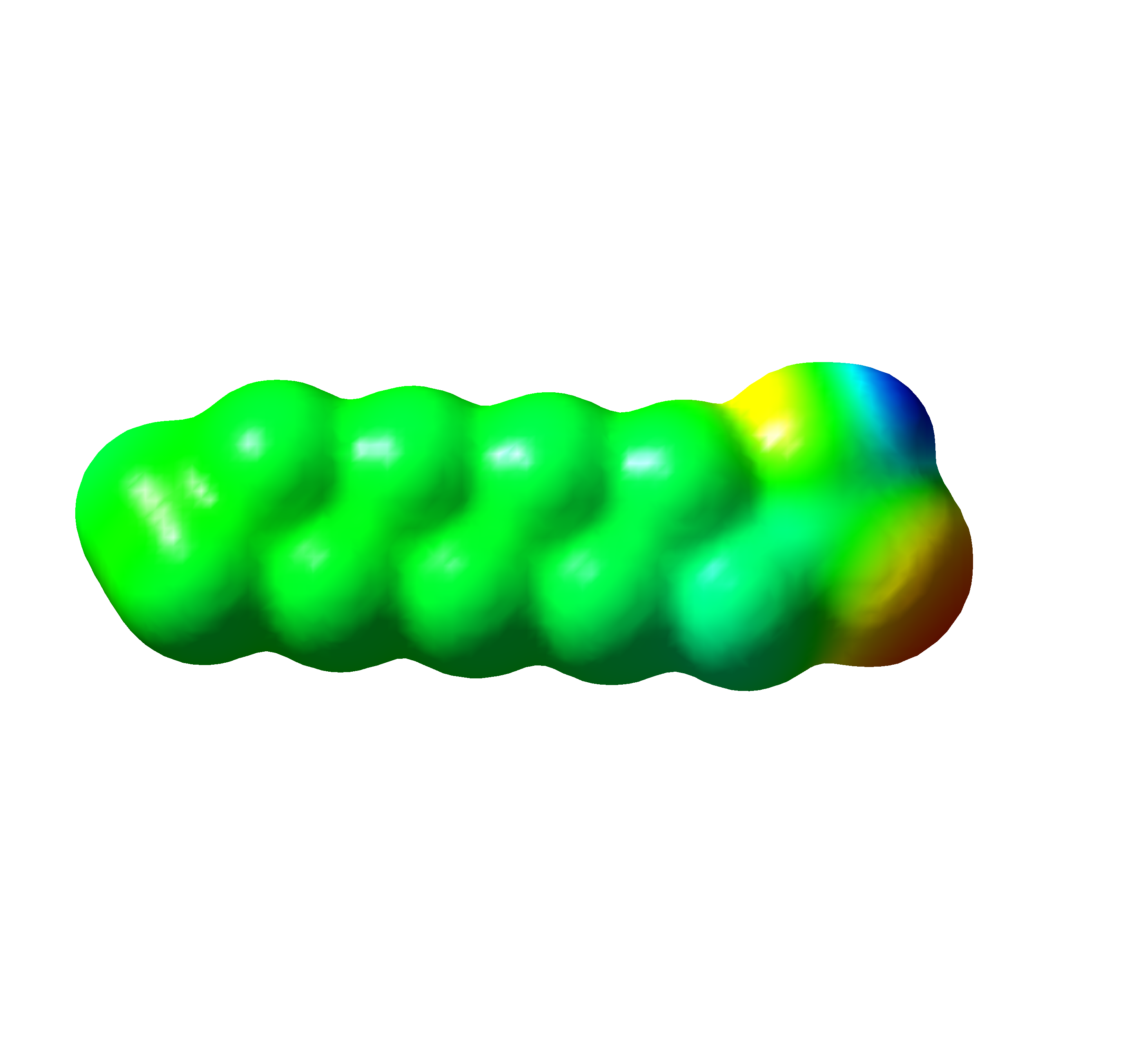

Supplement: S8 Data — (ZIP) [file pone.0343965.s009.zip › PONE-D-25-51583/Vitex Raw material/DFT Vitex all data/comp8/comp8.tif]

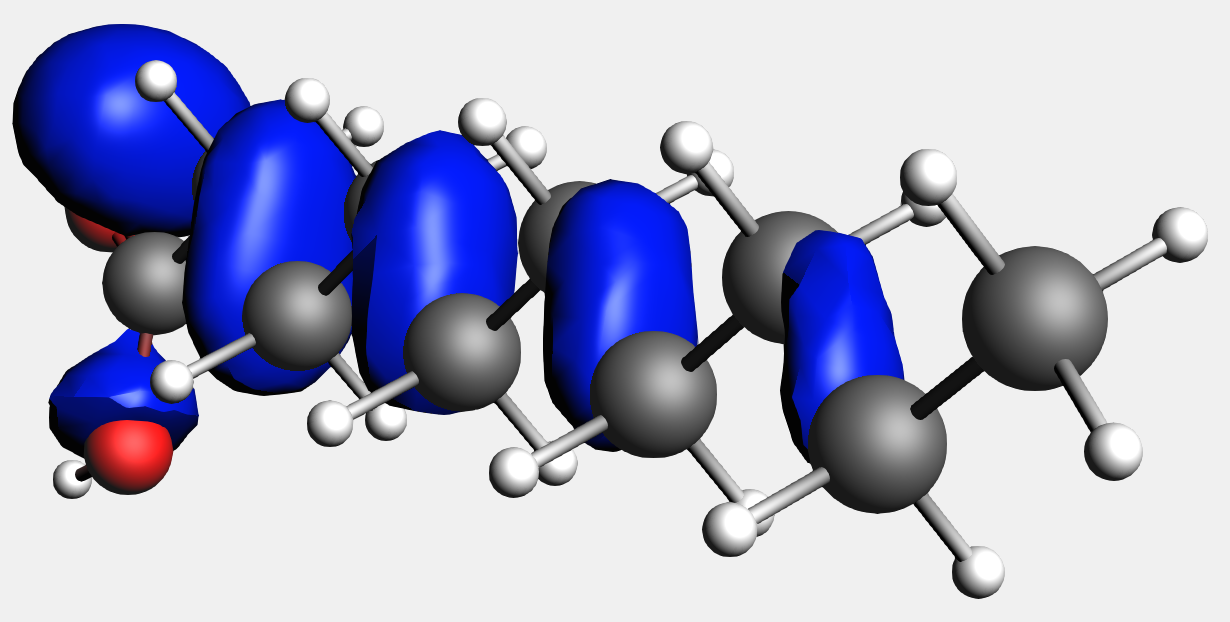

Supplement: S8 Data — (ZIP) [file pone.0343965.s009.zip › PONE-D-25-51583/Vitex Raw material/DFT Vitex all data/comp8/H.png]

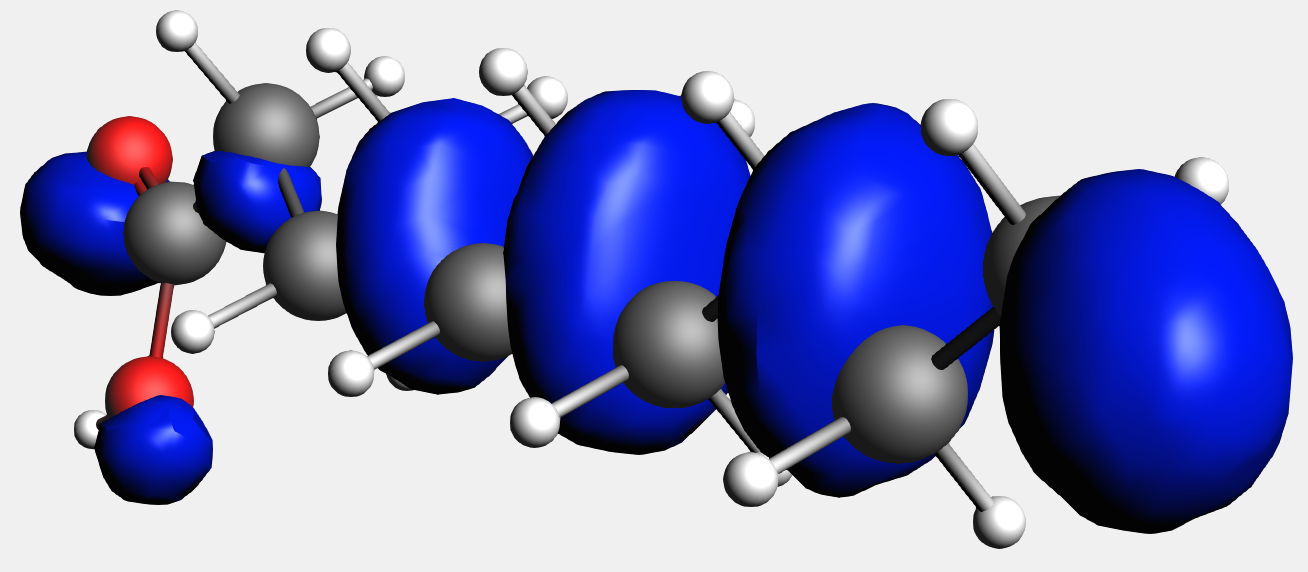

Supplement: S8 Data — (ZIP) [file pone.0343965.s009.zip › PONE-D-25-51583/Vitex Raw material/DFT Vitex all data/comp8/H1.png]

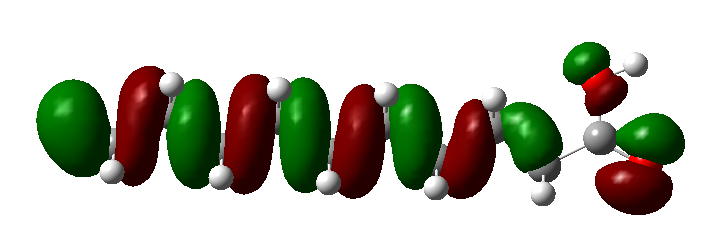

Supplement: S8 Data — (ZIP) [file pone.0343965.s009.zip › PONE-D-25-51583/Vitex Raw material/DFT Vitex all data/comp8/homo-1.tif]

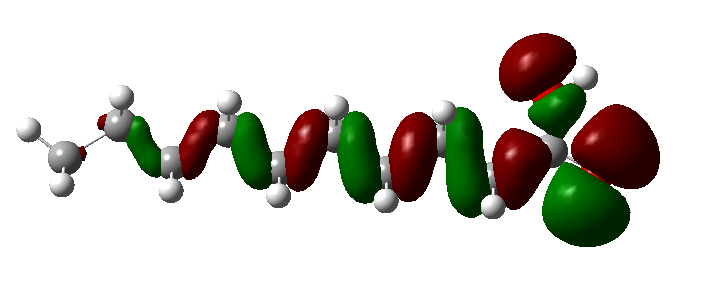

Supplement: S8 Data — (ZIP) [file pone.0343965.s009.zip › PONE-D-25-51583/Vitex Raw material/DFT Vitex all data/comp8/homo.tif]

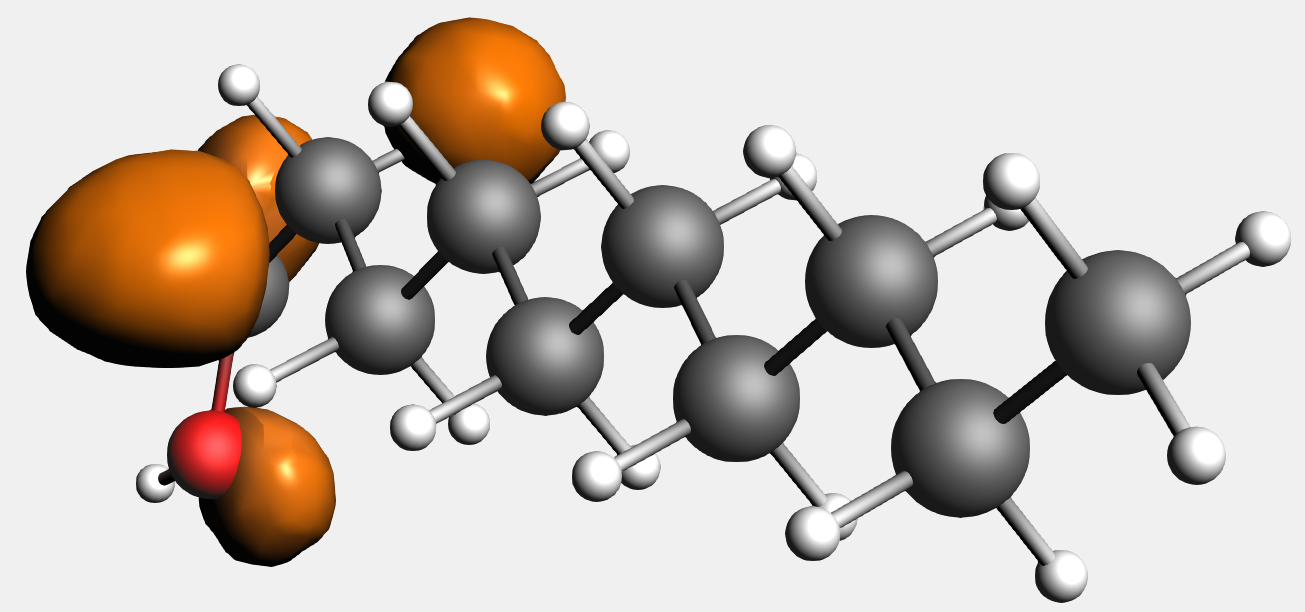

Supplement: S8 Data — (ZIP) [file pone.0343965.s009.zip › PONE-D-25-51583/Vitex Raw material/DFT Vitex all data/comp8/L.png]

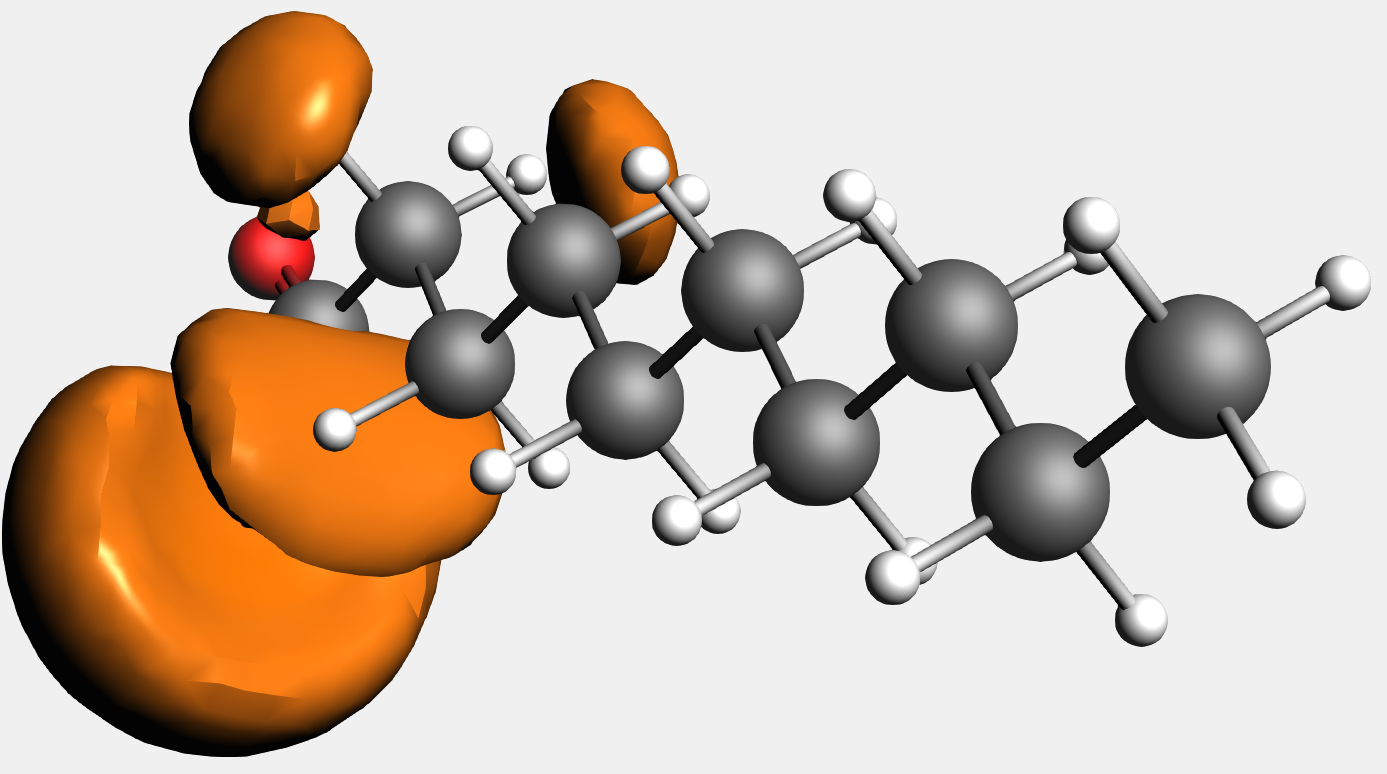

Supplement: S8 Data — (ZIP) [file pone.0343965.s009.zip › PONE-D-25-51583/Vitex Raw material/DFT Vitex all data/comp8/L1.png]

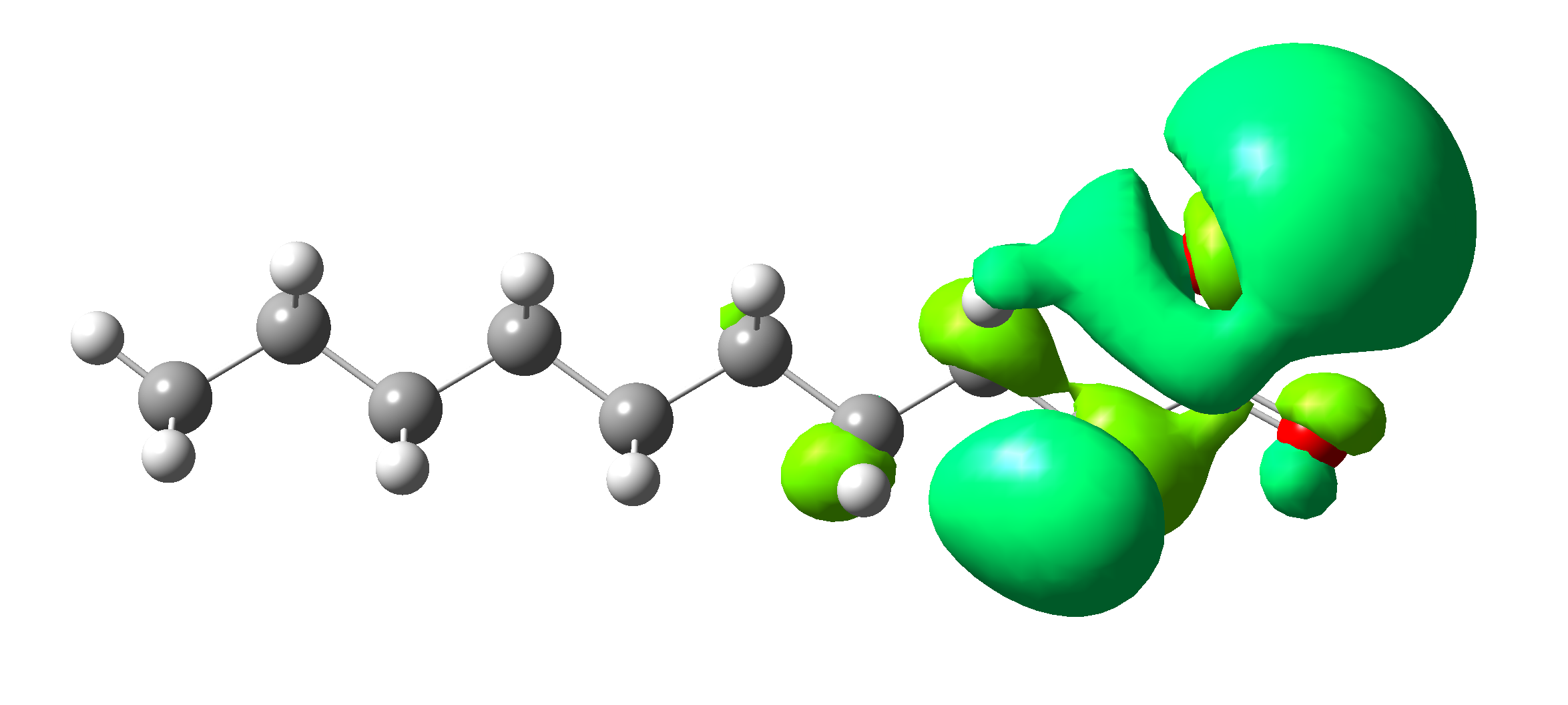

Supplement: S8 Data — (ZIP) [file pone.0343965.s009.zip › PONE-D-25-51583/Vitex Raw material/DFT Vitex all data/comp8/lomo+1.tif]

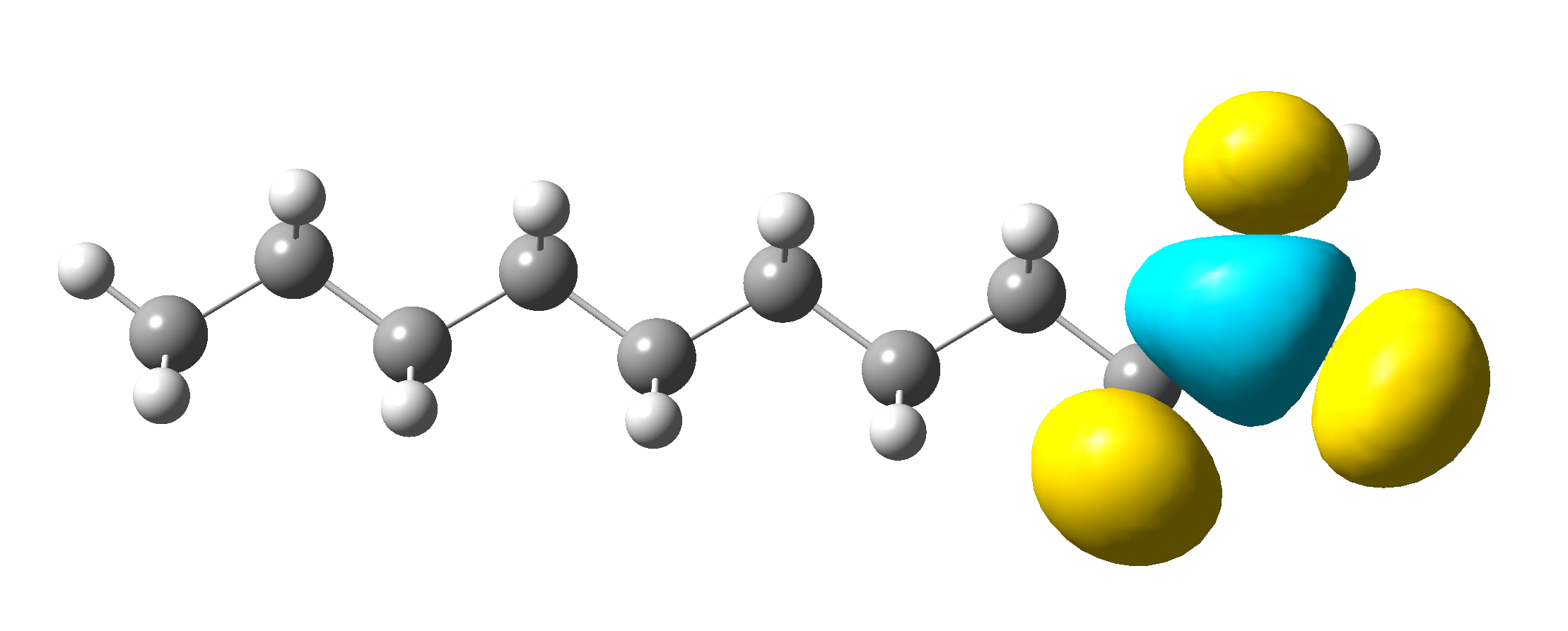

Supplement: S8 Data — (ZIP) [file pone.0343965.s009.zip › PONE-D-25-51583/Vitex Raw material/DFT Vitex all data/comp8/lomo.tif]

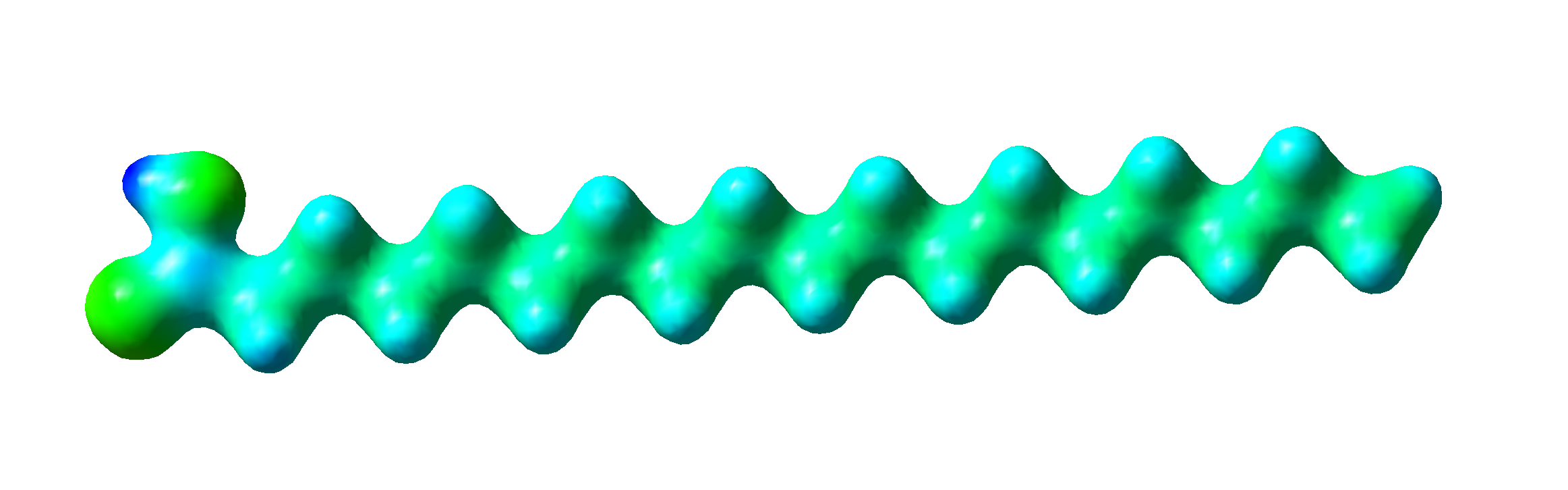

Supplement: S8 Data — (ZIP) [file pone.0343965.s009.zip › PONE-D-25-51583/Vitex Raw material/DFT Vitex all data/comp9/c9.tif]

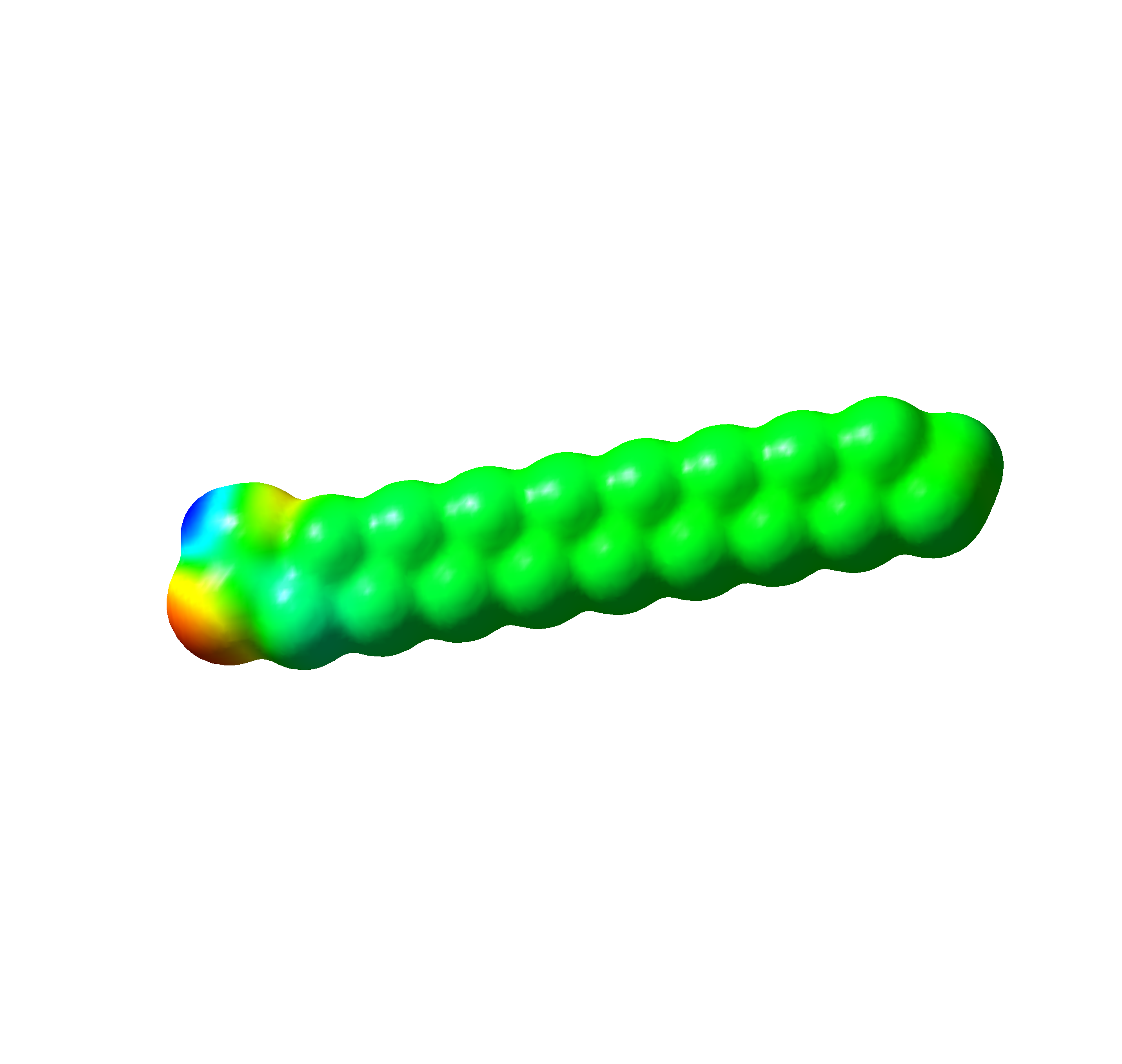

Supplement: S8 Data — (ZIP) [file pone.0343965.s009.zip › PONE-D-25-51583/Vitex Raw material/DFT Vitex all data/comp9/comp9.tif]

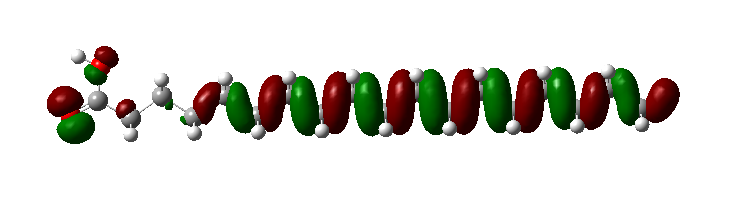

Supplement: S8 Data — (ZIP) [file pone.0343965.s009.zip › PONE-D-25-51583/Vitex Raw material/DFT Vitex all data/comp9/homo-1.tif]

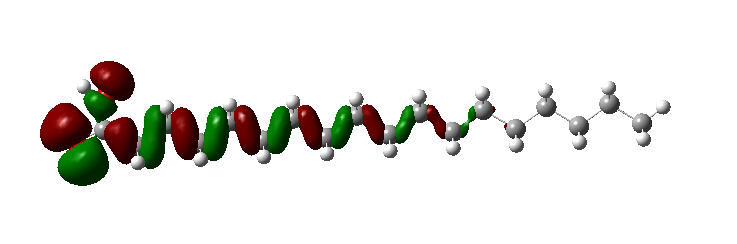

Supplement: S8 Data — (ZIP) [file pone.0343965.s009.zip › PONE-D-25-51583/Vitex Raw material/DFT Vitex all data/comp9/homo.tif]

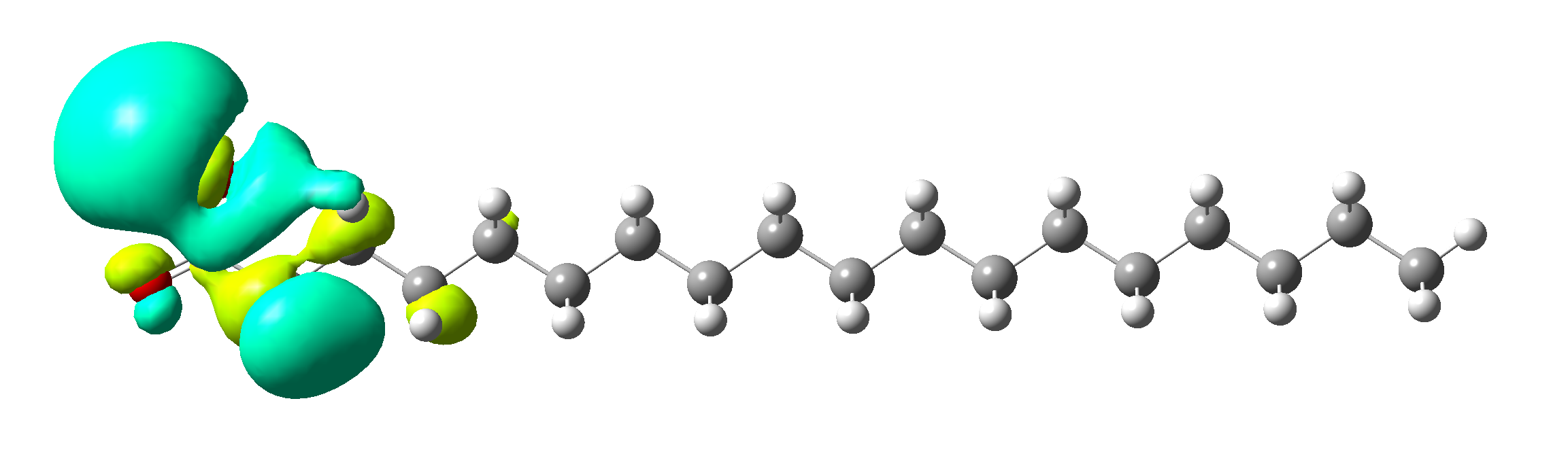

Supplement: S8 Data — (ZIP) [file pone.0343965.s009.zip › PONE-D-25-51583/Vitex Raw material/DFT Vitex all data/comp9/lomo+1.tif]

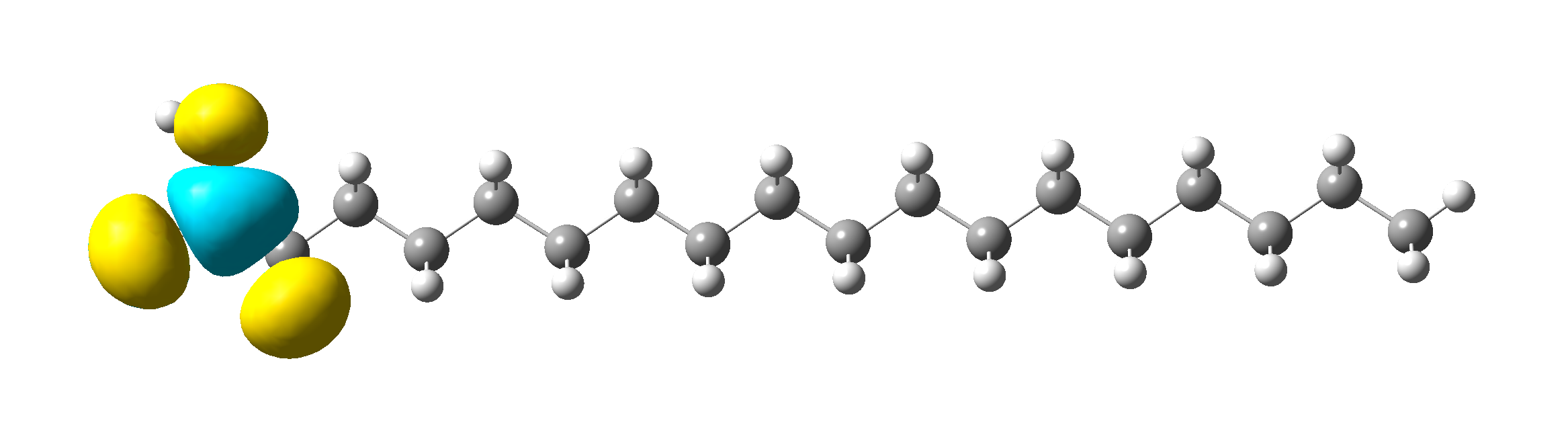

Supplement: S8 Data — (ZIP) [file pone.0343965.s009.zip › PONE-D-25-51583/Vitex Raw material/DFT Vitex all data/comp9/lomo.tif]

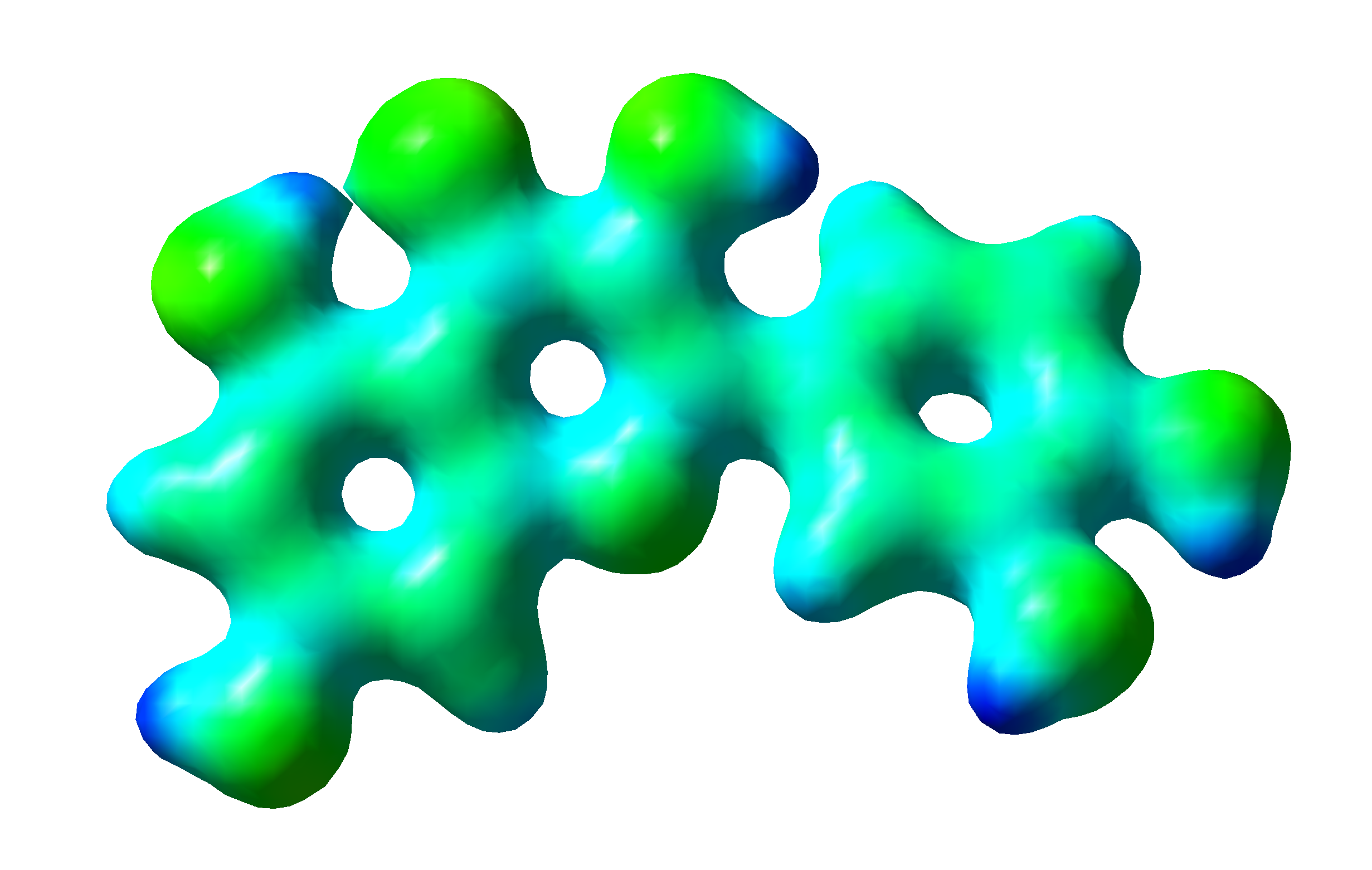

Supplement: S10 Data — (ZIP) [file pone.0343965.s011.zip › PONE-D-25-51583/Vitex Raw material/DFT Vitex all data/comp16/c16.tif]

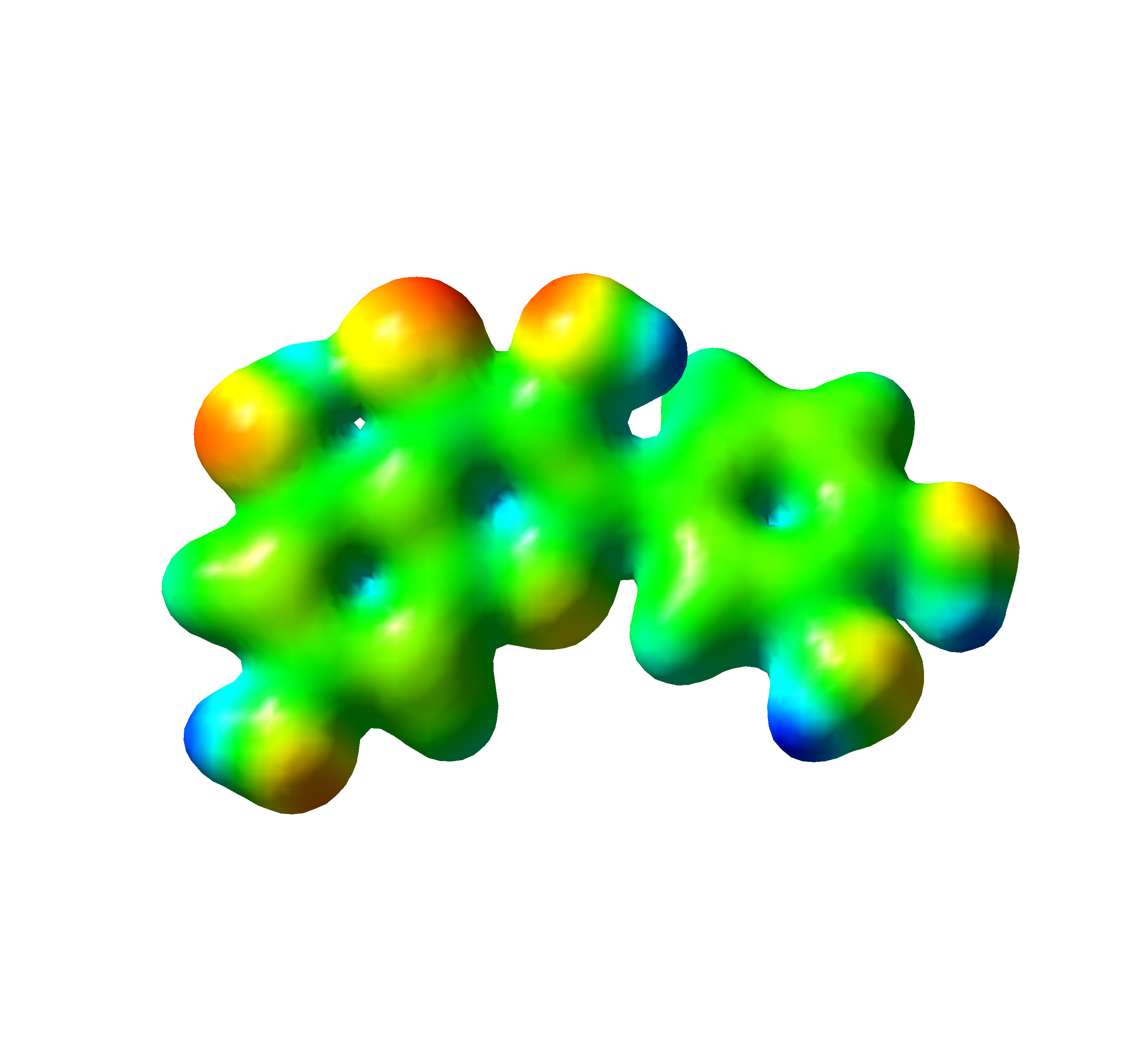

Supplement: S10 Data — (ZIP) [file pone.0343965.s011.zip › PONE-D-25-51583/Vitex Raw material/DFT Vitex all data/comp16/cc16.tif]

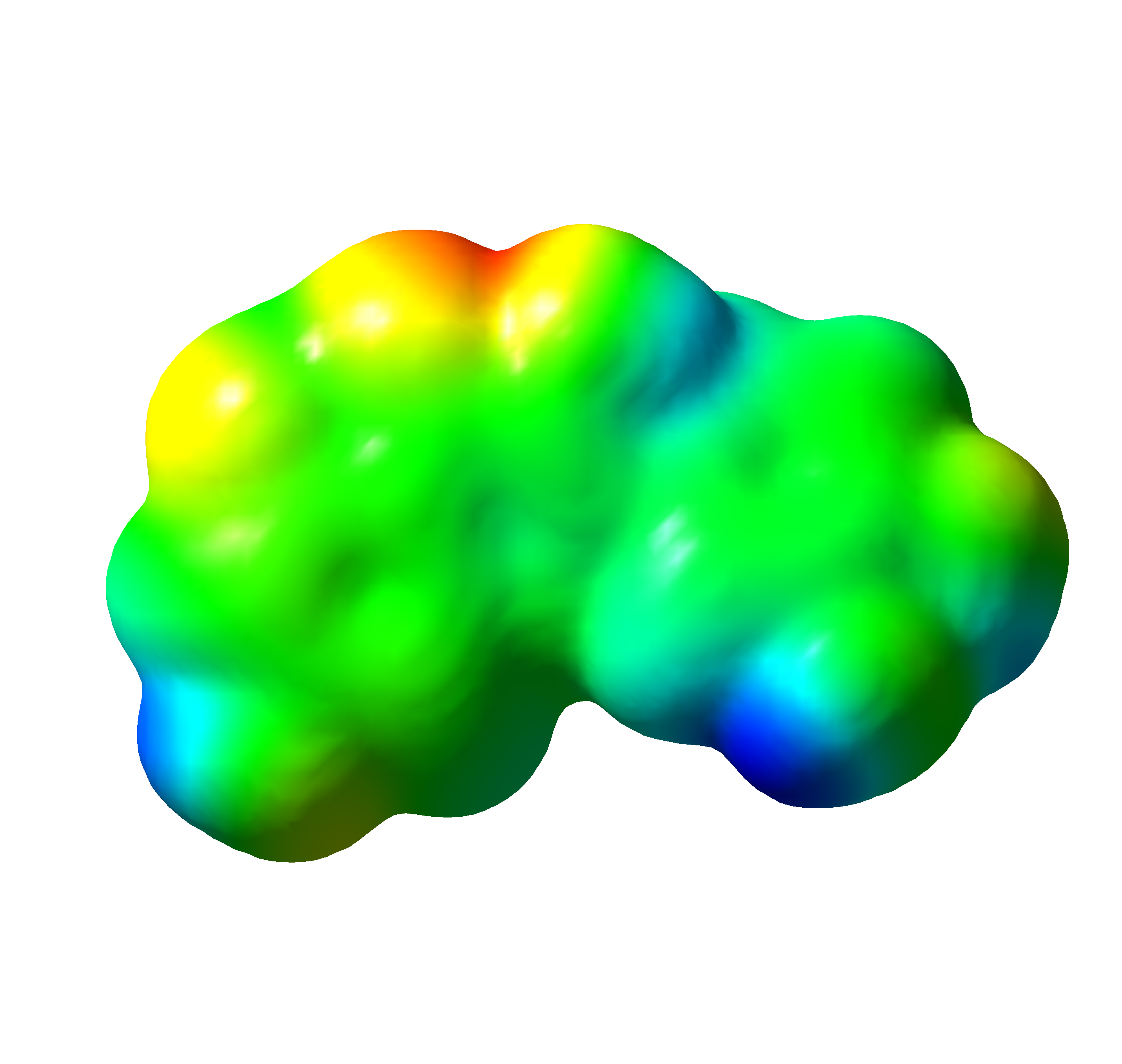

Supplement: S10 Data — (ZIP) [file pone.0343965.s011.zip › PONE-D-25-51583/Vitex Raw material/DFT Vitex all data/comp16/comp16.tif]

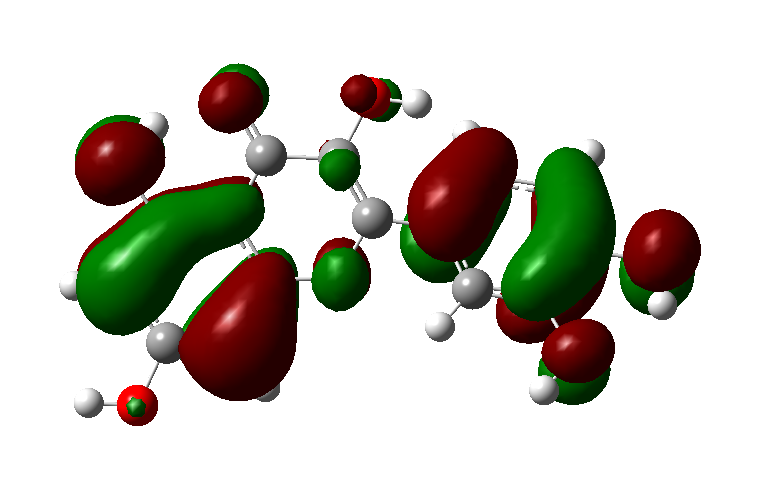

Supplement: S10 Data — (ZIP) [file pone.0343965.s011.zip › PONE-D-25-51583/Vitex Raw material/DFT Vitex all data/comp16/homo-1.tif]

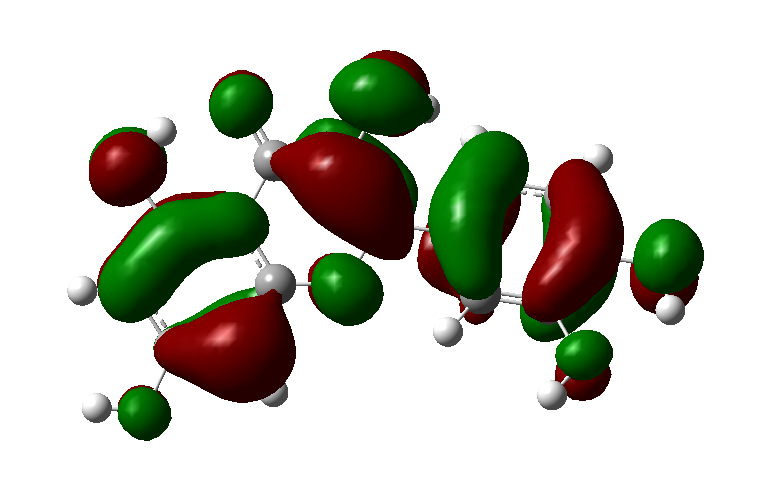

Supplement: S10 Data — (ZIP) [file pone.0343965.s011.zip › PONE-D-25-51583/Vitex Raw material/DFT Vitex all data/comp16/homo.tif]

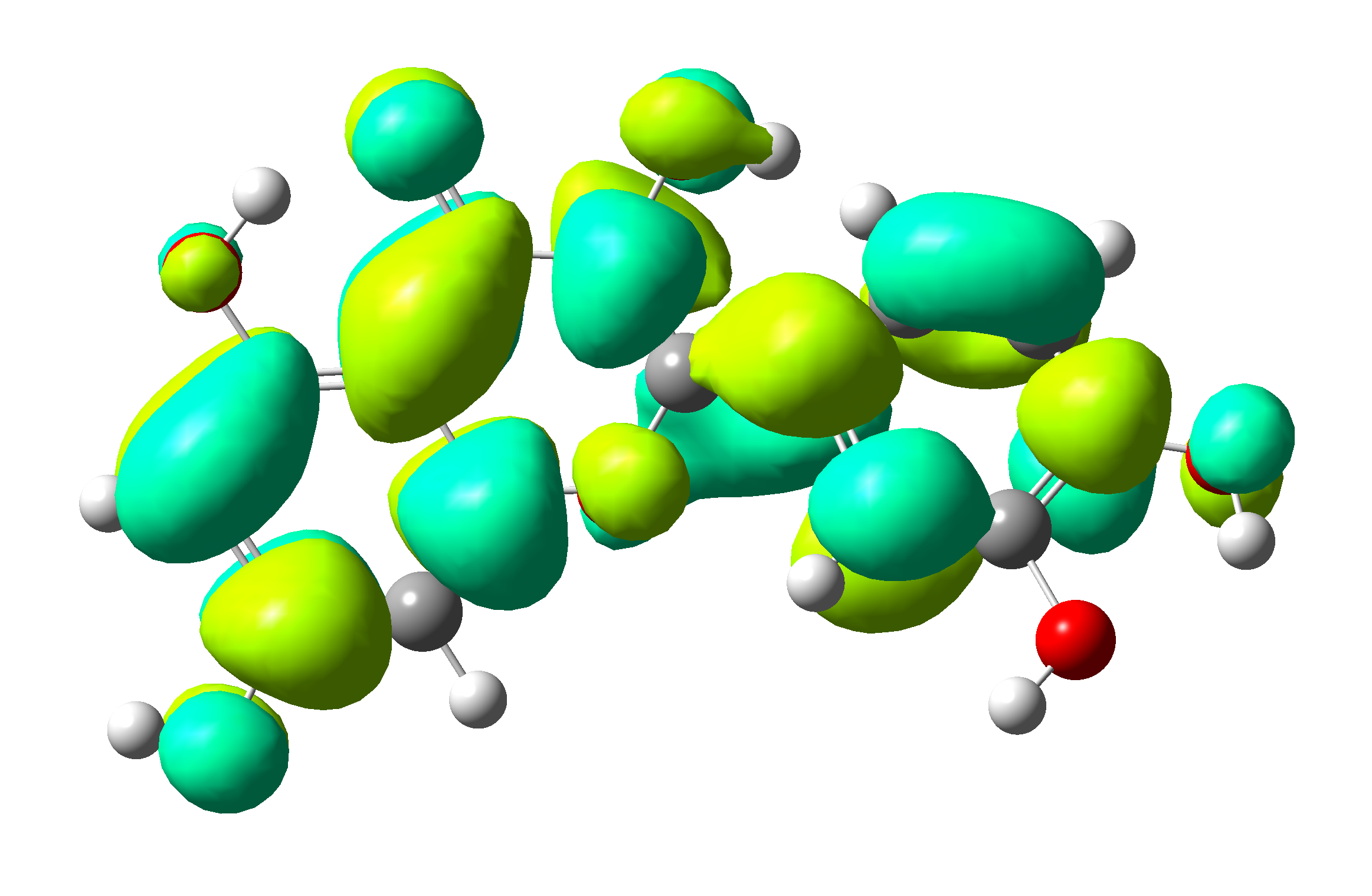

Supplement: S10 Data — (ZIP) [file pone.0343965.s011.zip › PONE-D-25-51583/Vitex Raw material/DFT Vitex all data/comp16/lomo+1.tif]

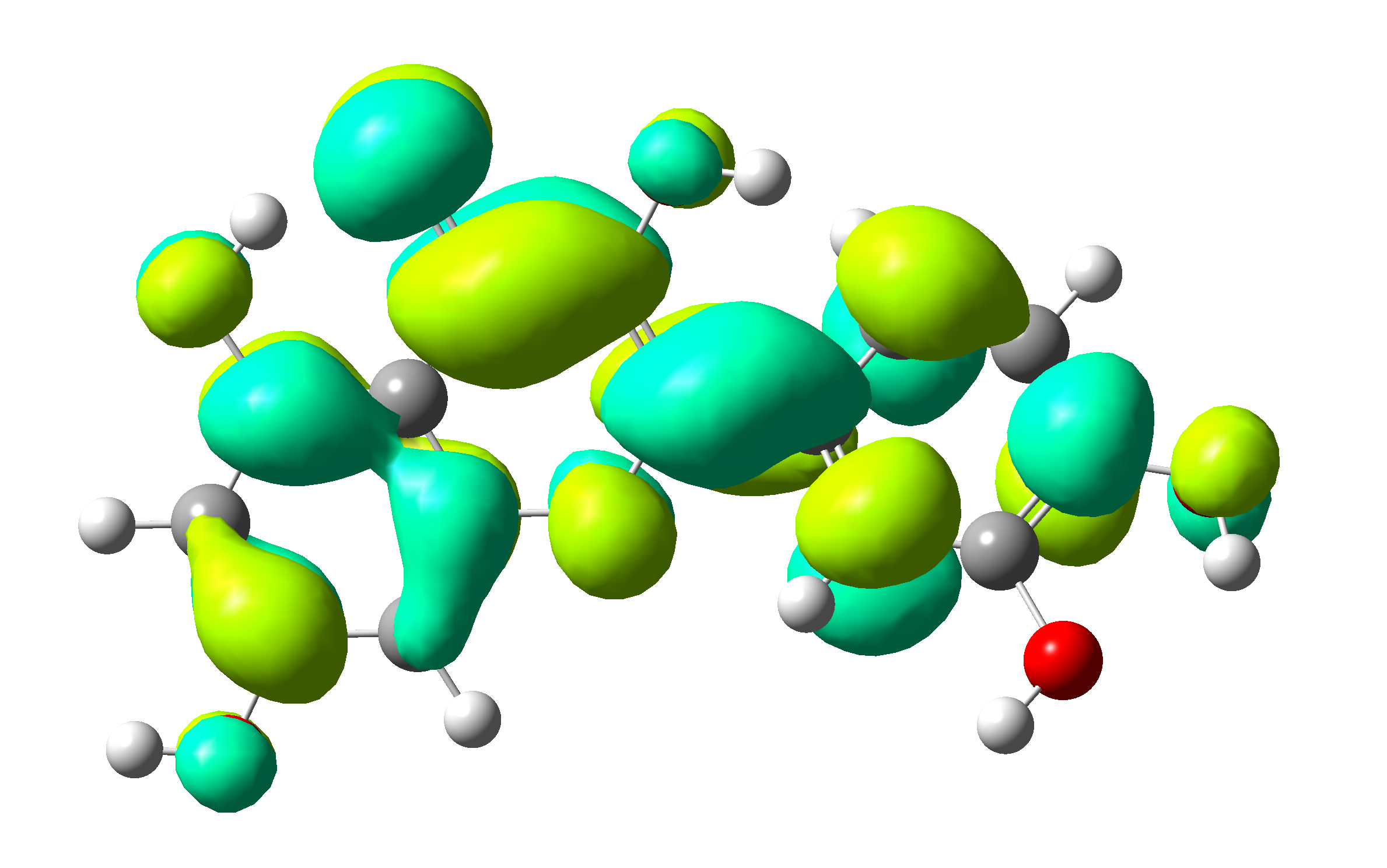

Supplement: S10 Data — (ZIP) [file pone.0343965.s011.zip › PONE-D-25-51583/Vitex Raw material/DFT Vitex all data/comp16/lomo.tif]

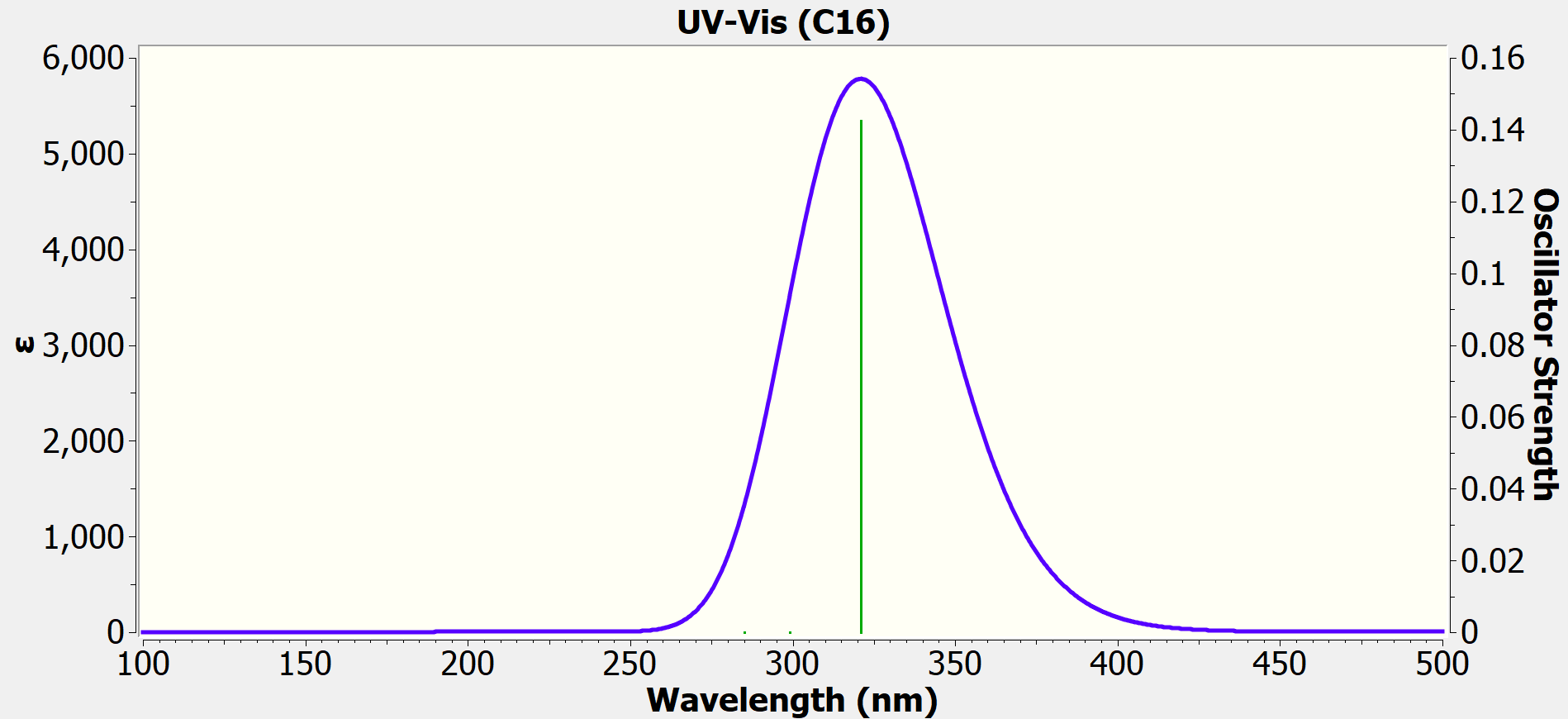

Supplement: S11 Data — (ZIP) [file pone.0343965.s012.zip › PONE-D-25-51583/Vitex Raw material/DFT Vitex all data/UV C16/33.png]

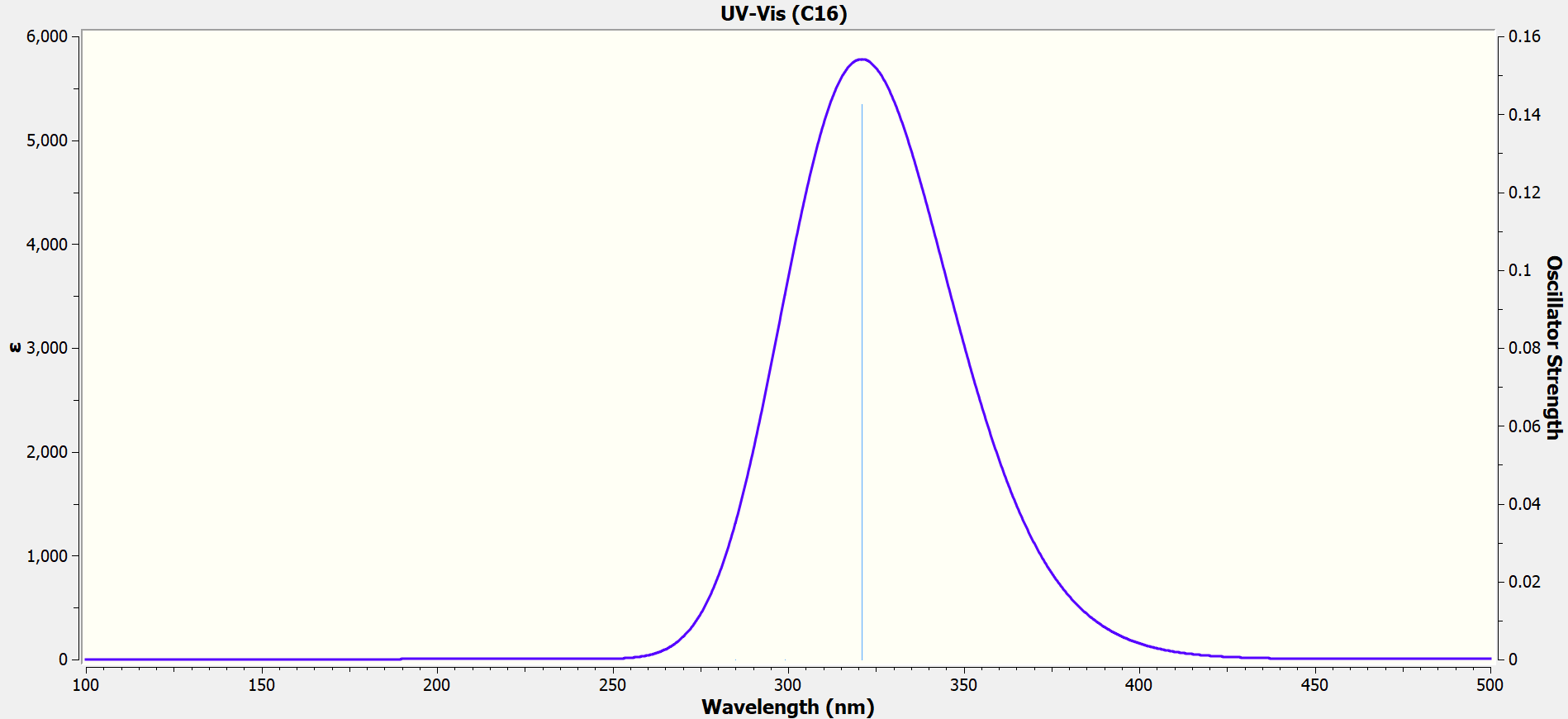

Supplement: S11 Data — (ZIP) [file pone.0343965.s012.zip › PONE-D-25-51583/Vitex Raw material/DFT Vitex all data/UV C16/QUERCITIN 16_uvvis.png]

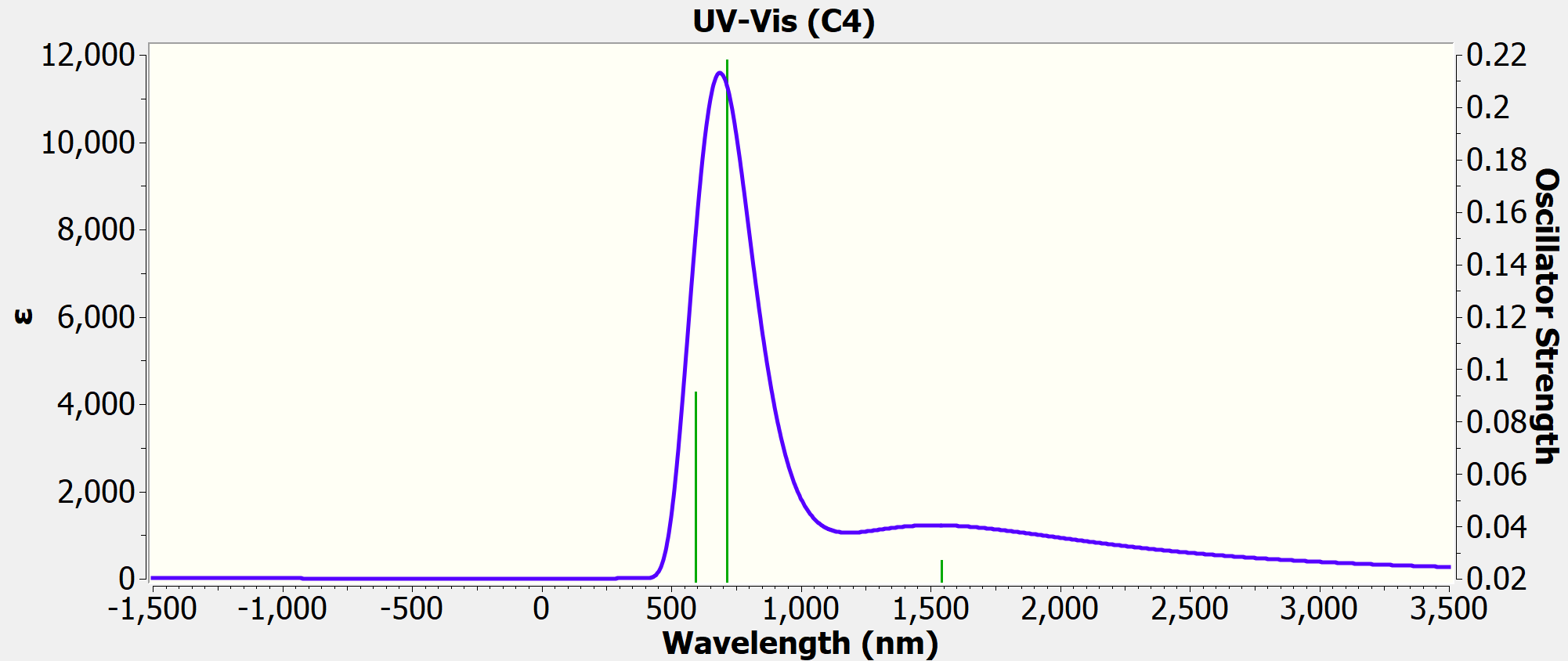

Supplement: S11 Data — (ZIP) [file pone.0343965.s012.zip › PONE-D-25-51583/Vitex Raw material/DFT Vitex all data/UV C4/33.png]

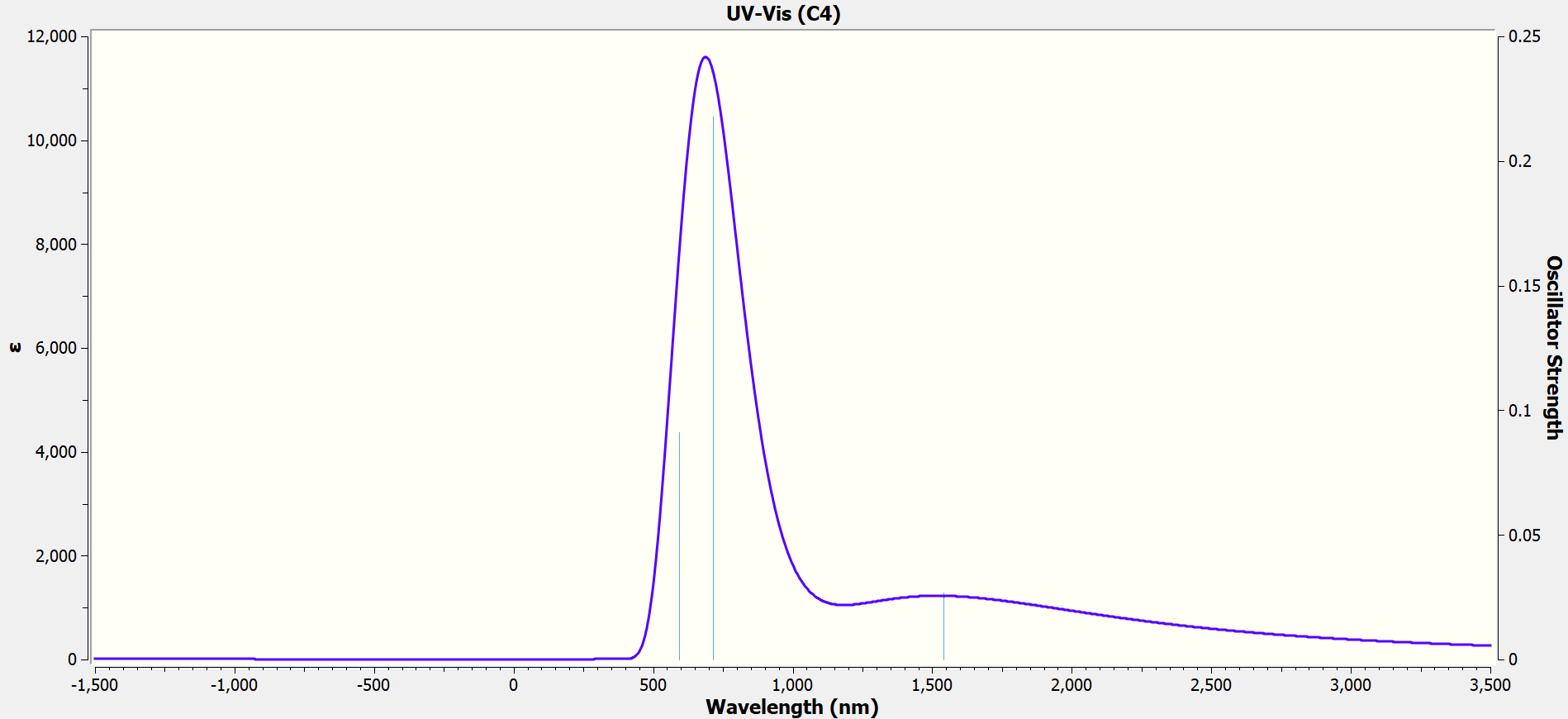

Supplement: S11 Data — (ZIP) [file pone.0343965.s012.zip › PONE-D-25-51583/Vitex Raw material/DFT Vitex all data/UV C4/New Folder/COMP4 LOG_uvvis.png]
