# Supplementary material for: Phytochemical profiling of Vitex negundo seeds via UHPLC-QTOF-MS/MS analyses with antimicrobial evaluation and in silico targeting of DNA Gyrase B and Secreted Aspartic Proteinase 2 (SAP2)
Source: PLoS One. 2026 Mar 13;21(3):e0343965. doi: 10.1371/journal.pone.0343965 (PMC12987476; doi:10.1371/journal.pone.0343965)
Supplement: S16 Data — (ZIP) [file pone.0343965.s017.zip › Vitex graph and bioactivity raw data/Vitex raw data for graph and bioactivity .docx]

**Vitex raw materials data:**

**The values used to build the graph in Fig. 4 (A):**

**Bioactivity against *Staphylococcus aureus***

| **Sample fractions** | **Type** | **Concentration (mg/ml)** | **Inhibition zone (mm)** |
| --- | --- | --- | --- |
| TH | MeOH seed extract | \| 0.30 \| \| --- \| | 26.5 |
| TL | MeOH seed extract | 0.15 | 27.0 |
| SH | Ciprofloxacin (standard) | 0.30 | 25.9 |
| SL | Ciprofloxacin (standard) | 0.15 | 26.2 |

**Table 2.** Anti-microbial activities of MeOH seeds extract of *Vitex negundo* vs ciprofloxacin (standard).

All standard fractions and sample values are mentioned in mg/ml; TH represents the high dilution, TL is a low dilution of the sample extract, SH represents the high dilution, and SL is a low dilution of a reference standard.

**The values used to build the graph in Fig. 4 (B):**

**Bioactivity against *Candida albicans***

| **Sample fractions** | **Type** | **Concentration (mg/ml)** | **Inhibition zone (mm)** |
| --- | --- | --- | --- |
| TH | MeOH seed extract | \| 0.30 \| \| --- \| | 25.8 |
| TL | MeOH seed extract | 0.15 | 25.6 |
| SH | Amphotericin (standard) | 0.30 | 26.8 |
| SL | Amphotericin (standard) | 0.15 | 25.9 |

**Table 2.** Anti-microbial activities of MeOH seeds extract of *Vitex negundo* vs amphotericin (standard).

All standard fractions and sample values are mentioned in mg/ml; TH represents the high dilution, TL is a low dilution of the sample extract, SH represents the high dilution, and SL is a low dilution of a reference standard.

**The replicate data used to build the table 2.**

| **1. *Staphylococcus aureus***  **Ciprofloxacin (mm)**  **(28.7 ± 0.5 mm)**  Value 1 = 28.2 (mm)  Value 2 = 28.7 (mm)  Value 3 = 28.9 (mm)  Calculated:  Mean = 28.7 mm  SD ≈ 0.547 mm (≈ 0.5)  **3. *Candida albicans***  **Amphotericin (mm)**  **(27.5 ± 0.4 mm)**  Value 1 = 27.1 (mm)  Value 2 = 27.5 (mm)  Value 3 = 27.9 (mm)  Calculated:  Mean = 27.5 mm  SD ≈ 0.46 mm (≈ 0.4) | **2. Plant seed extract**  **(26.4 ± 0.3 mm)**  Value 1 = 26.1 (mm)  Value 2 = 26.4 (mm)  Value 3 = 26.7 (mm)  Calculated:  Mean = 26.4 mm  SD ≈ 0.35 mm (≈ 0.3)  **4. Plant seed extract**  **(25.7 ± 0.4 mm)**  Value 1 = 25.2 (mm)  Value 2 = 25.7 (mm)  Value 3 = 26.1 (mm)  Calculated:  Mean = 25.7 mm  SD ≈ 0.45 mm (≈ 0.4) |
| --- | --- |
