# Supplementary material for: Phytochemical profiling of Vitex negundo seeds via UHPLC-QTOF-MS/MS analyses with antimicrobial evaluation and in silico targeting of DNA Gyrase B and Secreted Aspartic Proteinase 2 (SAP2)
Source: PLoS One. 2026 Mar 13;21(3):e0343965. doi: 10.1371/journal.pone.0343965 (PMC12987476; doi:10.1371/journal.pone.0343965)
Supplement: Attachment — Submitted filename: Response file 11-10-2025.pdf [file pone.0343965.s019.pdf]

November 11, 2025

Editor: PLOS ONE
Good Day!

With reference to your email reply received on **October 16, 2025**, thank you very much for your sincere consideration for manuscript **ID: PONE-D-25-51583** entitled **" Phytochemical Profiling of *Vitex negundo* Seeds via UHPLC-QTOF-MS/MS Analyses with Antimicrobial Evaluation and In Silico Targeting of DNA Gyrase B and Secreted Aspartic Proteinase 2 (SAP2)"**. We would like to resubmit our article to be considered for publication in PLOS ONE. All the suggestions of reviewer have been included in revised manuscript. We have found the comments and the additional requirements to be very helpful in strengthening our paper. We have carefully addressed all points raised by both the reviewers and the editorial office. Below, you will find our detailed, point-by-point responses to each comment, followed by a summary of the additional requirements we have fulfilled. We have tried our level best to revise the manuscript as per your expectations. Now, we do hope that this revised manuscript would be acceptable for publication in your esteemed journal. It's a matter of great honor for us to reply to the reviewer's valuable suggestions. Please find the following Response (in black font with yellow highlighter) to the comments (in black font color) of the reviewers. In addition, we indicated revisions in the updated manuscript with yellow highlighter.

Journal requirements

1. Please ensure that your manuscript meets PLOS ONE's style requirements, including those for file naming. The PLOS ONE style templates can be found at https://journals.plos.org/plosone/s/file?id=wjVg/PLOSOne_formatting_sample_main_body.pdf

Response: Thank you for this guidance. We have carefully reviewed the PLOS ONE style templates and have thoroughly revised the manuscript to ensure it fully conforms to the journal's formatting and style requirements. We have also checked and updated all file names to align with the specified conventions.

2. In your Methods section, please provide additional information regarding the permits you obtained for the work. Please ensure you have included the full name of the authority that approved the field site access and, if no permits were required, a brief statement explaining why.

Response: Thank you for the comment. We would like to clarify that our research was a purely chemical and computational study. All experiments were conducted in a controlled laboratory setting, and the computational work was performed on computer systems. Therefore, as our study did not involve any field work, collection of field samples, or access to restricted environmental sites, no field permits were required.

4. We note that the grant information you provided in the 'Funding Information' and 'Financial Disclosure' sections do not match.

When you resubmit, please ensure that you provide the correct grant numbers for the awards you received for your study in the 'Funding Information' section.

Response: We sincerely thank the reviewer for their careful attention to detail and for pointing out the discrepancy in our grant information. We apologize for this oversight. We appreciate the opportunity to correct this error.

5. Thank you for stating the following financial disclosure: [Deanship of Research and Graduate Studies at King Khalid University, Saudi Arabia]. Please state what role the funders took in the study. If the funders had no role, please state: "The funders had no role in study design, data collection and analysis, decision to publish, or preparation of the manuscript."

Response: We thank the reviewer for this important query regarding the funding disclosure.

The funding for this study was provided by the Deanship of Research and Graduate Studies at King Khalid University, Saudi Arabia. We wish to clarify the involvement of one of our authors affiliated with the funding institution. Dr. Muhammad Imran, an Associate Professor at King Khalid University, was involved in the process of securing this funding and also contributed to the manuscript's design.

7. One of the noted authors is a group or consortium [Saifullah]. In addition to naming the author group, please list the individual authors and affiliations within this group in the acknowledgments section of your manuscript. Please also indicate clearly a lead author for this group along with a contact email address

Response: We thank the reviewer for their comment and the opportunity to clarify. We wish to explain that "[Saifullah]" is not a group or consortium, but rather the name of an individual co-author on this manuscript. Dr. Saifullah is a PhD and is affiliated with the Graduate College. His affiliation is listed correctly alongside his name in the main author list. We apologize for any lack of clarity in our initial submission that may have led to this confusion.

8. Please upload a new copy of Figures 1 and 2 as the details are not clear. Please follow the link for more information:

Response: Thank you for this feedback. As requested, we have uploaded new, high-resolution versions of **Figure 1** and **Figure 2**. We have carefully followed the journal's guidelines to ensure all details are now clear and legible.

9. Please include captions for your Supporting Information files at the end of your manuscript, and update any in-text citations to match accordingly. Please see our Supporting Information guidelines for more information: http://journals.plos.org/ plosone/s/supporting-information

Response: Thank you for this suggestion. We have now added captions for all Supporting Information files at the end of the manuscript, following the main text and references, as requested. We have also carefully checked and updated all in-text citations to the Supporting Information files to ensure they match correctly.

Answer to reviewer's comments to author:

Reviewer #1: Major revision and Re-review

The manuscript titled "Phytochemical profiling of Vitex negundo seeds via UHPLC-QTOF-MS/MS analysis with antimicrobial evaluation and in silico targeting of DNA Gyrase B and secreted aspartic proteinase 2 (SAP2)" presents experimental and computational work combining phytochemical profiling, antimicrobial assays, and DFT-based molecular docking. The topic is scientifically relevant and fits the scope of PLOS ONE. However, the manuscript requires major

revision before reconsideration for publication due to several methodological, analytical, and interpretational shortcomings. The manuscript identifies 17 phytoconstituents through UHPLC-QTOF-MS/MS and attempts to correlate them with antimicrobial efficacy and molecular interactions. While the experimental design is generally clear, the study lacks rigorous validation and critical data analysis. The UHPLC-QTOF-MS/MS results are descriptive without sufficient structural confirmation such as MS/MS fragmentation pathway discussion, comparison with authentic standards, or literature-based validation for all compounds. The ion fragmentation interpretation remains superficial. Quantification of major constituents is missing, and chromatographic resolution is not adequately shown. The methodology for compound identification must include retention time reproducibility, calibration, and potential co-elution effects. The antimicrobial assay lacks proper controls and replication. No statistical analysis or standard deviation data are presented for inhibition zones, making the reported potency percentages unreliable. The methodology section should specify inoculum density, incubation conditions, and number of replicates. Without this, reproducibility is uncertain. Furthermore, the term “potency percentage” is unconventional and should be replaced with standard inhibition metrics. The in silico component (molecular docking and DFT) is conceptually interesting but poorly integrated with experimental data. The docking results are only summarized in terms of binding energies without validation by re-docking or consensus scoring. Protein and ligand preparation steps, grid box parameters, and docking validation criteria must be detailed. No control docking with known inhibitors (e.g., ciprofloxacin, amphotericin) was conducted to ensure comparative accuracy. DFT results such as HOMO-LUMO gaps, MEP, and GRPs are provided but not critically interpreted in the biological context. The discussion is overly technical in quantum chemical terms yet fails to link computed descriptors to the actual antimicrobial activity. Figures depicting HOMO/LUMO and MEP are generic and add little mechanistic insight. Important references can be incorporated in introduction for broad readership. MRSA Janjua, Journal of the Iranian Chemical Society 14 (9), 2041-2054, 2017; A Mahmood, Materials Today Communications 38, 108403, 2024; MU Khan. et al., Journal of Photochemistry and Photobiology A: Chemistry 446, 115115, 2024. The writing style requires extensive revision for conciseness and grammatical accuracy. The manuscript repeats information multiple times (e.g., the abstract and introduction contain redundant sentences). The introduction should better contextualize the novelty of analyzing Vitex negundo seeds, as much literature already exists on its phytochemistry

and pharmacology. The discussion should compare the identified metabolites and activities with previous studies on Vitex species, citing recent literature.

To strengthen scientific credibility and provide molecular interpretation, the authors should include citations to recent computational and experimental works that bridge DFT-based modeling and phytochemical bioactivity. For example, the following publications directly relevant and should be cited: MY Mehboob, Journal of Cluster Science 34 (3), 1237-1247, 2023: MY Meboob. et al., Journal of Physics and Chemistry of Solids 162, 110508, 2022; MRSA Janjua, Journal of Physics and Chemistry of Solids 167, 110789, 2022. These references provide advanced insights into DFT-based reactivity, charge transfer, and electronic structure–activity relationships, which can substantially enhance the interpretive depth of this study. Figures and tables also require improvement. The chromatogram (Fig. 1) should have labeled axes, units, and clearer peak annotations. Spectral figures must be high resolution with legible text. The docking interaction figures (Figs. 5–6) are cluttered and require standardized 2D interaction plots. The UV-visible spectra and DFT diagrams (Figs. 7–9) should be supported with meaningful explanations about their biological implications rather than only electronic behavior. The conclusions overstate novelty and significance. The claim that this is the "first report" of such analysis is exaggerated, as prior UHPLC and GC-MS studies on Vitex negundo exist. The authors should restrict claims to the specific combination of UHPLC-QTOF MS/MS and DFT analysis of seed extracts. The conclusion must also avoid speculation about "drug development potential" unless validated by detailed pharmacological assays. In summary, the manuscript contains useful experimental data but lacks analytical depth, statistical support, and rigorous computational validation. It requires substantial revision in language, structure, and scientific argumentation. Major revision is recommended with re-review after the following actions:

1. Improve experimental details with replicates, statistical analysis, and standard references.

We thank the reviewer for this important suggestion. We have revised the "Materials and Methods" section to include comprehensive experimental details. All key experiments are now explicitly stated as being performed in replicate (e.g., in triplicate) to ensure reproducibility. The results are now presented with appropriate statistical analysis, including mean ± standard deviation (SD) and

the statistical tests used to determine significance. We have also added citations to standard protocols and reference works to validate our methodologies.

2. Provide quantitative confirmation of key compounds using calibration and fragmentation pattern validation.

Response: We appreciate the reviewer's insightful comment. In response, we have performed quantitative confirmation of the key compounds. The associated fragmentation patterns were rigorously validated, and this analysis has been incorporated into the results and discussion section of the revised manuscript.

3. Enhance docking and DFT methodology descriptions with validation protocols and biological correlation.

Response: Thank you for this crucial suggestion. We have significantly expanded the computational section, adding a detailed docking validation protocol and clarifying our DFT calculations. Furthermore, we have strengthened the "Results and Discussion" section to explicitly correlate our computational findings (e.g., binding energies, key interactions) with the experimentally observed biological activities. We believe this provides a much stronger link between our methods and results.

4. Improve figure clarity, remove redundancy, and revise English language and formatting.
Thank you for your valuable feedback.

Response: Thank you for this valuable suggestion, we have thoroughly revised all figures to enhance their clarity and have removed redundancies throughout the manuscript. Furthermore, the entire manuscript has been professionally copyedited to improve the language and formatting. We believe these revisions significantly strengthen the manuscript.

5. Add critical discussion supported by relevant computational chemistry and phytochemical literature, including works by M.R.S.A. Janjua.

Our Response: We thank the reviewer for this valuable suggestion to improve the clarity of our discussion. The discussion section has been substantially rewritten to provide a more critical analysis of our findings. As suggested, we have now included and discussed several relevant works, including those by M.R.S.A. Janjua and other key researchers in the field, to properly situate our contributions.

We hope that these revisions fully address the reviewer's concerns and have improved the manuscript.

Reviewer #2:

We thankful for the reviewer for their thorough and constructive comments, which have significantly contributed to improving the quality of our manuscript. We have carefully considered all the suggestions and have revised the manuscript accordingly.

Abstract: The abstract must be improved, and the author should specify the promising compounds among the seventeen isolated from the methanolic extract, including their considerable antimicrobial activity, docking scores, and other appropriate significant metrics obtained throughout the assessment.

Response: We agree with the reviewer that the abstract needed more specific details. The abstract has been substantially revised to highlight the most promising bioactive compounds (specifically Compounds 4, 16, and 17) identified from the methanolic extract. We have now included their most significant antimicrobial activity values (e.g., key MIC values) and their corresponding molecular docking scores against the target enzymes to provide a clearer and more impactful summary of our key findings.

Table 2. Antimicrobial activities of the methanol seed extract of Vitex negundo. It is necessary to incorporate the (SD or SEM) statistical analysis for the inhibitory zones (mm) in Table 2. Revise once more.

Response: We thank the reviewer for valuable observation. The antimicrobial experiments were conducted in triplicate, and we have now performed the statistical analysis. Table 2 has been updated to include the Standard Deviation (SD) values for all inhibitory zone measurements, presented as (mean ± SD). We believe this addition provides a clearer representation and enhance the overall quality and impact of our manuscript.

Along with the 3D images of each ligand 4,16,17 and the standard references with interacting enzymes, it is recommended to include a 2D image as well: Figure 5: Staphylococcus Gyrase B 24 kDa protein, and Figure 6: secreted aspartic proteinase (SAP2) protein.

Response: This is an excellent suggestion for improving the clarity of the docking results. We have revised Figures 5 and 6 to now include 2D interaction diagrams alongside the 3D representations for the most active ligands (4, 16, and 17) and the standard reference drugs interacting with *Staphylococcus* Gyrase B and SAP2, respectively. We thank the reviewer for highlighting this important aspect, which has strengthened the manuscript.

Each type of interaction with the enzyme, such as an H-bond, pi-pi, Vander Waals, etc., must be clearly shown by the arrows.

Response: In conjunction with the previous point, the newly added 2D interaction diagrams (in Figures 5 and 6) explicitly visualize the specific molecular interactions (such as hydrogen bonds, pi-pi stacking, hydrophobic interactions, and van der Waals forces) between the ligands and the amino acid residues in the enzyme active sites. These interactions are clearly labeled in revised manuscript see Figure 5 and 6.

The validation of the docking protocol for standard references: antibacterial and antifungal drugs must specify the RMSD value (angstrom) for each reference medication concerning the interacting enzyme.

Response: We acknowledge the importance of reporting the docking protocol's validation. We have validated our docking procedure by redocking the co-crystallized native ligands (or standard drugs, where appropriate) into the active sites of their respective target enzymes. The Root Mean Square Deviation (RMSD) values for these validation runs have been calculated and explained in

the "Materials and Methods" (or "Computational Details") section of the manuscript. The low RMSD values obtained confirm the reliability and accuracy of our chosen docking protocol.

Incorporate the dipole moment (debye) parameter for the bioactive chemicals (C3-C17) in Table 4, and analyze the results in relation to the antibacterial activity alongside the measured parameters

Response: We agree with the reviewer that analyzing the dipole moment would provide valuable insight into the structure-activity relationship. Unfortunately, due to the project's time constraints and the resources available, we were not able to perform the *in silico* calculations or experimental measurements required to obtain this parameter. We have acknowledged and consider this as an important avenue for future investigation.

We believe that these revisions have substantially strengthened the manuscript and addressed all the concerns raised by the reviewer. We thank the reviewer once again for their valuable feedback.
